# Using analogous sectors to build good practice resources in regulation of urban faecal sludge emptying and transport

**DOI:** 10.1039/d5ew01108f

**Published:** 2026-07-22

**Authors:** Claire Grisaffi, Paul Leinster, Alesia Ofori, Sandy Mae A. Gaspay, Florence Muheirwe, Alison Parker

**Affiliations:** a School of Civil Engineering, University of Leeds UK c.grisaffi@leeds.ac.uk; b Faculty of Engineering and Applied Science, Cranfield University UK paul@pierconsultants.co.uk; c Faculty of Engineering and Applied Science, Cranfield University UK alesia.ofori@cranfield.ac.uk; d University of the Philippines Institute of Civil Engineering, Traffic Engineering and Management Group – UP National Centre for Transportation Studies Philippines sagaspay1@up.edu.ph; e Institute of Human Settlements Studies, Ardhi University Tanzania muheirweflorah@gmail.com; f Faculty of Engineering and Applied Science, College Road, Cranfield University MK43 0AL UK a.parker@cranfield.ac.uk +44 (0)1234 758120

## Abstract

In eastern and southern Africa, faecal sludge emptying and transport is the dominant urban sanitation solution, mainly delivered by a diverse and largely informal private sector. While regulation is advancing, there is limited evidence on how to build compliance among service providers at scale. This article explores learning from six analogous sectors – street food vending, informal transport, artisanal mining, alternative water supply, construction waste management, and municipal waste pickers – to inform effective regulatory practices. A rapid literature review (220 publications) and expert input (24 sector specialists) were combined with 19 key informant interviews with regional sanitation regulators. The findings highlight emerging good practices: making compliance clear, simple, visible, and valued; understanding service providers' economic and social and normative drivers; and using participatory, adaptive regulation. Key insights include the need to move beyond binary views of informality, tailor policies to diverse provider types, and work effectively through associations. Limitations of information-based tools like vehicle tracking were also noted. Recommendations for a more inclusive regulatory approach are proposed.

## Abstract 2

Inclusive regulation for all service providers: segment providers by capacity and willingness to comply and adapt responses. Regulations should include informal providers as legitimate actors rather than problems to be formalized.

## Graphical abstract

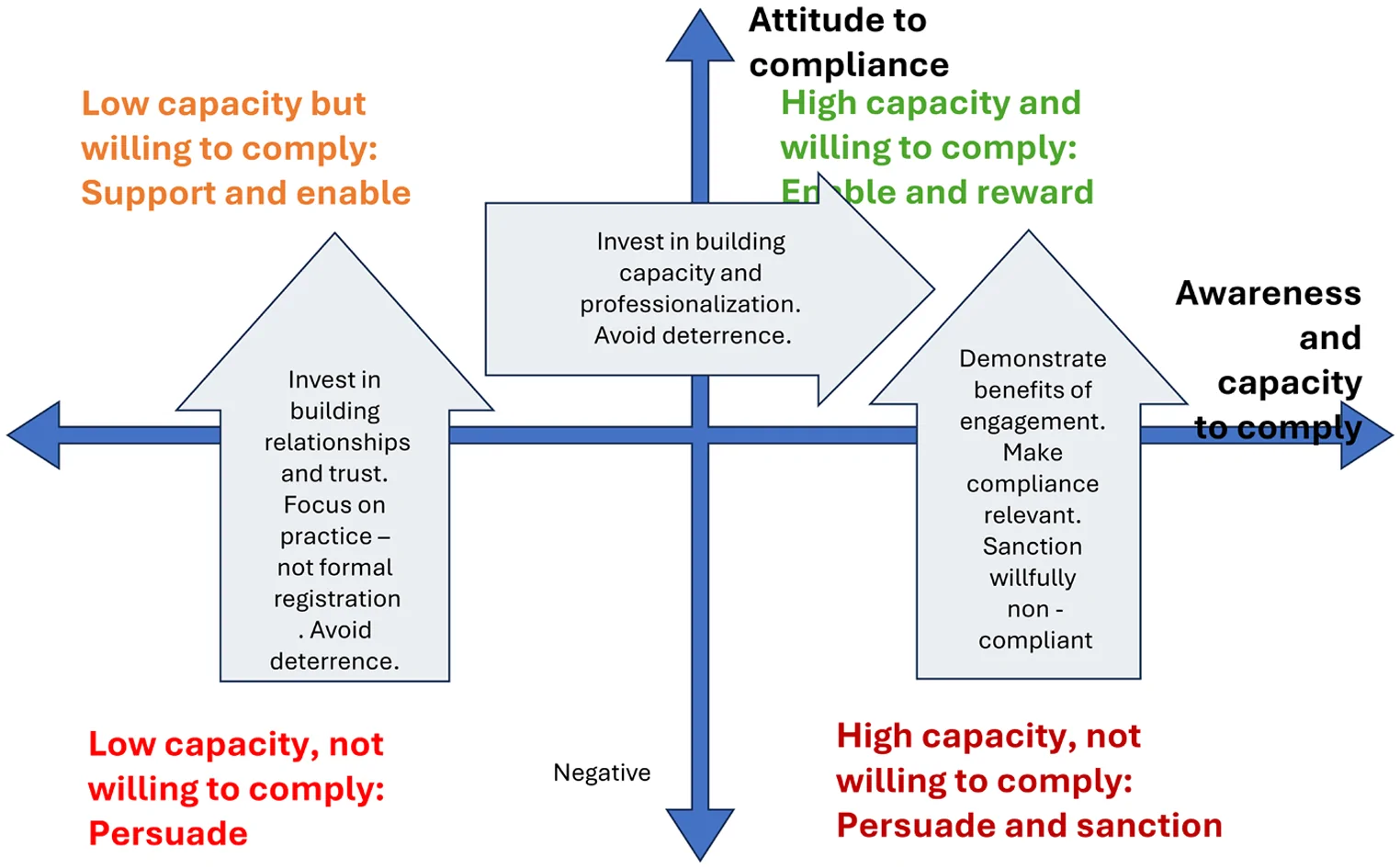

**Water impact**

Effective regulation is key to scaling safe urban sanitation and public health and the environment. This article addresses how to build compliance among fecal sludge emptying and transport providers in eastern and southern Africa. Drawing lessons from six analogous sectors, the study proposes inclusive, adaptive, and participatory regulation, recognition of informal providers, and segregating service providers by capacity and attitude.

## Introduction

Urban sanitation is a global challenge; more than 1.5 billion urban residents do not have access to safely managed sanitation.^1^ In 81 countries, predominantly in sub-Saharan Africa and south and eastern Asia, faecal sludge emptying and transport is the majority urban sanitation solution.^1^ Unsafe disposal of faecal sludge is a substantial contributor to unsafe sanitation, impacting predominantly on low-income areas and damaging public health and the environment.^2,3^

Faecal sludge emptying and transport services across eastern and southern Africa are commonly provided by a heterogenous private sector, ranging from established vacuum tanker operators to individuals servicing their community with buckets.^4^ The mandated service authority, the utility or municipal department at the city level, may be contracting some services, or providing services directly. However, providing citywide sanitation requires mandated service authorities to also take on the role of a local regulator, translating national regulations into action.^5^

In this article regulation is defined as a process of effecting change, led by regulators who work through, and with, a wide range of actors.^6^ Regulation which builds voluntary complianceis likely to be more effective than requiring resource intensive enforcement, particularly for services such as faecal sludge emptying and transport which are characterised as low visibility, highly dispersed and highly informal.^7–9^

There are a growing number of peer-reviewed publications and practitioner guidance addressing regulation of faecal sludge emptying and transport, often as part of citywide inclusive sanitation.^10–13^ Implementation is nascent and evidenced good practice of building compliance with safe practices in faecal sludge emptying and transport is scant.^14^ However, there are multiple sectors which face similar challenges in building compliance of front-line service providers who are low visibility, highly dispersed, and highly informal. Recognizing these commonalities and drawing on experience from these sectors may provide a more substantial evidence base around what has worked, or not, in building compliance. This learning could then inform research and development in faecal sludge emptying and transport, to identify practices which may be more effective and provide early warning of risks or potential negative impacts.

Cross-sector learning is not a simple process due to differing language, basic assumptions and making tacit knowledge explicit.^15,16^ However, the benefits of breaking sectoral silos in addressing these complex challenges are widely recognized in and outside the water and sanitation sector.^17,18^ To date there has not been a comprehensive review linking cross-sector learning to building compliance with safe practices in faecal sludge emptying and transport.

This article aims to generate insights from analogous sectors to inform emerging practice in regulation of faecal sludge emptying and transport. The focus is on city level mandated service authorities and how they can work more effectively to build compliance of private service providers. We identify good practices in building compliance for further research and potential policy recommendations for mandated service authorities working as *de facto* local regulators, with the support of national regulators.

## Materials and methods

**Fig. 1:**
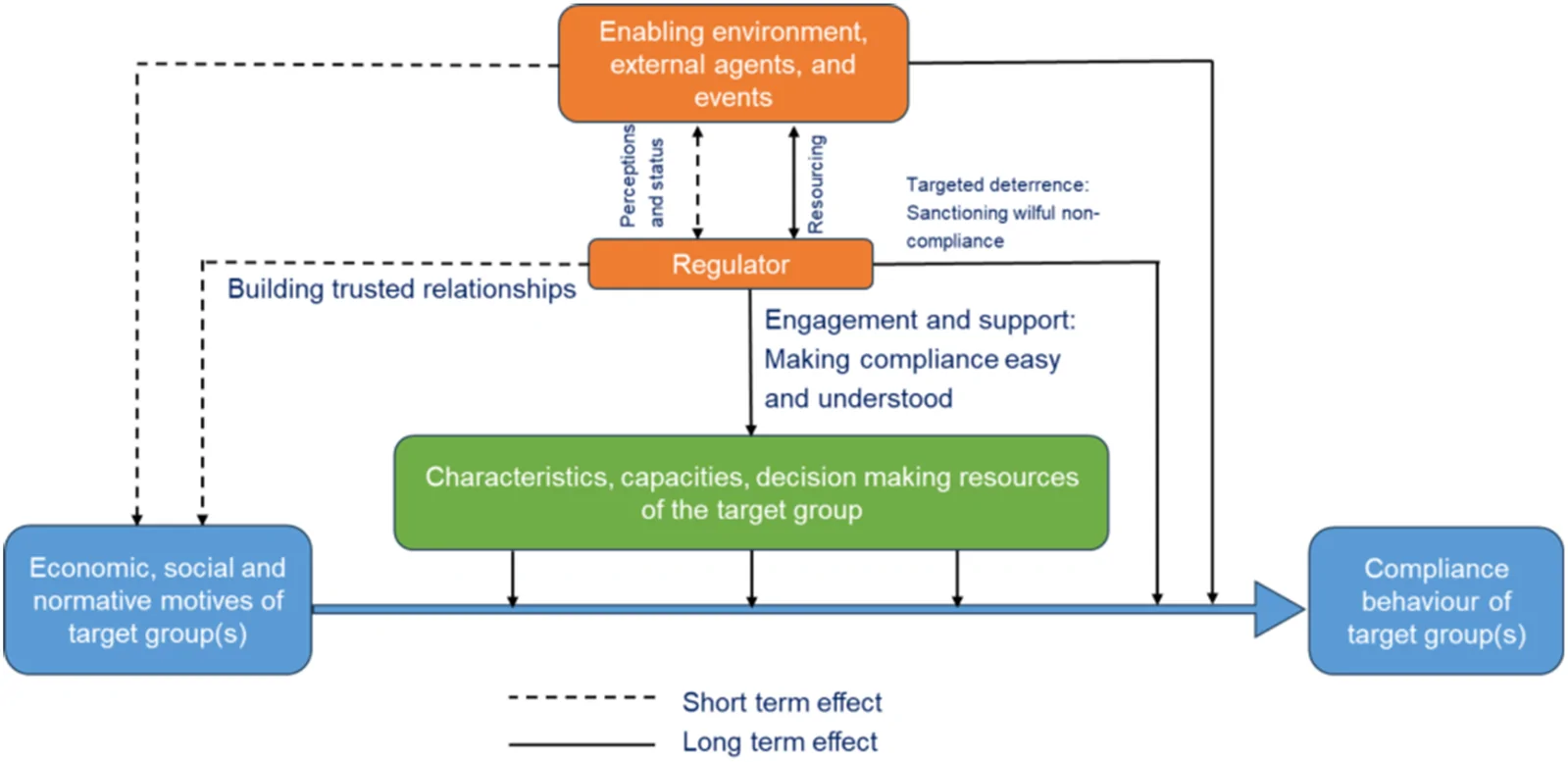
Adapted holistic compliance model.^14^

This article builds directly from the priority challenges to building compliance, and the adaption of a holistic model of compliance for faecal sludge emptying and transport service providers given in Grisaffi *et al.*^14^ The adapted model is given in Fig. 1.

The research followed a stepped process. Firstly, emerging good practice in building compliance of faecal sludge emptying and transport service providers was identified through a rapid literature review and an analysis of key informant interviews with national water and sanitation regulators and mandated service authorities. Secondly, sectors analogous to faecal sludge emptying and transport were short-listed. A rapid literature review was then completed of the six shortlisted analogous sectors and validated by sector specialists. Finally, the findings for the analogous sectors and faecal sludge emptying and transport were compared using the adapted holistic model of compliance.

### Identifying emerging good practice in building compliance of faecal sludge emptying and transport service providers

The rapid literature review started from the literature reviews completed by Grisaffi *et al.*^14,19^ These were updated using peer reviewed literature as described in S2 and organisational literature from the Sanitation Workers Knowledge and Learning Hub, and the member organisations of the Initiative for Sanitation Workers, for a more complete picture of emerging practice. In total 15 papers were identified and coded.

This rapid literature review was supplemented by an analysis of 19 key informant interviews completed with leading national water and sanitation regulators and mandated service authorities from eastern and southern Africa. The data collection and profile for these 19 key informant interviews are described in detail in Grisaffi *et al.*^19^ The sample includes 10 national regulators and 9 mandated service authorities from across 5 countries. The transcripts were analysed for the current paper looking for current or proposed good practice using the elements of the adapted holistic model of compliance. The findings from the transcripts are summarised against the elements of the holistic model for compliance in Table 1. Where the transcripts directly support the point being made they are noted as in the citations as ‘KII’. Otherwise, the reflections from key informants are discussed directly after the comparison of the literature.

**Table 1:** Themes from cross-sector literature review and key informant interviews with national regulators and mandated service authorities in faecal sludge emptying and transport

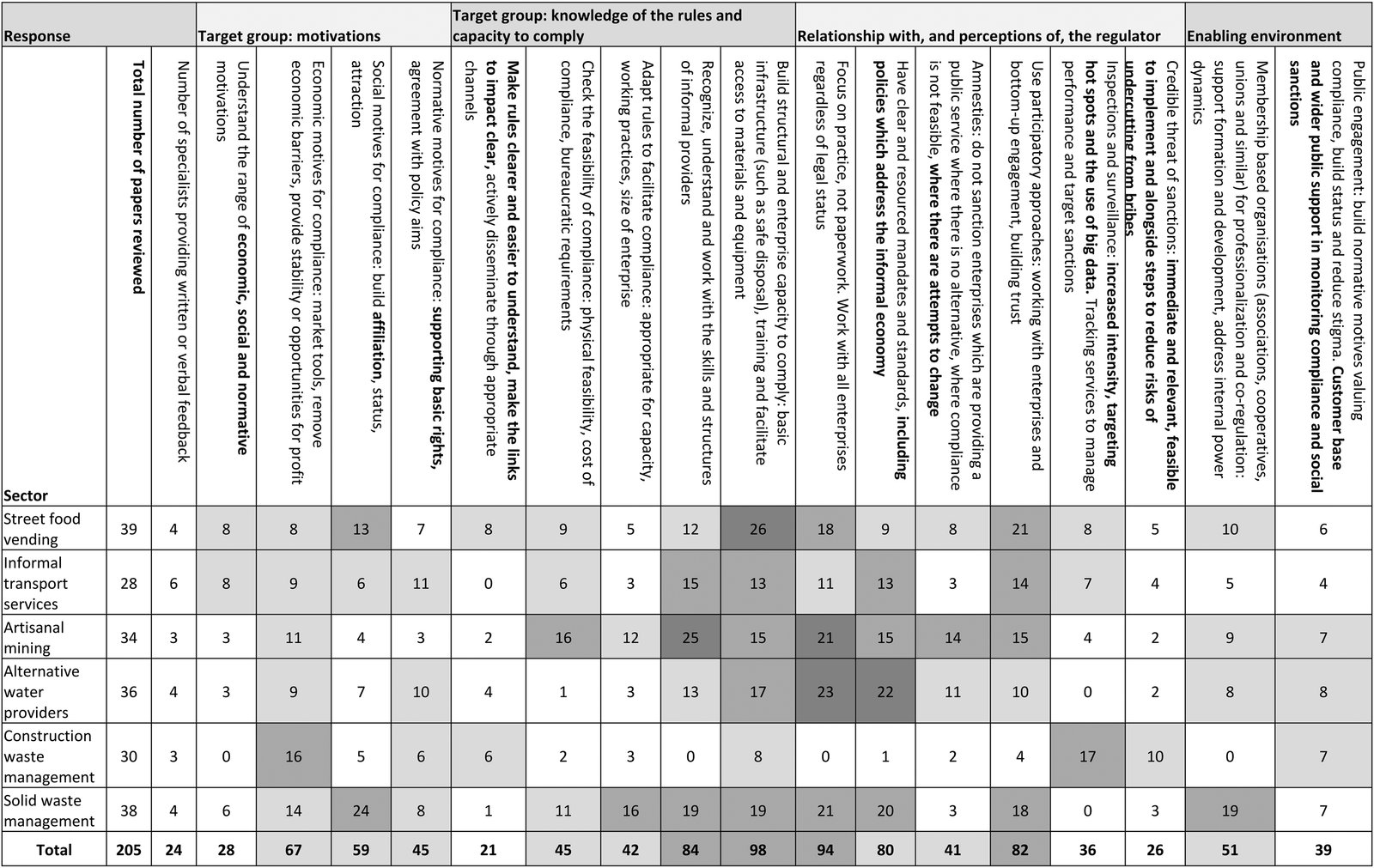 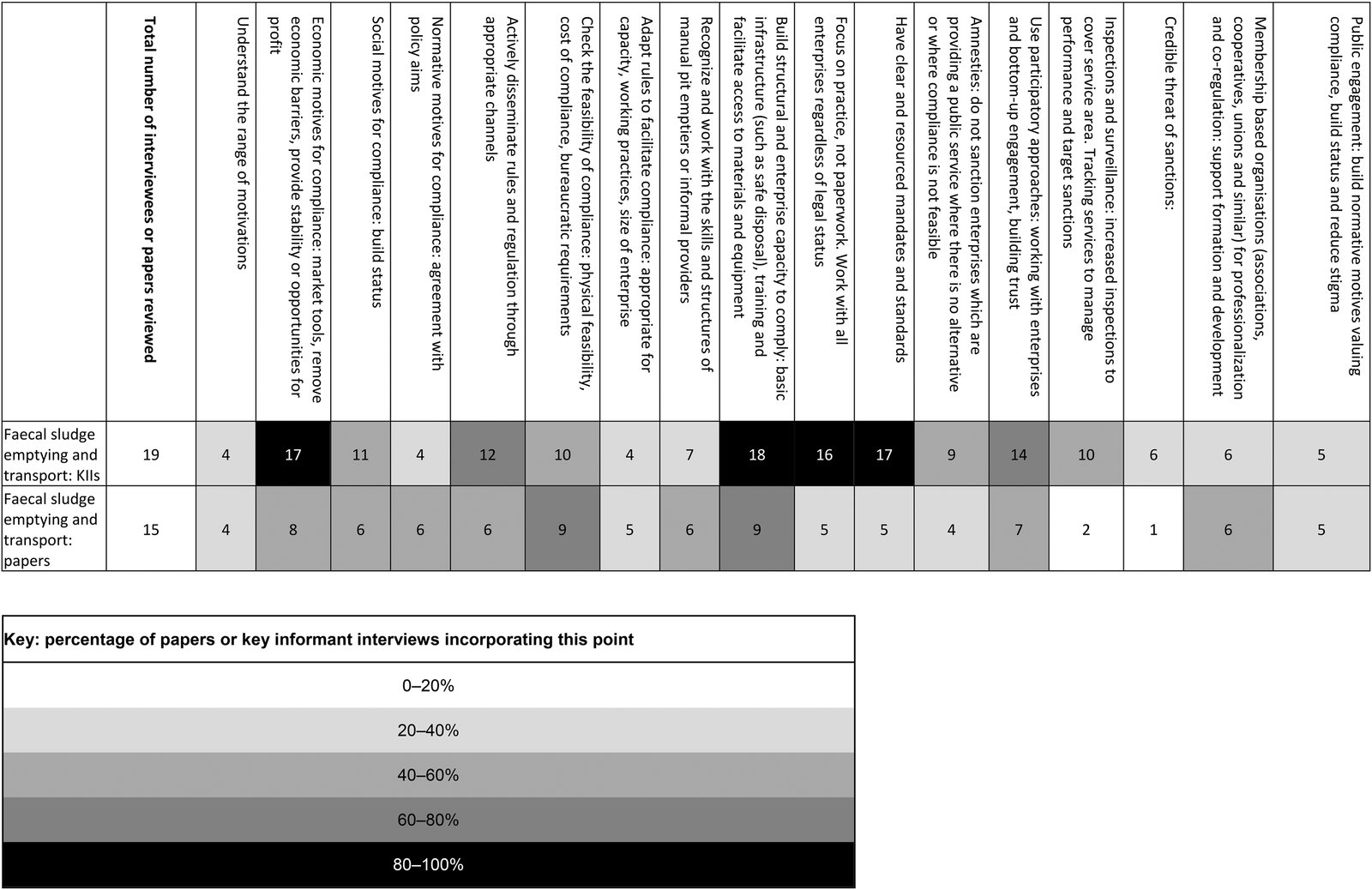

### Learning from analogous sectors

**Fig. 2:**
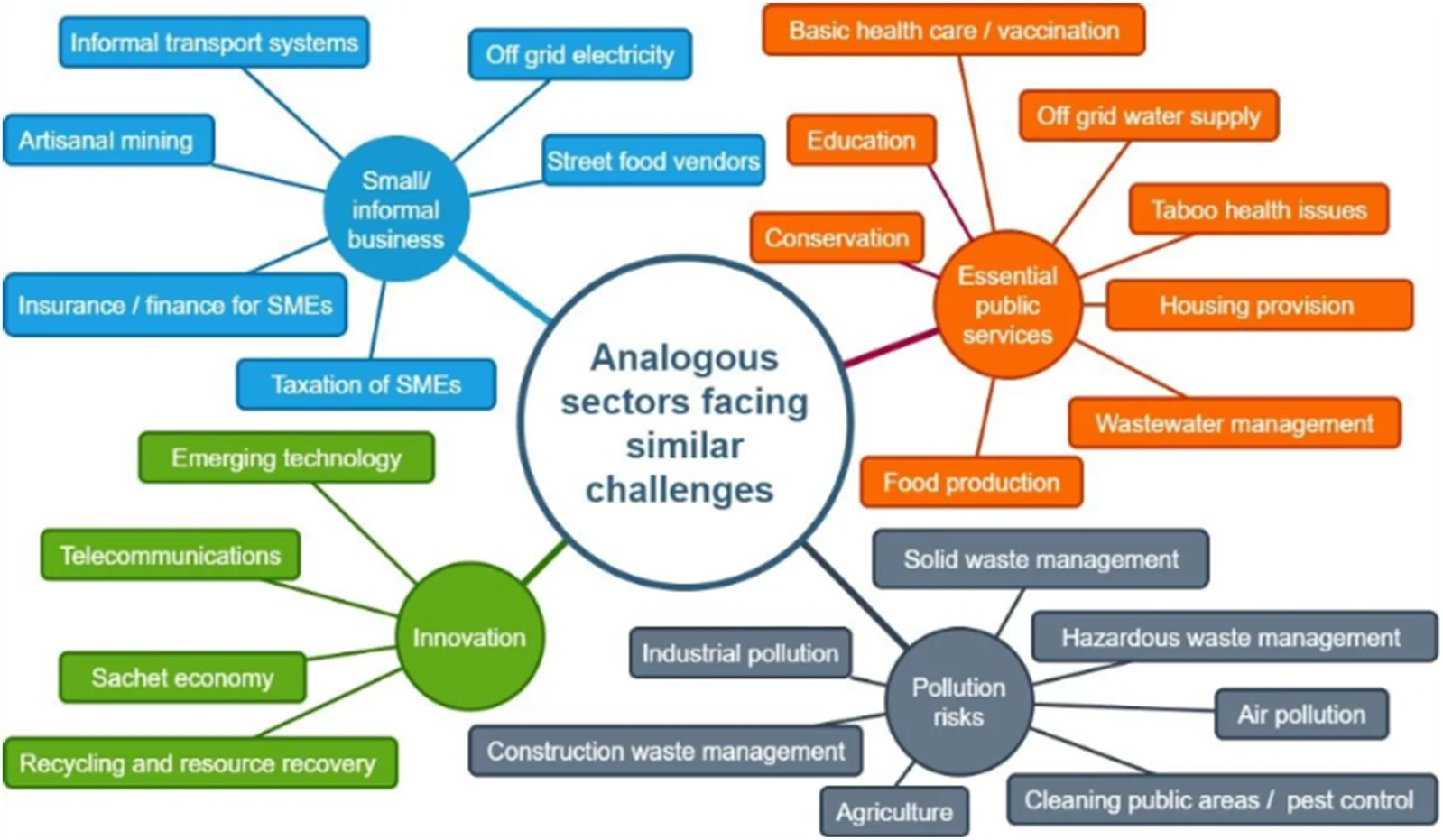
Potential analogous sectors.^14^

A longlist of 25 potential analogous sectors was identified by an expert panel through an online Delphi study. The full list of grouped analogous sectors is given in Fig. 2. The description of the expert panel and Delphi study process is given in Grisaffi *et al.*^14^ Sectors which faced the four priority challenges in building compliance in service providers, were shortlisted; low levels of trust between mandated service authorities and service providers, the view of the service as low status (possibly disgusting) impacting managers and front-line staff, the costs of compliance for service providers, and the enabling environment. This process is described in S1. Trust is defined in this study as ‘*a willingness to be vulnerable based on positive expectations of the other*’.^14^

Six analogous sectors were identified for further review in the current paper, based on facing these four challenges in building compliance. These sectors are (i) street food vending, (ii) informal transport services, (iii) artisanal mining, (iv) alternative water providers, (v) construction waste management (fly tipping), and (vi) solid waste management (waste pickers).

A rapid review was completed for these six sectors, as detailed in S2. The rapid review ‘*accelerates the process of conducting a traditional systematic review through streamlining… to produce evidence in a resource-efficient manner*’.^20^ The main areas of streamlining were to replace multiple database screening with the use of online literature mapping tools, to narrow the range of keywords and to reduce quality checks. Searches were completed on two databases, Scopus and Google Scholar^21^ and screened for relevance. At least 5 relevant seed papers identified by full text review were then used in two online literature mapping tools to identify further papers; Inciteful^22^ and Research Rabbit.^23^ The generated long list was manually screened.

**Fig. 3:**
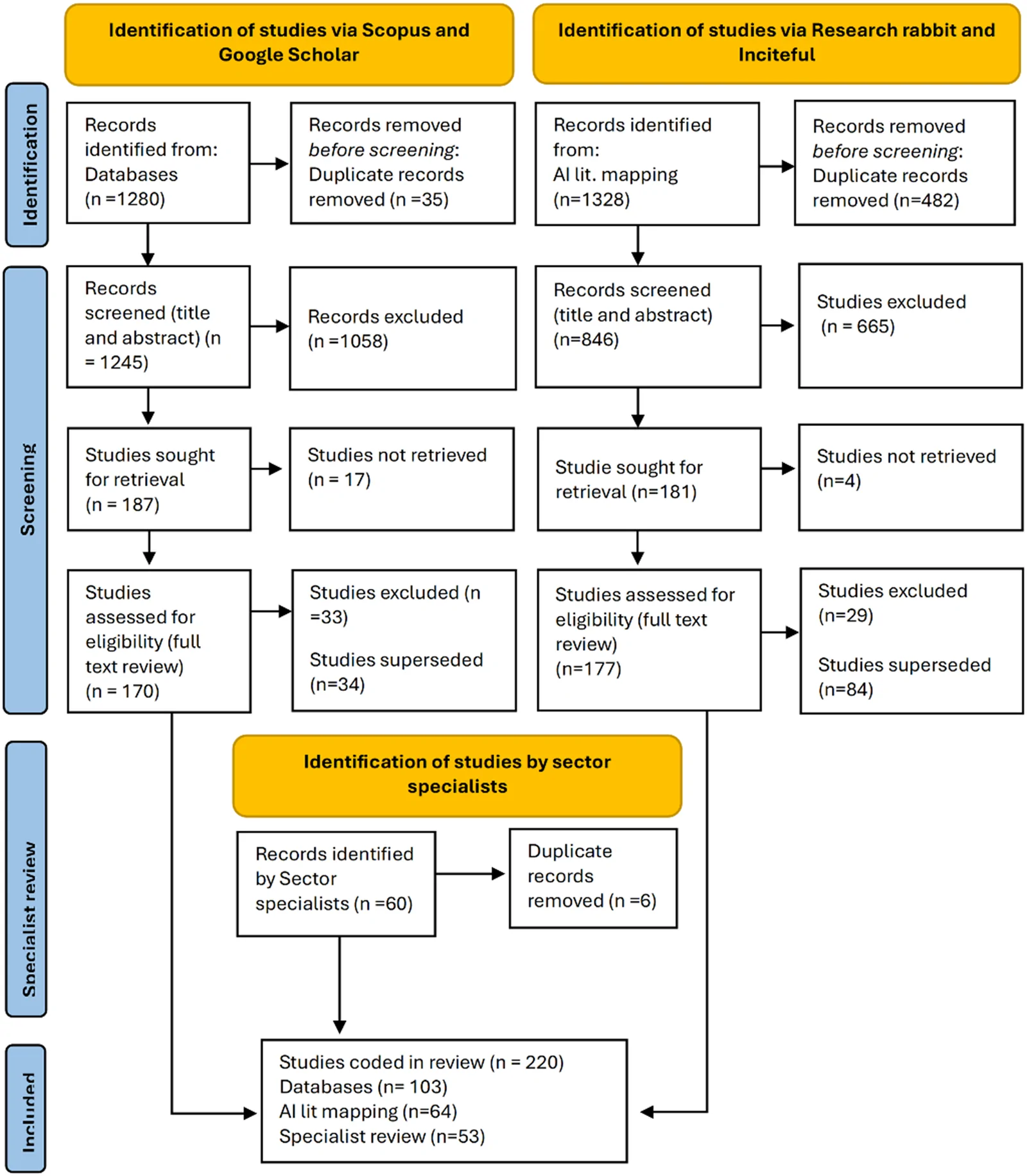
PRISMA diagram for the overall rapid review process.

A full text review was completed of all relevant papers. A sub-set of these papers were coded, starting with recent review articles and then layering on more recent primary articles until data saturation was reached, taken as the point where no new insights are being gained. The remaining eligible papers were rapidly screened to check for new insights and if none were noted they were marked as superseded. The number of papers marked as superseded are given in Fig. 3. Coding used the elements of the adapted holistic model of compliance given in Fig. 1; (i) target groups' economic, social and normative motivations; (ii) target groups' knowledge of the rules and capacity to comply; (iii) (local) regulator capacity and relationship with the target group and (iv) the enabling environment and external actors. In total 205 papers from analogous sectors were identified and coded. The total number of papers coded from each sector is given in Table 1.

Overall, 220 papers from faecal sludge emptying and transport service providers and analogous sectors were identified and coded. The flow of the rapid review is given in the summarised PRISMA diagram in Fig. 3. This figure shows the overall number of papers identified through databases, online mapping tools and from specialist recommendations. The details per sector are given in S2.

The narrative summary generated for each sector was shared with the authors cited in the rapid literature review. Invitations to comment on completeness and accuracy were extended to the corresponding, first and second authors until at least three reviews were received for each sector. Invitations were sent over email and only initial invitations were sent, there was no follow-up over email or other platforms. This choice was made to avoid antagonising academics who may already be inundated with an excess of review requests. However, it did mean that responses were received only from those specialists whose curiosity was instantly piqued by considering how their knowledge might be relevant to faecal sludge. Response rates were understandably low, around 10% overall. In total 24 specialists provided written or verbal feedback, including additional papers to incorporate, comments and clarifications. Reviewers also commented on the overall body of literature and challenges or trends in the sector which are not reflected in the literature. There were no identifiable trends in gender, seniority or geographic location in respondents compared to non-respondents.

**Fig. 4:**
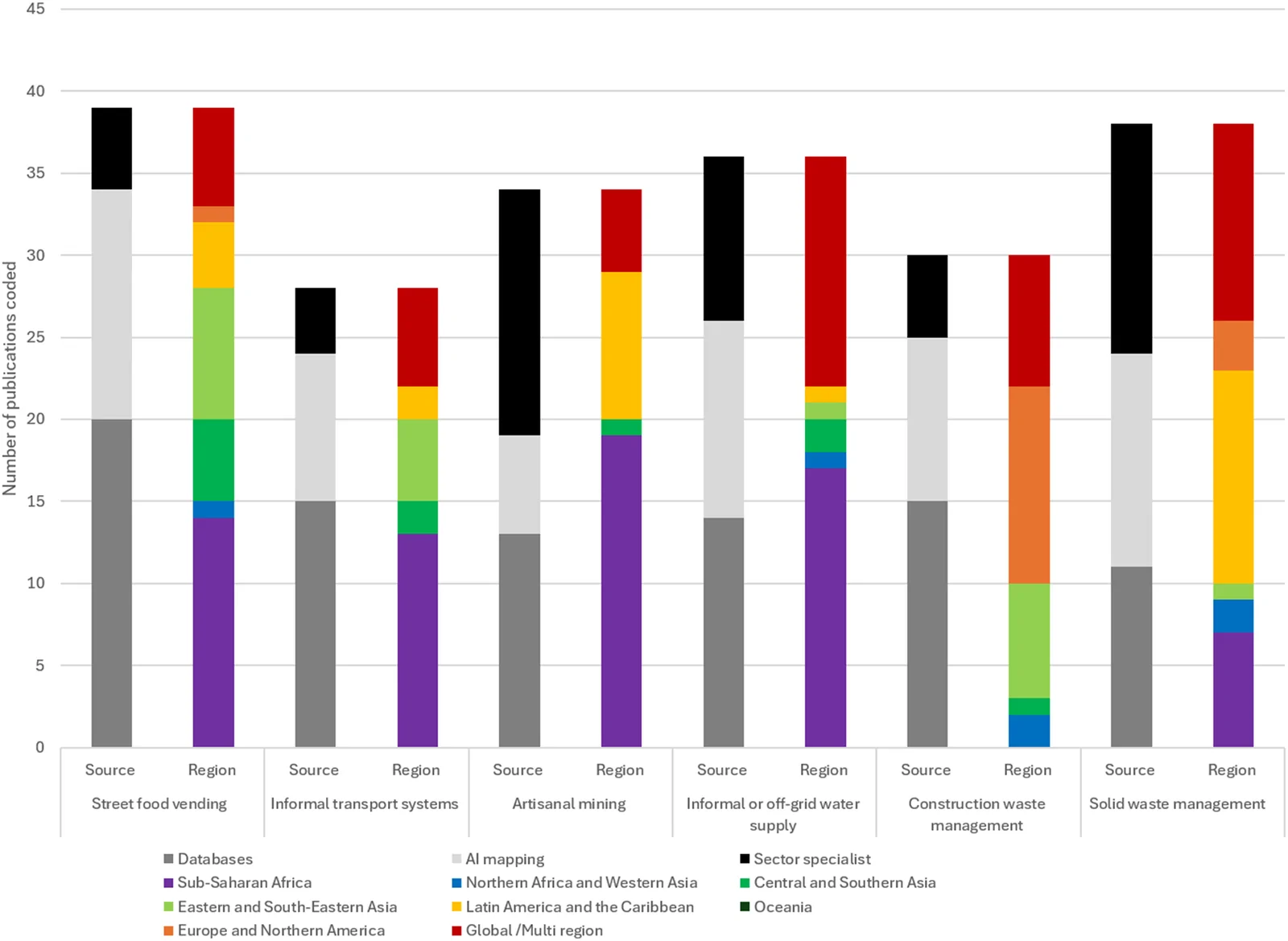
Literature reviewed for each sector, split by source and geography.

Feedback was incorporated into the final sector summaries given in S3. Feedback which could not be addressed through the rapid review, were included as limitations. The profile of the complete set of literature reviewed, including the geographic distribution and the breakdown by source is given in Fig. 4.

The respondents were all assured of confidentiality and informed of their right to remove their data in line with ethical approvals obtained from Cranfield University Research Ethics CURES/17815/2023 (updated reference CURES/26665/2025). The final sector summaries and the anonymised specialist feedback was shared with all respondents alongside the draft manuscript. Responses, all positive overall with some constructive comments and additions, were received from 71% of respondents.

These six sector summaries were synthesised. The synthesis considers only those findings which are identified across two or more sectors. Particularly extensive literature on a relevant subject from one sector is also highlighted.

### Comparison of the analogous sectors and faecal sludge emptying and transport

The findings from the analogous sector review were then compared to emerging practices for regulation of faecal sludge emptying and transport. Conflicting, confirming and novel elements were identified. Given the high level of commonality all sectors are often referenced as a group. Where a finding is relevant to analogous sectors only this is specified. Findings are linked to specific sectors where not applicable to all analogous sectors or all sectors including faecal sludge emptying and transport. For readability only example references are taken in the text, the full set of literature is given in S3. In the synthesis ‘*target groups*’ is used to mean individuals or enterprises subject to regulation.

The main emerging themes from each sector are given in Table 1, including the number of papers where the point is raised as a finding, a challenge to be addressed, or a recommendation. Table 1 is structured by elements of the adapted holistic model of compliance, broken down further where needed for clarity. The framework of the compliance model also provided the framework for determining what learning was included for consideration in the review process. All learning which linked directly to an element in the compliance framework was coded. If it fell outside of this framework then it was considered not to be relevant to compliance building. Two co-authors completed spot checks during the screening process and review the narrative summaries. Areas where analogous sectors appear to add to emerging good practice in faecal sludge emptying and transport are included as bold text in Table 1.

The synthesis flattens difference and emphasizes common experience. An additional analysis identifying relevant sector specific insights was therefore completed and is given in Table 2.

**Table 2:** Additional sector specific insights relevant to regulation of faecal sludge emptying and transport

Sector	Insights from sector summaries
Street food vending	• Trying to relocate, or artificially fix, working areas has led to wasted infrastructure investment. Instead provide (possibly temporary or mobile) infrastructure based on the constraints and requirements of street food vendors
	• Co-regulation, through zoning or associations, may exacerbate power dynamics between street vendors. More vulnerable vendors may be exploited or further marginalized unless these dynamics are understood and protected against
Informal transport services	• Ride hailing applications can provide useful routing information. However, information-based regulation using digitized ride hailing and service tracking are typically a regressive form of regulation which pushes down drivers wages and increases costs to vulnerable households, by removing informal cross-subsidies facilitated by drivers and disrupting other informal transport modes. It may marginalize vulnerable groups, both drivers and customers
	• Compliance costs need to be fully costed, and the level of support understood. There are repeat examples of new requirements being introduced, such as upgraded vehicles, new payment modalities and online tracking which were either abandoned or circumvented by target groups
	• Commission based systems and lack of collective support and representation drive unsafe behavior, by increasing precarity of livelihoods and incentivizing speed rather than quality of service. Better treated drivers provide better services
Artisanal mining	• Policies need to have differentiated formalization requirements for different mining groups, from artisanal to large scale mining. This approach may mean accepting continued negative externalities as part of bringing miners on board and having a transparent process of improvement
	• ‘*Formality*’ and ‘*formalization*’ are not neutral terms; they are politically loaded and can be weaponized to deliberately scapegoat and control market access for certain groups by creating barriers to entry
Alternative water providers	• The moral economy – framing economic activity through the lens of ethical norms, social values, and customary practices – can facilitate self-regulation when there are strong normative beliefs around the service provided by the target group. Normative beliefs in the right to water ‘*facilitate practices that keep water prices affordable and water distribution seen as fair*’
	• Accepting off-grid water supply may mean consigning certain groups to lower standards of service. Some water service providers are actively criminal; providing poor quality water at exploitative prices to communities with no alternative
Construction waste management	• Proximate dump sites are identified as only a local modifier of behavior. Without wider interventions individuals are likely to use the most convenient (legal or illegal) disposal sites. Improved urban planning is needed to increase visibility of disposal. Wider urban development could increase the ability and likelihood of communities to report and prevent illegal disposal
	• Satellite imagery and big data – for example the use of indices on land surface temperature and vegetation – can be an effective approach for identifying significant disposal hot spots
Solid waste management	• Recognizing grassroots innovation, and adapting to existing grassroots systems may be both more cost effective and enabling than integrating workers into municipal systems
	• Membership based organisations can be instrumental in building the sector, benefiting waste pickers, the environment and the economy, however they can also lead to further exploitation and marginalization of vulnerable members
	• Gaining and retaining support for stigmatized groups requires continuous commitment and resourcing, it is quickly derailed by political changes or the entrance of corporate actors

## Results and discussion

**Fig. 5:**
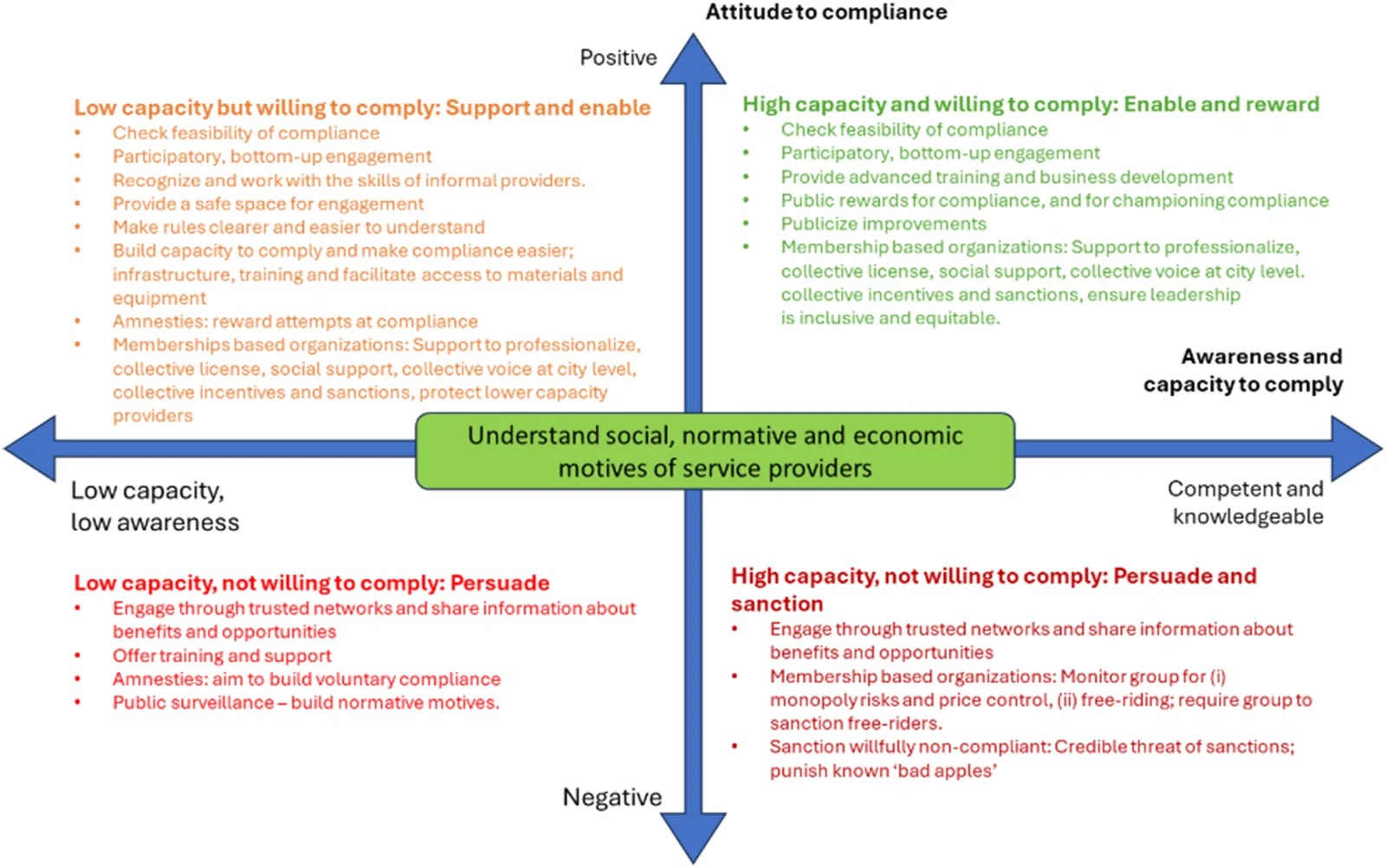
Summary of good practice identified through the review.

An overview of the analogous sectors reviewed is given below. The sub-section ‘*Reinforcing established good practice*’ gives a summary of good practice which supported by evidence from both the analogous sectors and faecal sludge emptying and transport. The following sub-sections are then structured by four themes where the analogous sector review appears to extend good practice in faecal sludge emptying and transport: (i) behavioural drivers; (ii) adapting rules and regulations to make compliance feasible; (iii) understanding and process of engagement of the informal economy; and (iv) developing and regulating through membership-based organisations. The good practice identified through this review are summarized in Fig. 5. This combined results and discussion section is closed with a review of the validity of the method used and its usefulness in generating new insights for building compliance of faecal sludge emptying and transport service providers.

### Sectors reviewed

The analogous sectors are all similar to faecal sludge emptying and transport in being highly informal and dispersed and having significant externalities. Front line workers all face a level of stigma. The cost of compliance and distrust between the target group and the regulator is a common factor.

Street food vendors can range from sizeable formal enterprises to individuals armed with only a saucepan. Street food vendors are providing an important public service of affordable food for large numbers of migrant and transitory workers in urban areas.^24^ However, poor hygiene practices of street food vendors are a significant contributor to foodborne illness.^25^ Street food vending offers an important livelihoods opportunity and has continued to operate despite being illegal and state-oppressed in many settings.^26^

Informal transport service providers range from large minibus operators to individuals with motorbikes.^27^ Informal transport services provide affordable mobility in and around cities, jobs for low-skilled workers and are an essential service connecting less accessible or low-income areas to public transport networks.^27^ However, there are associated public health risks, including air and noise pollution and a high level of traffic accidents.^27^

Artisanal and small-scale mining, referred to here as artisanal mining, is labour intensive and uses simple technology and minimal mechanization.^28^ It is a significant livelihood option in most mineral economies offering much greater incomes than other occupations.^29^ The vast majority (80–90%) of artisanal miners are working informally.^30^ Poor practices in the sector lead to significant environmental degradation, however there are frequently no feasible compliant alternatives for artisanal miners.^31^

Alternative water providers include all commercial water providers outside of the networked system; tankered water, water vendors, small networks reselling bulk water and other forms of modular and decentralised water systems.^32^ Alternative water providers are the main water service provider for at least 700 million urban residents, providing an essential public service where utilities are absent or services are unreliable.^33^

Construction waste management is the reducing, reusing, recycling, and disposal of waste materials generated during construction, renovation, and demolition. Waste reduction and reuse have substantially addressed many of the challenges in construction waste management.^34^ This review has focused on fly tipping, which remains a pervasive problem in construction waste management and is low visibility and highly dispersed.^35^ Learning from the incentive structures for reuse and recycling in construction waste management could be relevant for the reuse of faecal sludge, however it is considered outside of the scope for this article. Published research on fly-tipping in construction waste management has quite a different profile to the other analogous sectors, being largely based in high-income countries and being more located within formal industry. Construction waste management is retained in this review because of the importance of unsafe disposal in faecal sludge emptying and transport and the potential for learning around compliance building in this area.

Solid waste management in municipalities is underpinned by waste pickers making a livelihood from providing an essential public service collecting, sorting and processing municipal solid waste.^36^ Waste pickers remain some of the most stigmatized and marginalized workers in society, however waste picker organisations are also recognized as being highly innovative and developing environmental and social solutions from the grassroots.^36^

### Reinforcing established good practice

Across all sectors knowledge and understanding of regulations are a basic step in building compliance and are linked to improved practices.^13,37–42^^,^††This denotes transcripts which directly support the point being made. The important role of the regulator or mandated service authority in facilitating this knowledge and understanding through clear communication of regulations, active dissemination and training is recognized.^13,37,43^^,^† Making regulations understood, includes also an understanding of the benefits and for the regulation to be perceived as straightforward.^43–47^ However, the impact may be negligible without wider interventions to enable compliance.^14,48,49^

The need to build structural and enterprise capacity to comply, is the most consistent recommendation or priority across all sectors, as seen in Table 1.^13,34,48,50–53^^,^† Main areas of support are similar; basic infrastructure, such as providing proximate disposal points, training, facilitating access to materials and equipment, and business development, such as marketing and support with business model development and access to finance.

The need for clear mandates and standards is identified across all sectors, as seen in Table 1, alongside the financial and institutional capacity at national and local levels to actively regulate.^13,14,44,54–60^^,^† Poor resourcing of front-line officials is a common challenge and can lead to coping strategies which damage relationships with the target group.^55,61^^,^†

Recommendations for participatory approaches and bottom-up engagement, in the development and implementation of regulations, are given across all sectors as seen in Table 1.^13,44,51,53,62–64^^,^† The need to invest in close relationships and building trust between regulators and target groups is identified in street food vending, artisanal mining and faecal sludge emptying and transport, particularly for those target groups who may have previously faced harassment from the authorities.^14,51,65–67^^,^† Top down and punitive approaches are identified in analogous sectors as costly to implement, potentially weakening trust between the regulator and the target group and increasing resistance and non-compliance.^53,60,68–70^ Trust between the target group and the regulator is considered to build compliance, reflecting the perspective that trust enables regulation to safeguard public interests.^71^ ‘*Trust*’ is not always defined but appears to be consistently close to the common definition of ‘*the intention to accept vulnerability to the actions of another party, based upon the expectation that the other will perform a particular action*’.^72^ Bottom-up engagement is also required to ensure that investments into infrastructure have an impact. There were several examples in street food vending where substantial investment into fixed infrastructure, including water and sanitation services, went unused because target groups needed to be mobile to respond to customer demand.^73^

All sectors also confirmed the need to retain credible sanctions.^13,58,74,75^ Analogous sectors identify that effective sanctions are immediate and relevant to the target groups, such as vehicle impoundment.^75^ Sanctions for non-compliance need to be much higher than the cost of compliance, but excessive penalties may incentivize corruption, by allowing a greater margin for payoffs of officials.^58,76,77^ Threatening greater sanctions without a visible increase in supervision or capacity for enforcement may have a negligible or even negative impact on compliance.^68,74,75^ Measures to reduce the risk of corruption are needed alongside the development of sanctions.^76,77^

The potential for consumer or other civil society groups to take a role as a surrogate regulator is identified for alternative water providers, solid waste management, construction waste management and faecal sludge emptying and transport.^13,58,78^ ‘*Surrogate regulators*’ being ‘*third parties that are not the primary target of regulation yet contribute to the delivery of one or several regulatory functions*’.^79^ In solid waste management and faecal sludge emptying and transport public engagement is also considered as a route to address the stigma faced by the target group.^14,80^ Surrogate regulators may reduce monitoring costs in the long term, although government support would be needed to raise awareness of unsafe practices and the importance of compliance.^32,34,59^ Effective surrogate regulators require careful selection, support, oversight, appropriately defined incentives and clear frameworks for engagement.^13,79^ The full cost of this process does not seem to have been considered in the literature reviewed.

Information based regulation may have limited application in faecal sludge emptying and transport. In faecal sludge emptying and transport the use of online tracking services, such as Sanitracker^12^ was identified by key informants as one of the most promising developments in regulatory tools. However, this enthusiasm should be tempered; in informal transport, tracking target groups can provide reliable routing and service data, but are a regressive form of regulation which risks marginalizing vulnerable users, see Table 2.^81–83^ Specialist reviewers gave examples of where vehicle tracking was circumvented by target groups due to lack of ownership or high costs. Big data, modelling and tracking target groups have been successfully used in construction waste management for surveillance and targeted sanctions, but are less effective for smaller volumes of waste or in unplanned areas, as common in faecal sludge emptying and transport.^46,84,85^

### Behavioural drivers: economic, social and normative motives

Economic motives are consistently noted as the primary barrier to, and driver of, compliance across sectors as seen in Table 1.^14,25,34,44,51,86,87^^,^† A wide range of economic motives are reported, from maximising income, or growth, to a need for predictability and stability.^13,25,34,44,51,86–88^ Target groups move between seeking profit and stability dependent on their current circumstances and needs.^51,89^

Removing or reducing economic barriers, for example by removing charges for safe disposal, reducing licensing or permitting fees, and facilitating access to personal protective equipment, can improve compliance. However it may have limited impact without wider interventions to increase the social value placed on compliance, Table 2.^13,40,43,87,88^ This point is relevant learning for faecal sludge emptying and transport. Tipping charges are often used to finance operating costs of faecal sludge treatment plants but may be driving unsafe disposal.^14^ National water and sanitation regulators across eastern and southern Africa benchmark utilities against operational cost recovery which can lead to business models which prioritize efficiency over equity.^90,91^ Access to basic personal protective equipment, or other requirements for occupational health and safety, are not adequately considered in any of the sanitation financing tools considered for citywide inclusive sanitation.^91^

Analogous sectors identify that economic incentives should be structured to improve quality and safe practices, rather than incentivizing speed of service delivery. In informal transport a minimum regular salary and worker protection is found to improve safety of service, as opposed to target setting and increased precarity, see Table 2.^87^ This point is relevant learning for faecal sludge emptying and transport given that commission-based models were planned or being implemented by several key informants. These findings indicate that the design of commission models needs to include incentives for service quality and protection for workers, in addition to incentivizing scale of service provision.

In all sectors, but particularly in street food vending and solid water management, these economic motives are tempered by social and normative motives, as seen in Table 1. Individuals and organisations want to be seen to do the right thing and be perceived positively by others. Street food vendors, artisanal miners and faecal sludge emptying and transport service providers were identified as embedded social actors.^14,25,89^ In informal transport, alternative water providers and faecal sludge emptying and transport, working practices and prices charged were mediated by personal or kinship networks and normative drivers around the right to basic services.^14,27,86,92^ There are examples of target groups choosing sub-contractors, suppliers, daily transport routes, employees and employers, based on personal or kinship networks. There are multiple examples of target groups' flexibility in adjusting prices or restructuring payments dependent on households' ability to pay. These findings highlight the need to protect the vulnerable households and workers who may have benefitted from previous informal systems, as services are progressively regulated.

Status and recognition of their important role in providing basic services is an important motivator for street food vendors, alternative water providers, and waste pickers in solid waste management.^25,33,36^ Normative motives around fairness and procedural justice are evidenced in informal transport services and artisanal mining.^54,65^ Normative motives are noted as being so strong in alternative water providers, in mediating prices and access to services, that the moral economy is proposed as a basis for regulation.^66^

The needs of faecal sludge emptying and transport service providers are well documented, including occupational health and safety, legal protection and social protection schemes, dignified livelihoods, status and protection from harassment.^13,91,93,94^ A deeper understanding of social and normative drivers is identified as an important research gap for building compliance of faecal sludge emptying and transport service providers.^80,95^ Most key informants were planning to use, or were currently using, social motives to build compliance; recognizing sanitation workers as noble and raising their social status through professionalization of the service, including visible use of personal protective equipment and increased mechanization. However, research supporting the interventions were scant and in key informant interviews normative motives linked to the positive public health and environmental impacts of safe practices were rarely considered for front line providers.

### Adapting rules and regulations to make compliance feasible

The negative social and environmental impacts of criminalizing practices where there are no viable and affordable alternatives is documented across all sectors.^14,52,92,96,97^^,^† It is recognized across all sectors that regulators need to understand the technical and financial feasibility of compliance.^13,43,60,88,92^ This point is relevant for faecal sludge emptying and transport given the well documented difficulties in accessing and emptying latrine pits and septic tanks particularly in low-income areas.^14^ A review of sanitation financial tools highlighted that there was limited consideration of low-income areas.^91^ Key informants highlighted concerns about the difficulty of estimating the cost of compliance given the lack of data and the potential for the cost of compliance to push service providers underground or out of business.

All sectors, apart from construction waste management, highlight the importance of recognizing and working with the skills and structures of the target group, as seen in Table 1, including their proximity to customers, market understanding and flexibility or responsiveness.^14,45,51,73,98,99^^,^† The specialist skills of faecal sludge emptying and transport service providers were recognized by key informants, however mapping and understanding informal service providers was advanced in only a small number of locations.

Explicit decriminalization of the target group, not just tacit agreement to continue working, is given as an important step in regulating street food vendors, alternative water providers, and waste pickers in solid waste management.^100–102^ This is a relevant learning for faecal sludge emptying and transport; key informants provided multiple examples of where decriminalization of manual handling of faecal sludge at the city level had been done tacitly, without changes in the legal framework.

Regulations should be adapted to align with existing practices and technology, building from the existing knowledge base.^96,101,103^ This reflects the broader importance of building from, or aligning with, informal regulations.^104^ Across analogous sectors regulations developed for larger commercial companies needed to be adapted for individuals, smaller enterprises or less formal target groups which may have lower capacity and have been historically criminalized and marginalized. Going beyond communication and simplifying the regulations to facilitate understanding is recommended in analogous sectors.^37,43^ Requirements or standards were adapted for transport, types of equipment used, security checks, registered addresses and reporting requirements.^89,101^ This point is relevant for faecal sludge emptying and transport; key informants described how similar adaptations were needed, or being implemented. For example, standards were being adapted for transport to include provisions for push carts and barrels, permitting and reporting requirements for lower capacity providers were being waived or simplified. The adaptations described were largely tacit, agreed locally, without changes in the legal framework. One outlier is Nakuru Water and Sanitation Services Company, who went ahead of national policy to establish local by-laws adapted for onsite sanitation, as documented in the African Water and Sanitation Regulators Association (ESAWAS) technical guide to support utilities in adopting responsibility for onsite sanitation (2026). ISW^67^ also recommended simplifying bureaucracy to facilitate engagement with sanitation workers.

There were recommendations across all sectors to not sanction target groups where compliance is not feasible, where capacity to comply is very low and which are providing an essential public service where there is no alternative.^13,31,100,102,105–107^ Use of tacit amnesties are also reported as an important part of building compliance in faecal sludge emptying and transport.†

Differential regulations, and the use of amnesties, can go much further in enabling change. Sector specialists highlighted how in artisanal mining differentiated regulations allow lower capacity organisations to access support and benefits in exchange for engagement and a commitment to future improvement. For example, a miner can be ‘*formal*’ in Guyana but still use mercury. A miner in Peru can be ‘*in the process of formalizing*’ and have to comply with few actual requirements but still enjoy the same advantages of formal miners. These approaches are considered necessary in order to effectively engage with groups who have been historically marginalized and criminalized by regulatory authorities. However, in faecal sludge emptying and transport, explicit acceptance of continued poor management of faecal sludge as part of bringing service providers on board, is likely to be contentious. Key informants highlighted that standards to protect public or worker health were non-negotiable, even where these were not enforced, or indeed not enforceable, in practice.

### Understanding the informal economy

Across every analogous sector reviewed, apart from construction waste management, a similar process is reported; a heavy-handed, and often bureaucratic, approach to enforcing regulation and attempting to displace informal workers and enterprises, followed by a (slowly) growing recognition that this approach does not improve practice, and can have a significant negative social impact.^24,51,101,108–111^ Heavy handed responses range from violent government oppression for artisanal mining, to more benign neglect for the case of alternative water providers.^110,111^ Faecal sludge emptying and transport appears to be going beyond these analogous sectors in the progressive engagement and early recognition of the importance of engaging informal workers and enterprises.^13,88^^,^† For example the ESAWAS technical guide to support utilities in adopting responsibility for onsite sanitation (2026) describes how Malindi Water and Sewerage Company in Kenya has built trust with informal emptiers through a slow and iterative process using word of mouth and informal networks and addressed fears of harassment.

Differing perceptions and understanding of informal workers and enterprises are discussed in the analogous sector literature reviewed. In informal transport services and in solid waste management, the formal and informal economy are presented as interdependent, with the informal workers and enterprises typically being subordinate, providing services and keeping costs lower for formal enterprises.^112^ For street food vendors and artisanal mining, the informal economy is typically presented as intrepid entrepreneurs working around burdensome bureaucracy and a hostile legal system.^113^ The informal economy may have been engaged with the formal economy for many years and have established ways of working.^27,97,114^ The formal and informal economy may be intertwined, with formal services being dependent on informal workers and enterprises.^86,97^ This is relevant learning for faecal sludge emptying and transport, where the formal and informal economy appear to be perceived as binary and separate; and the recommended policy response is that governments should provide support to transition the informal workers and enterprises to becoming formal.^13,115^

Research in street food vendors, artisanal mining, alternative water providers, and waste pickers in solid waste management identifies how formalization risks entrenching existing power inequalities and further marginalizing informal groups.^114,116,117^ ‘*Formalization*’ follows the ILO definition of bringing ‘*informal*’ service providers ‘*under the regulation of the state with the advantages and obligations that this entails*’,^118^ The extensive literature in artisanal mining outlines how formalization should be seen as a political process, rather than a technical product.^29,30^ Forthcoming publications in faecal sludge emptying and transport argue for greater local government support to avoid the unintended outcomes of formalization.^119^ However, the risks of entrenching inequalities and the political nature of the process appear to be relevant learning for faecal sludge emptying and transport; in the key informant interviews, and the literature, formalization was largely presented as linear and technical.^13^

The need for policies which incorporate informal workers and enterprises is identified across all analogous sectors, as seen in Table 1.^37,44,57–60^ Accepting a certain level of informality, while building incremental improvements in practice is a pragmatic approach recommended by some academics.^28,53^ Incorporating, rather than formalizing, the informal sector means enabling informal workers to have rights to the same level of social protections as are enjoyed by formal workers, and being allowed to organize and to have a representative voice in rule-setting and policymaking processes.^36,115,120^ Organisational literature on faecal sludge emptying and transport service providers reflects this approach, recommending government interventions to improve occupational health and safety for all workers, regardless of employment status.^121^ This approach requires political willingness to explicitly allow informal workers to continue working without following the same work practices as formal enterprises.^120^

Additional resources are required to regulate informal workers and enterprises because they are likely to be dispersed, diverse, and may require regulators to engage through new networks and use new approaches.^14,81,84,88^^,^† External support, from international financing institutions or NGOs, may be required through the transition period, as shown by the successful examples of regulating alternative water providers.^122^

Alongside the need for more inclusive policies, and associated resourcing, the analogous sector review identifies the need to rethink the definitions and use of ‘*informal*’. The term ‘*informal*’ is questioned by groups working across street food vendors and solid waste management. It risks homogenizing huge and heterogenous groups and it is difficult define without inferring the superiority of the formal sector.^123^ The use of ‘*informal*’ is recognized to be useful for policy and for understanding the role and responsibility of government, however it is also politically loaded and problematic, with a history of being used selectively to marginalize target groups.^115^ ‘*Informal*’ is rarely used to describe wealthy or politically connected target groups who are circumventing rules and regulations.^115^

Alongside the points above, specialists in both informal transport services and alternative water providers highlighted that some literature can be naive in its perception of informal, off-grid or alternative providers. Some enterprises may be actively criminal and obstruct government investment. These providers may be an important part of the transition towards universal access, however seeing them as the end goal risks accepting lower quality services for lower income areas. Public investment into high-capacity formal service providers is still needed.

### Engaging the informal sector: membership based organisations

Two main approaches for working with the informal sector are presented across analogous sectors; contracting through the formal sector or working with Membership based organisations.

Larger formal organisations contracting smaller or informal target groups are discussed in street food vendors, artisanal mining, and waste pickers in solid waste management.^28,51,116^ This approach is identified as typically non-participatory and transactional, and there are examples of where it has led to the exploitation of smaller or informal groups.^51,116,124^ If this approach is taken, then contracting arrangements need to be set up so that both groups benefit from working together, and the larger organisation has a vested interest in protecting the smaller.^51,125^ This point is relevant for faecal sludge emptying and transport; key informants highlighted how manual pit emptiers may be engaged by vacuum tanker operators for difficult to access or less stable pits, or to empty compacted sludge cake.

Membership-based organisations, including associations, unions, cooperatives, or less formal groupings are referenced across all analogous sectors as having a positive impact on compliance.^33,56,126–131^ In solid waste management there is evidence that membership-based organisations can support better working conditions, recognition and reduction of stigma.^51,120^ Working through membership-based organisations has the potential to improve visibility, build relationships with the state, build professionalism and capacity of the sector, support the implementation of minimum standards and enable collective incentives and sanctions.^33,37,128,132–134^

Successful structures of membership-based organisations are highly context specific.^36^ However, in analogous sectors there was a consistent recommendation that membership-based organisations need to build from existing networks and norms, they should be developed from the bottom-up.^33,56,126,127–131^ At the same time long-term institutional support is essential; membership-based organisations need ongoing support from local and national government. Common recommendations include ensuring a reliable income for services provided, access to equipment and materials, support to develop internal processes, and collective access to social support services including occupational health and safety improvements.^36,126,133^

Membership-based organisations are more likely to be successful if the group itself has capacity to make decisions and enact change, if it has strong and equitable leadership, if it has visibility and voice – for example if the organisation is in a larger city, or if it has closer proximity to power.^134,135^ Membership should be voluntary and based on perceived benefits; enforced membership and payment of fees has been identified as regressive and stoking resistance.^58^ National, regional and international groupings of membership-based organisations have had a positive impact on development, increasing the visibility and ability to influence policy.^120^

Membership-based organisations may be particularly important for vulnerable groups, including gender-based vulnerability, and provide a route for greater influence. However, the equitable distribution of costs and benefits of compliance is highly dependent on leadership and internal capacity. The leaders of membership-based organisations are influential in the distribution of costs and benefits of group compliance; leadership and accountability structures are important for equity.^133^ As given in Table 2, oversight and support is needed from regulatory bodies to ensure that regulation through membership-based organisations does not lead to the exploitation of the more vulnerable.^133,136^

The successes and challenges in analogous sectors developing and regulating through membership-based organisations appear to be a potential area of learning for faecal sludge emptying and transport. WHO^137^ includes the role of associations in capacity building in the top 10 research questions for the sector. Mremi *et al.*^92^ and Tomoi *et al.*^80^ describe how membership-based organisations could improve working conditions for emptiers. There is scant peer-reviewed literature around experience with membership-based organisations in faecal sludge emptying and transport, despite the growth of pit-emptying associations across Africa under the umbrella organisation of the Pan-African Association of Sanitation Actors, formed in 2019. Organisational literature from the Initiative for Sanitation Workers describes how bottom-up support to collective action has enabled sanitation workers to form organisations, gain government recognition and advocate for change.^67,138^ Several key informants identified membership-based organisations as the most feasible approach to engaging the multiplicity of private service providers and reported their planned or ongoing engagement.

### Segregating service providers

Based on the findings in this article we propose to adapt regulatory approaches dependent on the service providers' capacity and willingness to comply. Service providers are segregated by capacity reflecting the prevalence of low-capacity providers, and the difficulty of compliance. Willingness to comply, or attitude to compliance, is an established approach for disaggregating target groups to determine the likelihood of compliance and is a useful proxy when actual behaviour is difficult to observe.^7^

Adapting regulatory strategies to the intent and ability to comply reflects the principles of responsive regulation.^8^ Categorizing service providers into groups and designing discrete regulatory strategies for each is considered simpler than implementing the pyramid of responsive regulation, while still allowing the flexibility for service providers to move between groups. This approach responds to previous critiques of responsive regulation as being complex and not being adapted to low resource settings.^146^

In practice, determining capacity and willingness to comply would rely on developing an agreed process, where professional judgements against established levels of competency and attitude, are supported by appropriate scrutiny. The decisions made, together with the reasoning, should be made publicly available to ensure full transparency in the overall process.

Determining capacity to comply is expected to rely upon a technical and financial assessment. However, determining willingness to comply is more subjective process. The examples from artisanal mining and solid waste management, where willingness to comply is, at least initially, effectively determined from attendance at meetings, trainings and stated intent may be perceived as high risk. There is always the potential for corruption – for example, payment in return for recognition as ‘willing to comply’; one of the reasons why normative motives are so important. Designing for the implementation of this framework warrants further consideration.

### Limitations and generalizability

#### Literature profile and validity of the methods used

The vast majority of the literature reviewed is descriptive, based on literature review, policy analysis and case studies (91%). There are only 19 papers which purport to give causal explanations, the vast majority are examples of approaches which have been positively received or proposals to address current barriers to compliance. The lack of causal links in analogous sectors limits the confidence of recommendations which can be made.

The published research in different sectors is dominated by different geographies. Research on waste pickers in solid waste management is largely centred in Latin America; fly tipping in construction waste management in east and south-east Asia; street food vendors, informal transport service providers, artisanal mining and alternative water providers in sub-Saharan Africa as illustrated in Fig. 4. The relevance of the application of ideas across geographies, cultures and wealth brackets is a further cause for caution.

The use of online mapping tools in literature searches substantially widened the scope of the review. The two literature mapping tools identified up to 14 additional relevant papers for each sector. In total, they made up 30% of papers incorporated into the final coding and synthesis. The specialist review also added substantially to the depth of literature considered. Specialist reviews identified up to 15 additional relevant papers for each sector, making up 24% of the final set of literature considered. Additional tacit knowledge, around bias within the published literature and observations from experience which are not reflected in literature, from specialist reviewers have added greater depth to the syntheses. These steps have therefore been important in making the literature review more robust. However, there were ultimately only a limited number of papers reviewed and shortlisted through this process, potentially leading to a lack of depth. The rapid review has also led to a much greater reliance on recent papers as the coding process started with recent literature reviews and worked forwards.

The overall validity of the exercise in being able to identify and synthesize current thinking in building compliance appeared to be supported by reviewer feedback. Much of the sector specialist feedback reinforced the points raised through the review. All reviewers added aspects to be considered and additional papers. There were two examples where an overall recommendation taken from the literature was challenged and subsequently amended. The first was to clarify that alternative water providers may not be a sustainable long-term alternative to utility provision. The second was to correct that there were no examples of successful formalization in artisanal mining and to underline the importance of adapting policies to the capacity of target groups. One reviewer identified a much larger body of literature which could have been considered, using a wider set of key words and additional database searches, but also concluded that these additional searches would add depth, but would not fundamentally change the conclusions taken from the rapid review.

The application of findings to inform other sectors needs to be done with caution. Several specialist reviewers queried whether there could be parallels drawn between their sector and faecal sludge emptying and transport, citing the unique nature of the challenges faced. Several also highlighted that all regulatory responses needed to be highly context specific to be effective. The rapid review was noted to flatten out much of the difference in implementation across locations and groups of service providers or enterprises. The proposed recommendations derived through this process would need validation by practitioners working directly on these challenges before being considered for further research and implementation.

This article does not claim to be definitive and there may be important developments in the design and implementation of regulation which are not covered. One such area is the growing application of behavioural insights to public governance.^139^ The scope and application of this learning should be an area of future research for faecal sludge emptying and transport given the potential for relevant insights around addressing corruption, the application of differential regulations, and the integration of behavioural public policy and responsive regulation.^140–142^

#### Reflections on methods to generate potential good practice

This article adds to existing methods papers in illustrating the benefits of artificial intelligence as a research tool.^143^ Applying online tools which use citation mapping and artificial intelligence enabled consistently sourcing of relevant literature across sectors outside of their expertise and synthesize evidence from analogous sectors. Incorporating tacit knowledge through specialist review was essential to check understanding and identify gaps.

This approach was taken due to the lack of evidence from within the urban sanitation sector of successful regulatory approaches. One of the most significant benefits has been adding weight to approaches already being taken by leading national regulators and mandated service authorities, particularly those approaches which may have lower levels of political support. For example, the analogous sector review reinforces that the use of amnesties and engagement of the informal economy is positive and progressive. The analogous sector review has also provided an early warning of potential issues. For example, the risks of exploitation when working through membership-based organisations and how vehicle tracking could be a regressive form of regulation.

Possibly the most interesting insight from the methods used is how deeply entrenched the authors and the specialist reviewers are in our respective sectors. We entered this discussion unsure that the process would be useful and confident that road-transported sanitation^19^ is quite unique in its transdisciplinary nature and level of complexity. Similarly, a number of specialist reviewers highlighted that it would be difficult to identify relevant learning because their sector is quite unique. However, there is a high level of consistency in the findings across these apparently disparate sectors. Where there were substantial differences, they often illuminated an interesting learning. For example, in artisanal mining, policy recommendations could only be made knowing the size and capacity of the organisation. Different understanding of formality across the sectors informs different perspectives which could be taken in faecal sludge emptying and transport.

The response of this group of individuals, who were open-minded enough to engage fully in this process, reflects both a necessary caution of transferring ideas between sectors, but also potentially the difficulty we all face in getting out of our silos.

## Conclusions

The cross-sector review reinforces a number of good practices in building compliance of faecal sludge emptying and transport service providers, including the principles of the adapted holistic model of business compliance and the steps outlined in the WHO roadmap for sanitation regulation.^13,14^

The overall approach given in Fig. 5 is responsive regulation, which responds to the environment and the conduct and capacity of the individual or enterprise, starting with developing relationships and capacity-building. It incorporates elements from across the spectrum of regulatory strategies and instruments, from the importance of established standards and retention of criminal sanctions for command-and-control regulation, to co-regulation, support mechanisms and capacity building.^144–146^

This paper makes an methodological contribution through the analogous sector synthesis, assisted by online literature mapping; demonstrating how useful it can be to step out of sectoral silos. The further contribution of this paper lies in bringing a more substantial evidence base around what has worked, or not, in building compliance into the urban sanitation sector. This contribution is primarily in the four areas where the review identified important potential learning from analogous sectors; behavioural drivers, adapting rules and regulations to make compliance feasible, understanding the informal economy, and developing and regulating through membership-based organisations, which are discussed below. However, this contribution is also in the identification of the specific insights given in Table 2, some of which do not link to these four areas but appear to resonate with feacal sludge emptying and transport service provision. Prominent among these insights are that expectations of online service booking and tracking services may need to be tempered, given the limitations and the negative impact of similar applications on vulnerable groups evidenced in other sectors. However, satellite imagery could facilitate remote monitoring and identification of unsafe disposal points, at least in planned settlements where street layouts are predictable.

Economic motives are important; removing economic barriers and demonstrating incentives, which align with the target groups' priorities of profit or stability, are an important part of any response. As a minimum, mandated service authorities should remove tipping fees for safe disposal of faecal sludge and invest in proximate disposal points for lower-capacity groups. This change may require a critical review of the expectations of cost-recovery in the sector and would provide a clear signal of the importance of compliance. However, regulatory approaches should not assume purely economic actors, beyond meeting livelihood requirements. Social and normative drivers influence both how services are provided currently and responses to regulation. Regulators should invest in greater understanding and use of social and normative drivers, both for service providers and for the wider public and policy makers. The important role of faecal sludge emptying and transport service providers in protecting public health and the environment needs to be widely recognized and celebrated.

Existing tacit adaptations to rules and regulations to make compliance understood and feasible should be made explicit and widespread. These adaptations include amending regulations to make them easier to understand, decriminalization of manual handling, adaptation of standards to be appropriate to manual emptying, simplification of permitting, administrative and reporting requirements. Many of these adaptations are already made across eastern and southern Africa and are not considered contentious. However, even with these adaptations, providing safe services will remain challenging for many low-capacity service providers. Mandated service authorities need to be able to engage positively with non-compliant service providers to start to change practices. However, recognizing and rewarding incremental improvements may be challenging given the public health and environmental risk from faecal sludge. A less contentious approach of ‘*constrained discretion*’^147^ may be useful for faecal sludge emptying and transport. This approach would mean that differentiated regulations and support to non-compliant service providers would be trialled within geographic and time boundaries; changes could be piloted by individual mandated service authorities with the agreement and support of the national regulator.

Emerging good practice in road-based sanitation considers progressive formalization as the preferred way forward. However, considering analogous sectors gives a much wider range of understanding of the formal and informal sectors as intertwined and interdependent, and as informality as being a pragmatic choice. This different perspective indicates that ‘*informality may not be the problem, and that formalization may not be the answer*’.^123^ Segregating service providers by capacity and willingness to comply, rather than level of formality, as shown in Fig. 5, would facilitate inclusive and appropriate regulatory responses which avoid labelling service providers as ‘*informal*’ while enabling those potentially criminal service providers to be targeted.^148^

Membership based organisations are identified as likely to be a more positive route to engaging the informal sector than subcontracting through formal enterprises. Membership-based organisations are expected to be become a more important part of faecal sludge emptying and transport regulation going forward. This co-regulation needs to be developed from the bottom up and from existing norms and networks, however it also needs consistent support from regulators to build capacity, visibility and ensure that vulnerable members are not exploited or marginalized. These recommendations may sound generic; the main message is that regulating through membership-based organisations is resource intensive. Regulators have a responsibility to understand and support the internal workings of membership-based organisations, it is not a hands-off approach.

Scaling safe services through a regulated private sector has been seen as the only immediately feasible option, given significant funding gaps.^5^ However, this option still requires substantial public investment, to ensure that low-capacity service providers and membership-based organisations continue to improve and bring service quality up instead of levelling down to the lowest common denominator. As services are progressively regulated, regulators also need to protect the vulnerable groups who may have benefitted from previous informal systems. Adapting rules and regulations to groups of service provider based upon capacity and attitude to compliance is proposed to ensure that support, incentives, amnesties and sanctions are targeted and build compliance, rewarding those attempting but failing to comply, and only sanctioning those who are wilfully non-compliant. A punitive approach is likely to push many essential service providers either underground or out of business. Potential good practice for working with different groups of service providers are summarized on Fig. 5, including ways of working with membership-based organisations. These recommendations would need to be validated with practitioners before being considered as a potential base for designing action research around building compliance in faecal sludge emptying and transport service providers.

## Author contributions

CG led the conceptualization, methodology, investigation, data curation, formal analysis, administration, and writing the original draft. AP and PL contributed to the conceptualization and supervised the research. AP was responsible for funding acquisition. AO, SM and FM contributed to the investigation by providing substantive feedback on the sector summaries. AP, PL, AO, SM and FM validated the research findings and reviewed the draft manuscript.

## Conflicts of interest

There are no conflicts to declare.

## Supplementary Material

EW-012-D5EW01108F-s001

## Data Availability

The data supporting this article have been included as part of the supplementary information (SI). Additional data for this article, including anonymised feedback from specialist reviewers are available at Cranfield University Collection of E-Research (CERES) Research Repository at https://doi.org/10.57996/cran.ceres-2798. Supplementary information is available. See DOI: https://doi.org/10.1039/d5ew01108f.

## References

[cit1] JMP , Progress on household drinking water, sanitation and hygiene 2000–2024: special focus on inequalities, United Nations Children's Fund and World Health Organization, 2025

[cit2] Peal A., Evans B., Ahilan S., Ban R., Blackett I., Hawkins P., Schoebitz L., Scott R., Sleigh A., Strande L., Veses O. (2020). Estimating safely managed sanitation in urban areas; lessons learned from a global implementation of excreta-flow diagrams. Front. Environ. Sci..

[cit3] Wolf J., Hubbard S., Brauer M., Ambelu A., Arnold B. F., Bain R., Bauza V., Brown J., Caruso B. A., Clasen T., Colford J. M., Freeman M. C., Gordon B., Johnston R. B., Mertens A., Prüss-Ustün A., Ross I., Stanaway J., Zhao J. T., Cumming O., Boisson S. (2022). Effectiveness of interventions to improve drinking water, sanitation, and handwashing with soap on risk of diarrhoeal disease in children in low-income and middle-income settings: a systematic review and meta-analysis. Lancet.

[cit4] ESAWAS , The Water Supply and Sanitation Regulatory Landscape Across Africa—Continent Wide Synthesis Report, Eastern and Southern Africa Water and Sanitation Regulators Association, Lusaka, 2022

[cit5] Schelbert V. and Narayan A., Discussion Paper Citywide Inclusive Sanitation: Reviewing the State of the Art, International Water Association, 2024, 10.2166/9781789064964

[cit6] Black J. (2002). Critical reflections on regulation. Australas. J. Leg. Philos..

[cit7] Braithwaite V. , Dancing with tax authorities: motivational postures and non-compliant actions, Taxing Democracy, 2003, vol. 3, pp. 15–39, 10.4324/9781315241746

[cit8] Baldwin R., Black J. (2008). Really responsive regulation. Mod. Law Rev..

[cit9] Wilcox J., Rutayisire B., Kuria N., Evans B., Bartram J., Sklar R. (2023). Developing formal pit-latrine emptying businesses for hard-to-serve customers: resources, methods, and pricing structures. J. Water, Sanit. Hyg. Dev..

[cit10] IWA , Lessons learnt regulating for citywide inclusive sanitation, International Water Association, 2022

[cit11] Lerebours A., Scott R., Sansom K., Kayaga S. (2022). Barriers and enablers to the regulation of sanitation services: a framework for emptying and transport services in Sub-Saharan African cities. Front. Environ. Sci..

[cit12] ESAWAS , Citywide Inclusive Sanitation (CWIS) Regulatory Journeys in Six Countries, Eastern and Southern Africa Water and Sanitation Regulators Association, Lusaka, 2024

[cit13] WHO , Regulatory roadmap to safely managed sanitation, World Health Organization, Geneva, 2025

[cit14] Grisaffi C., Leinster P., Mugo K., Drabble S., Parker A. (2025). Safe faecal sludge emptying and transport: compliance challenges and models for a public good. H2Open J..

[cit15] Fernie S., Green S. D., Weller S. J., Newcombe R. (2003). Knowledge sharing: context, confusion and controversy. Int. J. Proj. Manage..

[cit16] Pulido-Gómez S., de Jong J., Rivkin J. W. (2025). Cross-sector collaboration in cities: learning journey or blame game?. J. Public Adm. Res. Theory..

[cit17] Lah O. (2025). Breaking the silos: integrated approaches to foster sustainable development and climate action. Sustain. Earth Rev..

[cit18] Willetts J. , Winterford K., Dickin S., Soeters S., Uytewaal E., Butterworth J. A. and Rozenberg E., Strengthening mutual accountability in partnerships for WASH: Part 1-Literature review and learning from other sectors, UTS Institute for Sustainable Futures, SEI, IRC and SWA Research and Learning Constituency, 2021

[cit19] Grisaffi C., Leinster P., Sipuma R., Owako E., Parker A. (2026). New definitions for good practice: Regulators as activists for urban road-transported sanitation in eastern and southern Africa. PLOS Water.

[cit20] Hamel C., Michaud A., Thuku M., Skidmore B., Stevens A., Nussbaumer-Streit B., Garritty C. (2021). Defining rapid reviews: a systematic scoping review and thematic analysis of definitions and defining characteristics of rapid reviews. J. Clin. Epidemiol..

[cit21] Falagas M. E., Pitsouni E. I., Malietzis G. A., Pappas G. (2008). Comparison of PubMed, Scopus, web of science, and Google scholar: strengths and weaknesses. FASEB J..

[cit22] Weishuhn M. , Inciteful: Citation network exploration, 2025, Available at: https://inciteful.xyz, (accessed 10 September 2025)

[cit23] Research Rabbit , Reimagine Research, n.d., https://www.researchrabbit.ai/

[cit24] Pulliat G., Block D., Bruckert M., Nussbaum-Barberena L., Dreysse C., Dupé P., Perrin C. (2023). Governing the nurturing city: the uneven enforcement of street food vending regulations. Urban Geogr..

[cit25] Adaku A. A., Egyir I. S., Gadegbeku C., Kunadu A. P. H., Amanor-Boadu V., Laar A. (2024). Barriers to ensuring and sustaining street food safety in a developing economy. Heliyon.

[cit26] FAO , Promises and Challenges of the Informal Food Sector in Developing Countries, Food and Agricultural Organization, Rome, 2007

[cit27] Rekhviashvili L., Kębłowski W., Sopranzetti C., Schwanen T. (2022). Informalities in urban transport: Mobilities at the heart of contestations over (in) formalisation processes. Geoforum.

[cit28] Sauerwein T. (2023). Should mining companies partner with artisanal miners? Why only time will tell. J. Rural Stud..

[cit29] Siegel S., Veiga M. M. (2009). Artisanal and small-scale mining as an extralegal economy: De Soto and the redefinition of “formalization”. Resour. Policy.

[cit30] Ofori A. D., Mdee A., Van Alstine J. (2021). Politics on display: The realities of artisanal mining formalisation in Ghana. Extr. Ind. Soc..

[cit31] Veiga M. M., Fadina O. (2020). A review of the failed attempts to curb mercury use at artisanal gold mines and a proposed solution. Extr. Ind. Soc..

[cit32] Gerlach E. , Regulating Alternative Providers for the Poor, in Regulating water and sanitation for the poor: Economic regulation for public and private partnerships, ed. R. Franceys and E. Gerlach, Routledge, 2008, pp. 195–209, 10.4324/9781849772310

[cit33] Wutich A., Beresford M., Carvajal C. (2016). Can informal water vendors deliver on the promise of a human right to water? Results from Cochabamba, Bolivia. World Dev..

[cit34] Du L., Xu H., Zuo J. (2021). Status quo of illegal dumping research: Way forward. J. Environ. Manage..

[cit35] Ramos M., Martinho G. (2023). An assessment of the illegal dumping of construction and demolition waste. Clean. Waste Syst..

[cit36] Kain J. H., Zapata P., de Azevedo A. M. M., Carenzo S., Charles G., Gutberlet J., Oloko M., Pérez Reynosa J., Campos M. J. Z. (2022). Characteristics, challenges and innovations of waste picker organizations: A comparative perspective between Latin American and East African countries. PLoS One.

[cit37] Alba R., Bruns A., Bartels L. E., Kooy M. (2019). Water brokers: Exploring urban water governance through the practices of tanker water supply in Accra. Water.

[cit38] Oladipo-Adekeye O. T., Tabit F. T. (2021). The food safety knowledge of street food vendors and the sanitary compliance of their vending facilities, Johannesburg, South Africa. J. Food Saf..

[cit39] Ibelli-Bianco C., Guimarães J. P. S., Yamane L. H., Siman R. R. (2022). Education and training: Key solution to self-management and economic sustainability of waste pickers organisations. Waste Manage. Res..

[cit40] Ramos M., Martinho G., Vasconcelos L., Ferreira F. (2023). Local scale dynamics to promote the sustainable management of construction and demolition waste. Resour. Conserv. Recycl. Adv..

[cit41] Ntunja A., Baloyi W. T. H., Teare J., Opeoluwa O., Melariri P. (2025). Impact of Educational Intervention on Hygiene Knowledge and Practices of Sanitation Workers Globally: A Systematic Review. Scientifica.

[cit42] Velez M. A., Rueda X., Henao J. P., Monroy D., Tobin D., Maldonado J., Pfaff A. (2025). Small-scale gold miners' preferences on formalization: First steps toward sustainable supply chains in Colombia. World Dev..

[cit43] Haque I. T., Kohda Y. (2020). Understanding the impact of social determinants of health in street food safety: a qualitative study in Bangladesh. Int. J. Health Promot. Educ..

[cit44] Arthur-Holmes F., Ofosu G. (2024). Rethinking state-led formalisation of artisanal and small-scale mining (ASM): Towards mining licence categorisation, women empowerment and environmental sustainability. Resour. Policy.

[cit45] Gero A., Carrard N., Murta J., Willetts J. (2014). Private and social enterprise roles in water, sanitation and hygiene for the poor: a systematic review. J. Water, Sanit. Hyg. Dev..

[cit46] Lu W. (2019). Big data analytics to identify illegal construction waste dumping: A Hong Kong study. Resources, conservation and recycling. Resour., Conserv. Recycl..

[cit47] Mateo-Babiano I., Recio R. B., Ashmore D. P., Guillen M. D., Gaspay S. M. (2020). Formalising the jeepney industry in the Philippines – A confirmatory thematic analysis of key transitionary issues. Res. Transp. Econ..

[cit48] Martinez G., Smith N. M., Malone A. (2023). “I am formal, what comes next?”: A proposed framework for achieving sustainable artisanal and small-scale mining formalization in Peru. Extr. Ind. Soc..

[cit49] Nitin K., Puja D., Sanjay C., Sunil T., Poonam K., Poojan M., Sushruti K. (2025). A Cross-Sectional Study to Assess Street Food Vendors' Adherence to the Bureau of Indian Standards (BIS) in an Urban Slum of Pune, India. Cureus.

[cit50] Khairuzzaman M. D., Chowdhury F. M., Zaman S., Al Mamun A., Bari M. L. (2014). Food safety challenges towards safe, healthy, and nutritious street foods in Bangladesh. Int. J. Food Sci..

[cit51] Aparcana S. (2017). Approaches to formalization of the informal waste sector into municipal solid waste management systems in low- and middle-income countries: Review of barriers and success factors. Waste Manage..

[cit52] Boakye-Ansah A. S., Schwartz K., Zwarteveen M. (2019). From rowdy cartels to organized ones? The transfer of power in urban water supply in Kenya. Eur. J. Dev. Res..

[cit53] Gatarin G. R. (2024). Modernising the ‘king of the road’: Pathways for just transitions for the
Filipino jeepney. Urban Governance.

[cit54] McCormick D. , Institutional development in public transport: implications of selective compliance for Nairobi's paratransit system, Institute for Development Studies, University of Nairobi, 2014

[cit55] Forkuor J. B., Samuelsen H., Yeboah E. H., Rheinländer T., Akuoko K. O. (2017). The regulation of street foods: Experiences of front-line regulators in Ghana. Urban Forum.

[cit56] Dakyaga F., Kyessi A. G., Msami J. M. (2018). Water access today and tomorrow: Domestic water sustainability under informal water supply markets in Dar es Salaam, Tanzania. J. Sustainable Dev..

[cit57] Grace D. (2015). Food safety in low- and middle-income countries. Int. J. Environ. Res. Public Health.

[cit58] Muheirwe F., Kombe W., Kihila J. M. (2022). The paradox of solid waste management: A regulatory discourse from Sub-Saharan Africa. Habitat Int..

[cit59] Swarna Swetha K., Tezeswi T. P., Siva Kumar M. V. N. (2022). Implementing construction waste management in India: An extended theory of planned behaviour approach. Environ. Technol. Innovation.

[cit60] Ardila-Gomez A., Darido G. (2025). Public Transport Reform in Developing Countries: Lessons from Experience. Transp. Res. Procedia.

[cit61] Matsinhe N. P., Juízo D., Macheve B., dos Santos C. (2008). Regulation of formal and informal water service providers in peri-urban areas of Maputo, Mozambique. Phys. Chem. Earth.

[cit62] Porter M. E., Linde C. V. D. (1995). Toward a new conception of the environment-competitiveness relationship. J. Econ. Perspect..

[cit63] Martínez L., Young G. (2022). Street vending, vulnerability and exclusion during the COVID-19 pandemic: the case of Cali, Colombia. Environ. Urban..

[cit64] Wutich A., Thomson P., Jepson W., Stoler J., Cooperman A. D., Doss-Gollin J., Jantrania A., Mayer A., Nelson-Nuñez J., Walker W. S., Westerhoff P. (2023). MAD water: Integrating modular, adaptive, and decentralized approaches for water security in the climate change era. Wiley Interdiscip. Rev.:Water.

[cit65] Heemskerk M., Duijves C., Pinas M. (2015). Interpersonal and institutional distrust as disabling factors in natural resources management: small-scale gold miners and the government in Suriname. Soc. Nat. Resour..

[cit66] Beresford M., Brewis A., Choudhary N., Drew G., Garcia N. E., Garrick D., Hossain J. M., Lopez E., Nébié E. I., Pacheco-Vega R., Roque A., Wutich A. (2024). Justice and moral economies in “Modular, Adaptive, and Decentralized” (MAD) water systems. Water Secur..

[cit67] ISW , Supporting Sanitation Workers: Lessons from the Empowerment Support Fund, Initiative for Sanitation Workers. WaterAid, 2025

[cit68] D'Amato A., Mazzanti M., Nicolli F., Zoli M. (2018). Illegal waste disposal: enforcement actions and decentralized environmental policy. Socio-Econ. Plan. Sci..

[cit69] Zozmann H., Morgan A., Klassert C., Klauer B., Gawel E. (2022). Can tanker water services contribute to sustainable access to water? A systematic review of case studies in urban areas. Sustainability.

[cit70] Ayambire R. A., Nunbogu A. M., Cobbinah P. B., Kansanga M. M., Pittman J., Dogoli M. A. (2024). Constructing alternative interpretation: Embeddedness of illegality in small-scale mining. Extr. Ind. Soc..

[cit71] Verhoest K. , Redert B., Maggetti M., Levi-Faur D. and Jordana J., Trust and regulation, in Handbook on trust in public governance, ed. F. Six, J. A. Hamm, D. Latusek, E. Van Zimmeren and K. Verhoest, Edward Elgar, 2025, pp. 360–380, 10.4337/9781802201406.00030

[cit72] Six F. and Verhoest K., Trust in regulatory regimes: scoping the field, in Trust in Regulatory Regimes, ed. F. Six and K. Verhoest, Edward Elgar, 2017, pp. 1–36, 10.4337/9781785365577.00005

[cit73] Huang G., Xue D., Wang Y. (2019). Governmentality and spatial strategies: Towards formalization of street vendors in Guangzhou, China. Int. J. Urban Reg. Res..

[cit74] Dwumfour-Asare B. (2015). Effect of local authorities' field monitoring visits on awareness of regulation and hygiene practices among street food vendors: the case of two district capitals in Ghana. J. Behav. Health.

[cit75] Seror N., Portnov B. A. (2020). Estimating the effectiveness of different environmental law enforcement policies on illegal CandD waste dumping in Israel. Waste Manage..

[cit76] Sohail M., Maunder D. A. C., Cavill S. (2006). Effective regulation for sustainable public transport in developing countries. Transp. Policy.

[cit77] Abatemarco A., Cascavilla A., Dell'Anno R., Morone A. (2024). Maximal fines and corruption: An experimental study on illegal waste disposal. Waste Manage..

[cit78] Pakizer K., Lieberherr E. (2018). Alternative governance arrangements for modular water infrastructure: An exploratory review. Compet. Regul. Netw. Ind..

[cit79] Etienne J. , McEntaggart K., Chirico S. and Schnyder G., Comparative Analysis of Regulatory Regimes in Global Economies, BEIS Research Paper Number 19, Department for Business, Energy and Industrial Strategy, UK Government, London, 2018

[cit80] Tomoi H., MacLeod C., Moriyasu T., Simiyu S., Ross I., Cumming O., Braun L. (2024). Determinants of willingness to pay for fecal sludge management services and knowledge gaps: a scoping review. Environ. Sci. Technol..

[cit81] Mapunda D. W., Chen S. S., Yu C. (2018). The role of informal small-scale water supply system in resolving drinking water shortages in peri-urban Dar Es Salaam, Tanzania. Appl. Geogr..

[cit82] Doherty J. (2022). Motorcycle taxis, personhood, and the moral landscape of mobility. Geoforum.

[cit83] Kuttler T. (2024). Urban mobilities in Mumbai: Towards worker-centric platformisation beyond ‘urban solutionism’. Urban Stud..

[cit84] Marshall S., Taylor K., Connor T., Haines F., Tödt S. (2022). Will Business and Human Rights regulation help Rajasthan's bonded labourers who mine sandstone?. J. Ind. Relat..

[cit85] Fang B., Yu J., Chen Z., Osman A. I., Farghali M., Ihara I., Hamza E. H., Rooney D. W., Yap P. S. (2023). Artificial intelligence for waste management in smart cities: a review. Environ. Chem. Lett..

[cit86] Alba R., Kooy M., Bruns A. (2022). Conflicts, cooperation and experimentation: Analysing the politics of urban water through Accra's heterogeneous water supply infrastructure. Environ. Plan. E: Nat. Space.

[cit87] Behrens R., Zuidgeest M., Durant T. (2025). Relationships between paratransit passenger satisfaction and driver labour conditions in Sub-Saharan Africa. Afr. Transp.
Stud..

[cit88] Wankhade K. , Vijendra M. and Yaseen S., Addressing the missing middle in the sanitation chain: Improving emptying and transport services in Tamil Nadu, India, VeriXiv, preprint, vol. 2, p. 178, 10.12688/verixiv.1575.1

[cit89] Jiménez A. D., Smith N. M., Holley E. A. (2024). Towards sustainable ASM-based livelihoods: The role of institutional arrangements in the formalization of artisanal and small-scale mining. Resour. Policy.

[cit90] ESAWAS , Multi-country WSS Utility Benchmarking Report 2023/24, Eastern and Southern Africa Water and Sanitation Regulators Association, Lusaka, 2025

[cit91] Franco C. S., Singh S., Brdjanovic D. (2025). Sanitation financial tools (SFTs) for citywide inclusive sanitation (CWIS): A critical review. J. Environ. Manage..

[cit92] Mremi A., Kimwaga R., Mulungu D. M., Izdori F. (2025). Managing sanitation in unplanned urban areas: insights into manual emptying of onsite systems in Tandale, Dar es Salaam. J. Water, Sanit. Hyg. Dev..

[cit93] Tolera S. T., Temesgen S., Mulat Endalew S., Alamirew T. S., Temesgen L. M. (2023). Global systematic review of occupational health and safety outcomes among sanitation and hygiene workers. Front. Public Health.

[cit94] Berihun G., Desye B., Berhanu L., Daba C., Walle Z., Geto A. K. (2025). Prevalence of respiratory symptoms and associated factors among sanitation workers in Sub Saharan Africa: a systematic review and meta-analysis. Front. Public Health.

[cit95] Bhakta A., Cawood S., Zaqout M., Evans B. (2022). Sanitation work: Realizing equity and inclusion in WASH. Front. Water.

[cit96] Spiegel S., Keane S., Metcalf S., Veiga M. (2015). Implications of the Minamata Convention on Mercury for informal gold mining in Sub-Saharan Africa: from global policy debates to grassroots implementation?. Environ. Dev. Sustain..

[cit97] Muheirwe F., Kombe W. J., Kihila J. M. (2024). Solid waste collection in the informal settlements of African cities: a regulatory dilemma for actor's participation and collaboration in Kampala. Urban Forum.

[cit98] Geenen S. (2012). A dangerous bet: The challenges of formalizing artisanal mining in the Democratic Republic of Congo. Resour. Policy.

[cit99] Mittal K. M., Timme M., Schröder M. (2024). Efficient self-organization of informal public transport networks. Nat. Commun..

[cit100] Polese A., Rekhviashvili L., Morris J. (2016). Informal governance in urban spaces: Power, negotiation and resistance among Georgian street vendors. Geogr. Res. Forum.

[cit101] Koen L., Fourie E. S. (2023). Waste pickers and the law: Contradictory and fragmented regulation in three metropolitan municipalities. Dev. South. Afr..

[cit102] Gilson G. G., Garrick D. (2024). Co-producing water service delivery: a scoping review and typology of informal water vending in Sub-Saharan Africa. Environ. Res. Lett..

[cit103] Priadi C. R., Suleeman E., Darmajanti L., Putri G. L., Genter F., Foster T., Willetts J. (2024). Policy and regulatory context for self-supplied drinking water services in two cities in Indonesia: Priorities for managing risks. Environ. Dev..

[cit104] Becchis F. , Where Things Happen: The Local Dimension in Regulation, in The Political Economy of Local Regulation: Theoretical Frameworks and International Case Studies, ed. A. Asquer, F. Becchis and D. Russolillo, Palgrave Macmillan, 2017, pp. 95–112, 10.1057/978-1-137-58828-9

[cit105] Yang J., Zhang J., Guo C., Tan R., Yu M. (2021). Incentive or punitive measure? Analysis of environmental regulations in construction and demolition waste recycling. Math. Probl. Eng..

[cit106] Tucker J. L. (2024). Barriers to Inclusive Recycling in Asunción, Paraguay: A Just Transition?. Dev. Change.

[cit107] Guillen M. D. V. , Gaspay S. M., Mateo-Babiano I. and Schneck J. A. T., Formalizing the Informal in Public Transportation: The Case of Motorcycle Taxis Transport Network Service in the Philippines. Conference paper, Thredbo 2025 Conference International Conference Series on Competition and Ownership in Land Passenger Transport, Cape Town, 2024

[cit108] Ferro P. S., Behrens R., Wilkinson P. (2013). Hybrid urban transport systems in developing countries: Portents and prospects. Res. Transp. Econ..

[cit109] Bhatt J. (2014). Comparison of small-scale providers' and utility performance in urban water supply: the case of Maputo, Mozambique. Water Policy.

[cit110] Bansah K. J. (2023). Artisanal and small-scale mining formalization in Ghana: the government's approach and its implications for cleaner and safer production. J. Cleaner Prod..

[cit111] Weissenberger L. T., Elliott S. J. (2024). Gender dimensions of water vending in LMICs: A scoping review. Water Secur..

[cit112] Castells M. and Portes A., World underneath: The origins, dynamics, and effects of the informal economy, in The informal economy: Studies in advanced and less developed countries, ed. A. Portes, M. Castells and L. A. Benton, Johns Hopkins University Press, 1989

[cit113] De Soto H. , The mystery of capital: Why capitalism triumphs in the West and fails everywhere else, Basic books, 2000

[cit114] Karpouzoglou T., Vij S., Blomkvist P., Juma B., Narain V., Nilsson D., Sitoki L. (2023). Analysing water provision in the critical interface of formal and informal urban water regimes. Water Int..

[cit115] Chen M. and Carré F., The informal economy revisited: Examining the past, envisioning the future, Routledge, 2020, 10.4324/9780429200724

[cit116] Batréau Q., Bonnet F. (2016). Managed informality: Regulating street vendors in Bangkok. City Community.

[cit117] Bulkan J., Palmer J. (2016). Rentier nation: Landlordism, patronage and power in Guyana's gold mining sector. Extr. Ind. Soc..

[cit118] ILO , Innovative approaches to addressing informality and promoting the transition to formality for decent work, International Labour Conference 113th Session, International Labour Organization, 2025

[cit119] Ananya T. H. , Hossain A. Z. N., Ahmed T., Poustie M., Zaqout M. and Barrington D., Unforeseen adverse outcomes associated with formalization of fecal sludge emptying services in municipalities of Bangladesh, Sanitation Workers Research Awardees – Best Original Research Abstract, 2024, https://sanitationworkers.susana.org/blog/32-sanitation-workers-research-awards-2024-meet-the-awardees, (accessed 10 September 2025)

[cit120] Simpson M., Oduro-Appiah K., Gunsilius E., Dias S. M., Scheinberg A. (2024). Shifting perceptions of informal operators in the service and value chains: A retrospective of 40 years of observation and advocacy for informal recyclers and waste service providers, through the eyes of five global participant-researchers. Waste Manage. Res..

[cit121] SASLN , Occupational Safety and Health of Sanitation Workers: Access to Protective Equipment and Services, South Asian Sanitation Worker and Labour Network, 2025

[cit122] Gerlach E., Franceys R. (2010). Regulating water services for all in developing economies. World Dev..

[cit123] Rosaldo M. (2021). Problematizing the “informal sector”: 50 years of critique, clarification, qualification, and more critique. Sociol. Compass.

[cit124] Hilson G., Sauerwein T., Owen J. (2020). Large and artisanal scale mine development: The case for autonomous co-existence. World Dev..

[cit125] Ofosu G., Arthur-Holmes F., Siaw D., Sarpong D. (2025). Friends or foes: Can large-scale mining companies partner with small-scale miners? Yes, they can?. J. Rural Stud..

[cit126] Behrens R., McCormick D., Orero R., Ommeh M. (2017). Improving paratransit service: Lessons from inter-city matatu cooperatives in Kenya. Transp. Policy.

[cit127] Malasan P. L. (2019). The untold flavour of street food: Social infrastructure as a means of everyday politics for street vendors in Bandung, Indonesia. Asia Pac. Viewpoint.

[cit128] Calderón Márquez A. J., Silva de Souza Lima Cano N., Rutkowski E. W. (2021). Inclusion of waste pickers into municipal waste management systems: A comparison between Colombia and Brazil. J. Environ. Dev..

[cit129] Recio R. B. (2021). How can street routines inform state regulation? Learning from informal traders in Baclaran, Metro Manila. Int. Dev. Plan. Rev..

[cit130] George C., Msoka C. T., Makundi H. (2023). Formalisation of street vending in Dar es Salaam: Implementation and enforcement of the Wamachinga identity card initiative. Forum Dev. Stud..

[cit131] Ntramah S., Peters K., Jenkins J., Mugisha M. M., Chetto R., Owino F., Hayombe P. O., Opiyo P., Santos R. T., Johnson T. (2023). Safety, health and environmental impacts of commercial motorcycles in Sub-Saharan African cities. Urban Plan. Transp. Res..

[cit132] Kusakabe K. , Policy issues on street vending: an overview of studies in Thailand, Cambodia and Mongolia. Informal economy, poverty and employment, International Labour Organization, 2006

[cit133] Rodríguez L. A., Velez M. A., Pfaff A. (2021). Leaders' distributional and efficiency effects in collective responses to policy: Lab-in-field experiments with small-scale gold miners in Colombia. World Dev..

[cit134] Cardoso M. E. G. and Hilson G., Cooperatives as a centrepiece for formalizing small-scale mining in sub-Saharan Africa: Rationale, benefits and limitations, in Routledge Handbook of the Extractive Industries and Sustainable Development, ed. N. Yakovleva and E. Nickless, 2022, Routledge, pp. 419–439, 10.4324/9781003001317

[cit135] Gómez-Maldonado A., Ospina-Espita L. C., Rodríguez-Lesmes P., Rodríguez-Rodriguez M. A. (2023). Barriers and opportunities for waste pickers within solid waste management policy in Colombia. Waste Manage..

[cit136] Dias S. M., Ogando A. C. (2015). Rethinking gender and waste: exploratory findings from participatory action research in Brazil. Work Organ. Labour Glob..

[cit137] WHO , Global research agenda for improving the health safety and dignity of sanitation workers, World Health Organization, 2022

[cit138] Bushele M. , Khalifa L. and Singh K., From informal manual emptiers to emerging role models: the journey of Tanzania's Watu Kazi group, The Sanitation Workers Knowledge and Learning Hub, 2024, https://sanitationworkers.susana.org/blog/33-from-informal-manual-emptiers-to-emerging-role-models-the-journey-of-tanzania-s-watu-kazi-group#

[cit139] Gofen A., Moseley A., Thomann E., Kent Weaver R. (2021). Behavioural governance in the policy process: introduction to the special issue. J. Eur. Public Policy.

[cit140] Barak-Corren N., Kariv-Teitelbaum Y. (2021). Behavioral responsive regulation: bringing together responsive regulation and behavioral public policy. Regul. Gov..

[cit141] Kogler C. , Olsen J., Osman M. and Zeelenberg M., The effect of transparent unequal penalty rates on safety compliance for different-sized businesses, Behavioural Public Policy, 2023, pp. 1–17, 10.1017/bpp.2022.42

[cit142] Hortal A. and Martínez A. P., Behavioral strategies for reducing corruption: from regulation to choice architecture, Behavioural Public Policy, 2024, pp. 1–18, 10.1017/bpp.2024.48

[cit143] Molopa S. T. (2024). Artificial intelligence-based literature review adaptation. S. Afr. J. libr. Inf. Sci..

[cit144] Baldwin R. , Cave M. and Lodge M., Understanding regulation: theory, strategy, and practice, Oxford University Press, 2nd edn, 2011, 10.1093/acprof:osobl/9780199576081.001.0001

[cit145] Taylor C., Pollard S. J. T., Rocks S. A., Angus A. J. (2012). Selecting policy instruments for better environmental regulation: a critique and future research agenda. Environ. Policy Gov..

[cit146] Braithwaite J. , Types of responsiveness, in Regulatory theory: Foundations and applications, ed. P. Drahos, ANU Press, 2017, pp. 117–132, 10.22459/RT.02.2017

[cit147] Corry D. , Delivering economic growth and nature recovery: An independent review of Defra's regulatory landscape, Department for Environment, Food and Rural Affairs, UK Government, London, 2025

[cit148] Kanbur R. (2017). Informality: Causes, consequences and policy responses. Rev. Dev. Econ..

