# Supplementary material for: Using analogous sectors to build good practice resources in regulation of urban faecal sludge emptying and transport
Source: Environ Sci (Camb). 2026 Jul 22;12(10):3149–70. doi: 10.1039/d5ew01108f (PMC13559381; doi:10.1039/d5ew01108f)
Supplement: Supplementary file 1 [file EW-012-D5EW01108F-s001.pdf]

Supplementary Information (SI) for Environmental Science: Water Research & Technology.
This journal is © The Royal Society of Chemistry 2026

Supplementary materials:
Using analogous sectors to build good practice resources in regulation of urban faecal sludge emptying and transport

Contents

S.1 Shortlisting analogous sectors 1

S.2 Rapid literature review protocol..13

S.3: Sector summaries 19

S.3-1: Street Food vendors 19

S.3-2: Construction waste management (illegal dumping). 29

S.3-3: Artisanal mining 39

S.3-4: Informal transport. 52

S.3-5: Off grid water...62

S3-6: Municipal solid waste management (waste pickers) 73

S.1 Shortlisting analogous sectors

Table S.1: Analogous sectors identified and shared challenges with FS emptying and transport

The specificity for each sector suggested in the survey was highly variable, further subsets were developed based on an initial review.

Challenge	**The service is viewed as low status (and in some places - disgusting), impacting front line, and managers and leadership**		**There are typically low levels of trust between the authorities and workers or enterprises providing services.**		**Cost: The costs of compliance for service providers: high operating costs, largely in (hard to monitor) transport and disposal, which increase in low-income areas.**				**Enabling environment:**				**Referenced in categorisation**
Sector<br>(challenges linked)	**Low status**	**Considered disgusting**	**Low trust between regulators and implementors**	**High levels of informality**	**Invisible / Low visibility**	**High operating costs**	**Dispersed – hard to monitor / control**	**Compliance costs increase in low-income areas**	**Resources insufficient and not allocated equitably**	**Public expectations, and political will, is low**	**Lack of integrated planning across sectors and urban areas**	**Unclear, or actively obstructive legal and regulatory framework**	
Taxation of small and medium enterprises (5)			X	X	X		X					X	Kamleitner, et al., 2012<br>Resnick, 2021
Insurance / finance of SMEs<br>(4)			X	X			X					X	Rao et al, 2023<br>Falkner et al., 2015
Street food vendors (11)	X		X	X	X	X	X	X	X	X	X	X	Bouafou,et al., 2021<br>Abrahale et al., 2019<br>Sepadi and Nkosi, 2022<br>Hanser, 2021
Off grid electricity (7)				X	X		X	X	X		X	X	Bhattacharyya et al., 2013<br>Babayomi, et al., 2023
Informal transport systems (10)	X		X	X	X	X	X	X	X		X	X	Cervero et al., 2007<br>Ehebrecht et al., 2018

Challenge	**The service is viewed as low status (and in some places - disgusting), impacting front line, and managers and leadership**		**There are typically low levels of trust between the authorities and workers or enterprises providing services.**		**Cost: The costs of compliance for service providers: high operating costs, largely in (hard to monitor) transport and disposal, which increase in low-income areas.**				**Enabling environment:**				**Referenced in categorisation**
Sector<br><br>(challenges linked)	**Low status**	**Considered disgusting**	**Low trust between regulators and implementors**	**High levels of informality**	**Invisible / Low visibility**	**High operating costs**	**Dispersed – hard to monitor / control**	**Compliance costs increase in low-income areas**	**Resources insufficient and not allocated equitably**	**Public expectations, and political will, is low**	**Lack of integrated planning across sectors and urban areas**	**Unclear, or actively obstructive legal and regulatory framework**	
Artisanal mining (10)	X		X	X	X	X	X		X	X	X	X	Boldy et al., 2021<br><br>Schwartz, et al., 2021
Basic health care / Vaccination (4)						X	X		X			X	Debie et al., 2022<br><br>Roncarolo, et al. 2017
Stigmatised/ taboo health issues (9)	X	X	X		X	X	X		X	X		X	Ferguson,et al., 2022<br><br>Van Brakel et al., 2006
Conservation / Environmental protection (5)			X				X	X	X			X	de Miranda Ribeiro, 2015<br><br>Khanam, et al, 2023
Food production (2)											X	X	Wahbeh et al., 2022<br><br>Yadav, et al., 2022.
Education (3)						X		X				X	Nkrumah and Sinha, 2020<br><br>Hoppers, 2006
Housing provision								X	X		X	X	Howden-

Challenge	The service is viewed as low status (and in some places - disgusting), impacting front line, and managers and leadership		There are typically low levels of trust between the authorities and workers or enterprises providing services.		Cost: The costs of compliance for service providers: high operating costs, largely in (hard to monitor) transport and disposal, which increase in low-income areas.				Enabling environment:				Referenced in categorisation
Sector<br><br>(challenges linked)	**Low status**	**Considered disgusting**	**Low trust between regulators and implementors**	**High levels of informality**	**Invisible / Low visibility**	**High operating costs**	**Dispersed – hard to monitor / control**	**Compliance costs increase in low-income areas**	**Resources insufficient and not allocated equitably**	**Public expectations, and political will, is low**	**Lack of integrated planning across sectors and urban areas**	**Unclear, or actively obstructive legal and regulatory framework**	
and quality (4)													Chapman et al, 2023<br><br>WHO, 2021
Informal or off-grid water supply (10)	X		X	X	X	X	X	X	X		X	X	Amankwaa, et al. 2021<br><br>Dos Santos et al., 2017
Cleaning public areas / pest control (5)	X	X			X		X	X				X	Feldmann and Vogler, 2021<br><br>Sweeney, 2023
Industrial pollution (4)			X			X	X					X	Malik et al., 2022<br><br>Singh et al., 2023
Construction waste management (fly tipping) (10)	X		X	X	X	X	X	X		X	X	X	Cook et al., 2022<br><br>Niyobuhungiro and Schenck, 2022
Wastewater management (5)		X					X		X		X	X	Preisner et al., 2020<br><br>Muzioreva et al., 2022
Hazardous waste		X					X	X	X		X	X	Fazzo et al.,

Challenge	**The service is viewed as low status (and in some places - disgusting), impacting front line, and managers and leadership**		**There are typically low levels of trust between the authorities and workers or enterprises providing services.**		**Cost: The costs of compliance for service providers: high operating costs, largely in (hard to monitor) transport and disposal, which increase in low-income areas.**				**Enabling environment:**				**Referenced in categorisation**
Sector<br><br>(challenges linked)	**Low status**	**Considered disgusting**	**Low trust between regulators and implementors**	**High levels of informality**	**Invisible / Low visibility**	**High operating costs**	**Dispersed – hard to monitor / control**	**Compliance costs increase in low-income areas**	**Resources insufficient and not allocated equitably**	**Public expectations, and political will, is low**	**Lack of integrated planning across sectors and urban areas**	**Unclear, or actively obstructive legal and regulatory framework**	
management (6)													2017<br><br>Akpan and Olukanni, 2020
Air pollution (2)					X		X						Jonidi Jafari et al., 2021<br><br>Feng et al., 2024.
Agricultural pollution (3)			X			X	X						Zahoor and Mushtaq, 2023<br><br>Blackstock et al., 2010
Solid waste management; waste pickers (11)	X	X	X	X	X		X	X	X	X	X	X	Muheirwe et al., 2022<br><br>Zolnikov et al., 2021
Sachet economy (3)					X		X					X	Stoler, 2017<br><br>Ayeleru, et al., (2020).
Emerging technology (incl. IT) (3)							X				X	X	Székely, 2011<br><br>Lewallen, 2021
Telecommunications (1)												X	Cambini and Jiang, 2009<br><br>Dike and Rose, 2017

Challenge	The service is viewed as low status (and in some places - disgusting), impacting front line, and managers and leadership		There are typically low levels of trust between the authorities and workers or enterprises providing services.		Cost: The costs of compliance for service providers: high operating costs, largely in (hard to monitor) transport and disposal, which increase in low-income areas.				Enabling environment:				Referenced in categorisation
Sector<br><br>(challenges linked)	Low status	Considered disgusting	Low trust between regulators and implementors	High levels of informality	Invisible / Low visibility	High operating costs	Dispersed – hard to monitor / control	Compliance costs increase in low-income areas	Resources insufficient and not allocated equitably	Public expectations, and political will, is low	Lack of integrated planning across sectors and urban areas	Unclear, or actively obstructive legal and regulatory framework	
Recycling and resource recovery (6)						X	X	X		X	X	X	Aguilar et al., 2022<br><br>Zhang et al., 2024

References

Abrahale, K., Sousa, S., Albuquerque, G., Padrão, P., & Lunet, N. (2019). Street food research worldwide: a scoping review. Journal of Human Nutrition and Dietetics, 32(2), 152-174.

Aguilar, M. G., Jaramillo, J. F., Ddiba, D., Páez, D. C., Rueda, H., Andersson, K., & Dickin, S. (2022). Governance challenges and opportunities for implementing resource recovery from organic waste streams in urban areas of Latin America: insights from Chía, Colombia. Sustainable Production and Consumption, 30, 53-63.

Akpan, V. E., & Olukanni, D. O. (2020). Hazardous waste management: An African overview. Recycling, 5(3), 15.

Amankwaa, G., Heeks, R., & Browne, A. L. (2021). Digital Innovations and Water Services in Cities of the Global South: A Systematic Literature Review. Water Alternatives, 14(2).

Ayeleru, O. O., Dlova, S., Akinribide, O. J., Ntuli, F., Kupolati, W. K., Marina, P. F., ... & Olubambi, P. A. (2020). Challenges of plastic waste generation and management in sub-Saharan Africa: A review. Waste Management, 110, 24-42.

Babayomi, O. O., Olubayo, B., Denwigwe, I. H., Somefun, T. E., Adedoja, O. S., Somefun, C. T., ... & Attah, A. (2023). A review of renewable off-grid mini-grids in Sub-Saharan Africa. Frontiers in Energy Research, 10, 1089025.

Bhattacharyya, S. C. (2013). Financing energy access and off-grid electrification: A review of status, options and challenges. Renewable and Sustainable Energy Reviews, 20, 462-472.

Blackstock, K. L., Ingram, J., Burton, R., Brown, K. M., & Slee, B. (2010). Understanding and influencing behaviour change by farmers to improve water quality. Science of the total environment, 408(23), 5631-5638.

Boldy, R., Santini, T., Annandale, M., Erskine, P. D., & Sonter, L. J. (2021). Understanding the impacts of mining on ecosystem services through a systematic review. The Extractive Industries and Society, 8(1), 457-466.

Bouafou, K. G. M., Beugré, G. F. C., & Amani, Y. C. (2021). Street food around the world: A review of the literature. Journal of Service Science and Management, 14(6), 557-575.

Cambini, C., & Jiang, Y. (2009). Broadband investment and regulation: A literature review. Telecommunications Policy, 33(10-11), 559-574.

Cervero, R., & Golub, A. (2007). Informal transport: A global perspective. Transport policy, 14(6), 445-457.

Cook, E., Velis, C. A., & Black, L. (2022). Construction and demolition waste management: a systematic scoping review of risks to occupational and public health. Frontiers in Sustainability, 3, 924926.

Debie, A., Khatri, R. B., & Assefa, Y. (2022). Successes and challenges of health systems governance towards universal health coverage and global health security: a narrative review and synthesis of the literature. Health research policy and systems, 20(1), 50.

de Miranda Ribeiro, F., & Kruglianskas, I. (2015). Principles of environmental regulatory quality: a synthesis from literature review. Journal of Cleaner Production, 96, 58-76.

Dike, M. C., & Rose, E. L. (2017). Internationalization of mobile telecommunications: a systematic literature review. Review of International Business and Strategy, 27(3), 308-321.

Dos Santos, S., Adams, E. A., Neville, G., Wada, Y., De Sherbinin, A., Bernhardt, E. M., & Adamo, S. B. (2017). Urban growth and water access in sub-Saharan Africa: Progress, challenges, and emerging research directions. Science of the Total Environment, 607, 497-508.

Ehebrecht, D., Heinrichs, D., & Lenz, B. (2018). Motorcycle-taxis in sub-Saharan Africa: Current knowledge, implications for the debate on “informal” transport and research needs. Journal of transport geography, 69, 242-256.

Falkner, E. M., & Hiebl, M. R. (2015). Risk management in SMEs: a systematic review of available evidence. The Journal of Risk Finance, 16(2), 122-144.

Fazzo, L., Minichilli, F., Santoro, M., Ceccarini, A., Della Seta, M., Bianchi, F., ... & Martuzzi, M. (2017). Hazardous waste and health impact: a systematic review of the scientific literature. Environmental health, 16, 1-11.

Feldmann, F., & Vogler, U. (2021). Towards sustainable performance of urban horticulture: ten challenging fields of action for modern integrated pest management in cities. Journal of Plant Diseases and Protection, 128(1), 55-66.

Feng, T., Sun, Y., Shi, Y., Ma, J., Feng, C., & Chen, Z. (2024). Air pollution control policies and impacts: A review. Renewable and Sustainable Energy Reviews, 191, 114071.

Ferguson, L., Gruskin, S., Bolshakova, M., Yagyu, S., Fu, N., Cabrera, N., ... & Hempel, S. (2022). Frameworks and measures for HIV-related internalized stigma, stigma and discrimination in healthcare and in laws and policies: a systematic review. Journal of the International AIDS Society, 25, e25915.

Howden-Chapman, P., Bennett, J., Edwards, R., Jacobs, D., Nathan, K., & Ormandy, D. (2023). Review of the impact of housing quality on inequalities in health and well-being. Annual review of public health, 44(1), 233-254.

Hoppers, W. (2006). Non-Formal Education and Basic Education Reform: A Conceptual Review. International Institute for Educational Planning (IIEP) UNESCO. 7-9 rue Eugene-Delacroix, 75116 Paris, France.

Jonidi Jafari, A., Charkhloo, E., & Pasalari, H. (2021). Urban air pollution control policies and strategies: a systematic review. Journal of Environmental Health Science and Engineering, 19, 1911-1940.

Kamleitner, B., Korunka, C., & Kirchler, E. (2012). Tax compliance of small business owners: A review. International Journal of Entrepreneurial Behavior & Research, 18(3), 330-351.

Khanam, Z., Sultana, F. M., & Mushtaq, F. (2023). Environmental Pollution Control Measures and Strategies: An Overview of Recent Developments. Geospatial Analytics for Environmental Pollution Modeling: Analysis, Control and Management, 385-414.

Lewallen, J. (2021). Emerging technologies and problem definition uncertainty: The case of cybersecurity. Regulation & Governance, 15(4), 1035-1052.

Malik, S., Kishore, S., Prasad, S., & Shah, M. P. (2022). A comprehensive review on emerging trends in industrial wastewater research. Journal of Basic Microbiology, 62(3-4), 296-309.

Muheirwe, F., Kombe, W., & Kihila, J. M. (2022). The paradox of solid waste management: A regulatory discourse from Sub-Saharan Africa. Habitat International, 119, 102491.

Muzioreva, H., Gumbo, T., Kavishe, N., Moyo, T., & Musonda, I. (2022). Decentralized wastewater system practices in developing countries: A systematic review. Utilities Policy, 79, 101442.

Niyobuhungiro, R. V., & Schenck, C. J. (2022). A global literature review of the drivers of indiscriminate dumping of waste: Guiding future research in South Africa. Development Southern Africa, 39(3), 321-337.

Nkrumah, R. B., & Sinha, V. (2020). Revisiting global development frameworks and research on universal basic education in Ghana and Sub-Saharan Africa: a review of evidence and gaps for future research. Review of Education, 8(3), 733-764.

Preisner, M., Neverova-Dziopak, E., & Kowalewski, Z. (2020). An analytical review of different approaches to wastewater discharge standards with particular emphasis on nutrients. Environmental Management, 66(4), 694-708

Rao, P., Kumar, S., Chavan, M., & Lim, W. M. (2023). A systematic literature review on SME financing: Trends and future directions. Journal of Small Business Management, 61(3), 1247-1277.

Resnick, D. (2021). Taxing informality: Compliance and policy preferences in urban Zambia. The Journal of Development Studies, 57(7), 1063-1085.

Roncarolo, F., Boivin, A., Denis, J. L., Hébert, R., & Lehoux, P. (2017). What do we know about the needs and challenges of health systems? A scoping review of the international literature. BMC Health Services Research, 17, 1-18.

Schwartz, F. W., Lee, S., & Darrah, T. H. (2021). A review of the scope of artisanal and small-scale mining worldwide, poverty, and the associated health impacts. GeoHealth, 5(1), e2020GH000325.

Singh, B. J., Chakraborty, A., & Sehgal, R. (2023). A systematic review of industrial wastewater management: Evaluating challenges and enablers. Journal of Environmental Management, 348, 119230.

Stoler, J. (2017). From curiosity to commodity: a review of the evolution of sachet drinking water in West Africa. Wiley Interdisciplinary Reviews: Water, 4(3), e1206.

Sweeney, K. (2023). Present and Future Approaches to Urban Pest Management: A Global Pesticide Regulatory Perspective. In Urban Pest Management: An Environmental Perspective (pp. 121-141). GB: CABI.

Székely, I., Dániel Szabó, M., & Vissy, B. (2011). Regulating the future? Law, ethics, and emerging technologies. Journal of Information, Communication and Ethics in Society, 9(3), 180-194.

Van Brakel, W. H. (2006). Measuring health-related stigma—a literature review. Psychology, health & medicine, 11(3), 307-334.

Wahbeh, S., Anastasiadis, F., Sundarakani, B., & Manikas, I. (2022). Exploration of food security challenges towards more sustainable food production: A systematic literature review of the major drivers and policies. Foods, 11(23), 3804.

World Health Organization. (2021). Policies, regulations and legislation promoting healthy housing: a review.

Yadav, V. S., Singh, A. R., Gunasekaran, A., Raut, R. D., & Narkhede, B. E. (2022). A systematic literature review of the agro-food supply chain: Challenges, network design, and performance measurement perspectives. Sustainable Production and Consumption, 29, 685-704.

Zahoor, I., & Mushtaq, A. (2023). Water pollution from agricultural activities: A critical global review. Int. J. Chem. Biochem. Sci, 23(1), 164-176.

Zhang, Z., Chen, Z., Zhang, J., Liu, Y., Chen, L., Yang, M., ... & Yap, P. S. (2024). Municipal solid waste management challenges in developing regions: A

comprehensive review and future perspectives for Asia and Africa. Science of the Total Environment, 172794.

Zolnikov, T. R., Furio, F., Cruvinel, V., & Richards, J. (2021). A systematic review on informal waste picking: Occupational hazards and health outcomes. Waste Management, 126, 291-308.

S.2 Rapid literature review protocol

Review Team

The literature review is completed by the first author. The paper's co-authors from Cranfield University, Dr Alison Parker and Dr Paul Leinster, who approved this protocol, completed spot checks during the screening process and reviewed the narrative summaries.

Rationale and objectives of the review

We aim to identify and assess potential good practice which has not yet been widely considered in regulation of faecal sludge emptying and transport, and which may be useful in building compliance. The review aims to identify learning from sectors analogous to faecal sludge emptying and transport around how to build compliance.

A total of 25 potential analogous sectors were identified through the Delphi study completed in Grisaffi et al. (2025). Initial scoping was done on 10 of these; taxation of small and medium enterprises, insurance / finance of SMEs, street food vendors, off grid electricity, informal transport systems, artisanal mining, basic health care (split up into compliance for healthcare institutions, vaccination and hand hygiene), stigmatised/taboo health issues (split up into HIV/AIDS treatment compliance and the intersection of stigma and policy), education (largely institutional inspections) cleaning public areas / pest control. This initial scoping highlighted the difficulty of identifying relevant literature with such a loose scope.

These 25 potential sectors were therefore shortlisted to six, using the priority challenges identified in Grisaffi et al. (2025). The six sectors are given in Table S2-1.

A rapid review is being completed, in place of a systematic or scoping review due to the multiple sectors being assessed. The methodology presented in this protocol is for a rapid review which allows the most critical studies to be identified from each analogous sector, balancing breadth and depth. The rapid review takes the basic definition proposed by Hamel et al. (2021) 'A rapid review is a form of knowledge synthesis that accelerates the process of conducting a traditional systematic review through streamlining or omitting a variety of methods to produce evidence in a resource-efficient manner'. Hamel et al. (2021) note that there are currently not agreed approaches for what or how to streamline. The main areas of streamlining

were to: (i) replace multiple database screening with the use of AI tools, (ii) narrow the range of keywords and (iii) minimise quality checks. The use of seed papers in AI is expected to reduce the challenges of identifying the correct keywords when working in sectors where the author group have very little experience.

This protocol was developed and piloted using one example analogous sector using Street Food as a trial. It was then simplified further and implemented for further analogous sectors. The review validated the use of AI; the AI search engines typically identified at least half of the relevant papers finally included and there was limited overlap with the articles identified through Scopus or Google Scholar.

Table S2-1 Analogous Sectors and Search String used

Subgroup	Sector	Search string for sector
Small / informal businesses	Street food vendors	Street AND [Food OR Vendor*]
	Informal transport systems	Informal* AND [Mobility OR Transport]
	Artisanal mining	[Artisanal OR small scale OR illegal] AND mining
Essential public services	Informal or off-grid water supply	[vendor OR informal OR illegal OR off-grid] AND [water AND [network OR supply]]
Dispersed pollution risks	Construction waste disposal – focussed on illegal dumping	[construction AND waste AND [management OR disposal]]
	Municipal solid waste management – focussed on waste pickers	[municipal AND solid AND waste AND management] OR [waste AND pickers]

Inclusion and exclusion criteria

The inclusion and exclusion criteria are structured as shown in Table S2-2 using CIMO criteria (Denyer et al., 2008). The initial outcome criteria included only measured impacts on compliance. This was relaxed to also include postulated impacts due to the lack of studies with measured impacts on compliance.

Table S2-2: Inclusion and exclusion criteria

	Inclusion	Exclusion
**Context: The social and organizational setting in which the phenomenon occurs.**	Where there is a compliance challenge relevant to a given analogous sector	Compliance for high status and high profit services only delivered to high income groups

**Intervention: The actions are taken to address or influence the phenomenon.**	Any action with an explicit aim to increase compliance.	Assessing current situation with no attempt to change
**Mechanisms: The underlying processes or mechanisms that drive change within the phenomenon.**	Building long term compliance.	Short term actions with no long-term implication.
**Outcome: The resulting changes occur due to the intervention and the underlying mechanisms**	Postulated or measured impact - positive or negative - on compliance.	No postulated or measured impact on compliance.

Databases and key terms of the literature search; Process and tools for study selection

Identifying seed papers: Seed papers are identified through Scopus and Google Scholar, identified as having a wide journal range and coverage (Falagas et al., 2008). Searching in: Keywords, title and abstract using the following search terms:

- [*Sector*] AND [Regulation OR Compliance] AND [impact OR evidence OR change OR increase]

[*Sector*] terms are given in Table S2-1.

Manual screening is used to identify at least five relevant papers, meeting the criteria in Table S2-2, which are then used as seed papers.

Identifying and screening literature using AI: The seed papers are then uploaded to two AI literature screening tools; Research rabbit (https://www.researchrabbit.ai/), and Inciteful (https://inciteful.xyz/). Research Rabbit is selected based on recommendations from Ashkanani et al. (2024). Inciteful is added based on the functionality in giving an over of the sector; the website provides the top 50 similar papers, review papers, and influential papers. Litmaps (https://www.litmaps.com/) was also trialled during the pilot but did not identify any new papers. Maps of the literature produced through the software are visually inspected to identify whether the literature is fragmented, and identify any outliers not linked to the main groups of texts.

The top recommendations from Research Rabbit and Inciteful (for similar, most influential and review articles) would be saved as CSV files. Excel would be used to

remove duplicates. Abstracts and titles would be screened for relevance and a full text review would then be completed. If no papers are identified which meet the criteria in Table S2-2 then the seed papers would be reviewed, and a second iteration of the process would be attempted.

Data extraction and quality assessment.

The full text review will start with recent systematic reviews and use these as a base, then work forward and updating them more recent relevant primary studies (King et al., 2022). The full text reviews would be continued working backwards from the systematic reviews. Sufficient full text review would be completed to reach data saturation, taken as the point where no new insights are being gained (Sebele-Mpofu, 2020). Where there are distinct geographical splits in the literature identified then recent papers would be selected across each geographical zone. The remaining eligible papers would be rapidly screened to check for new insights and then marked as superseded.

The following data is extracted.

- Information about the article (author(s), year of publication, title, source, number of citations)
- Type of article - literature review, observational, experimental
- Location (geographical, target population demographics)
- Sector
- Key findings on regulation or compliance.

Quality assessment would be limited to an estimated level of confidence in the evidence and selecting papers only from journals which are indexed in the major bibliographic databases, are peer reviewed and have an impact factor of more than one. The above approach means that most practitioner literature is excluded.

Synthesis and review

The findings are synthesised by the main areas of the holistic model of compliance presented in Grisaffi et al. (2025):

- Target group: Motivations, Knowledge of the rules and capacity to comply
- Perceptions of, and relationship with, the regulator
- Resourcing and supporting regulators
- Enabling environment
- External actors

The narrative summary produced, describing the learning around building or eroding compliance, are shared with sector specialists for review and validation. Corresponding authors from all relevant papers published within the last 5 years are contacted via email and requested to for feedback on (i) whether this summary is an accurate reflection of current discussions on regulation of off-grid water providers and (ii) identification of any major omissions or misunderstandings. Summary was presented as a text document and, where requested, as an online structured survey or a presentation during an online discussion.

Feedback is incorporated into the final summaries. The summaries were shared with all corresponding authors cited in the rapid literature review. These sector specialists were asked to check for completeness and understanding of current approaches to build compliance in their sector. Invitations to review were also extended to co-authors cited in the literature review until at least three reviews had been completed for each sector.

References

Ashkanani, Z., Mohtar, R., Al-Enezi, S., Smith, P. K., Calabrese, S., Ma, X., & Abdullah, M. (2024). AI-assisted systematic review on remediation of contaminated soils with PAHs and heavy metals. *Journal of Hazardous Materials*, 133813.

Denyer, D., Tranfield, D., & Van Aken, J. E. (2008). Developing design propositions through research synthesis. *Organization studies*, 29(3), 393-413.

Falagas, M. E., Pitsouni, E. I., Malietzis, G. A., & Pappas, G. (2008). Comparison of PubMed, Scopus, web of science, and Google scholar: strengths and weaknesses. *The FASEB journal*, 22(2), 338-342.

Grisaffi, C., Leinster, P., Mugo, K., Drabble, S., & Parker, A. (2025). Safe faecal sludge emptying and transport: compliance challenges and models for a public good. *H2Open Journal*, h2oj2025041.

Hamel, C., Michaud, A., Thuku, M., Skidmore, B., Stevens, A., Nussbaumer-Streit, B., & Garritty, C. (2021). Defining rapid reviews: a systematic scoping review and thematic analysis of definitions and defining characteristics of rapid reviews. *Journal of Clinical Epidemiology*, 129, 74-85.

King, V. J., Stevens, A., Nussbaumer-Streit, B., Kamel, C., & Garritty, C. (2022). Paper 2: Performing rapid reviews. *Systematic Reviews*, 11(1), 151.

Sebele-Mpofu, F. Y. (2020). Saturation controversy in qualitative research: Complexities and underlying assumptions. A literature review. *Cogent Social Sciences*, 6(1), 1838706.

S.3: Sector summaries

S.3-1: Street Food vendors

Literature identified

Sources and steps are given in the PRISMA diagram below. The search string used was: [Street AND [Food OR Vendor*]] AND [Regulation OR Compliance] AND [impact OR evidence OR change OR increase]. Compliance is considered around two main areas; hygienic practices for food safety, and geographic restrictions or allowed workplace.

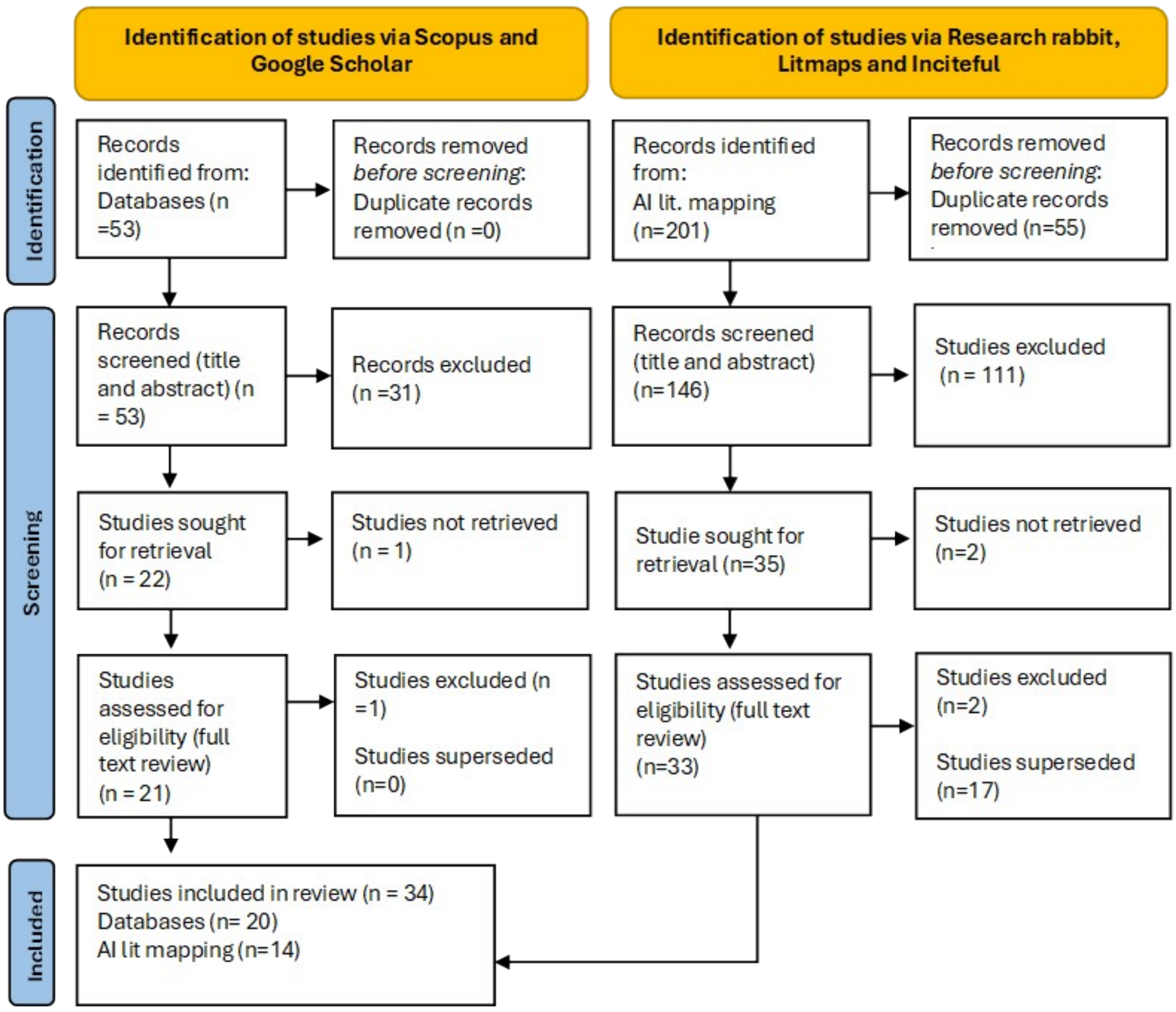

As highlighted in a 2015 review, the limited literature on this sector does not identify effective regulatory approaches for locations where risks are pervasive, costs of compliance are high, and enforcement capacity is weak (Grace, 2015). The majority of papers identified were limited to quantifying current practices and challenges, often focussing on the narrow experience of street vendors as individuals rather than wider structural issues (Tafti, 2019). Full text review identified five papers which claimed to have identified impacts on compliance, however none of these were experimental studies able to demonstrate causality. A wider range of articles are therefore included below all of which include postulated impacts on compliance. Literature identified was predominantly from East and Southern Africa, and Southeast Asia.

These studies cover a wide range of contexts and a huge variation both within and across countries and the synthesis acts to flatten out these differences. In order to address this limitation, the review is largely limited to only the points which are raised across multiple locations. This makes the review more generic, but increases the likelihood that findings are transferable.

Street food vendors were chosen as an analogous sector to fecal sludge emptying and transport because they have similar compliance challenges around the knowledge, attitudes and practices (KAP) of safe working. There are high numbers of KAP studies on street food vendors, which were used in the selection of the sector. These KAP studies were excluded from the literature review as they do not describe how to increase compliance.

The rapid review findings incorporate the additional 5 recommended references from Expert feedback. The preamble also includes further background on the limitations of the review in response to expert review comments.

A separate section summarises further reflections from the expert feedback on research and progress in the sector.

Findings

Target group: Motivations. There is evidence of the costs of compliance being perceived as too high and street food vendors having high levels of knowledge but being unable to purchase basic hygienic materials, such as soap (Khairuzzaman et

al., 2014; Amaami et al., 2017; Haque and Kohda, 2020; Moges et al., 2024). Certification of informal vendors, and reputation for safe practices, has been reported to increase customers and revenue, however low socio-economic status is given as a barrier to achieving and maintaining the standards needed for certification (Haque and Kohda, 2020; Bannor et al., 2022). Economic motivations are a factor for compliance, if these barriers could be overcome (Adaku et al., 2024).

Informality was noted as contributing to the complexity and cost of compliance, as street food vendors were repeatedly displaced by government authorities, they were unable to find clean spaces to sell their products (Haque and Kohda, 2020; Moges et al., 2024).

Target group: Knowledge of the rules and capacity to comply. There is evidence that training and education are linked to higher levels of knowledge, and improved practices (Grace, 2015; Haque and Kohda, 2020; Azanza et al., 2000; Song, 2020; Oladipo-Adekeye et al., 2021; Bannor et al., 2022; Huynh-Van et al., 2022; Tuglo et al., 2023; Sepadi and Nkosi, 2023). However, this has been queried by others who find that appearances are more important than actual hygiene practices (Rheinländer et al., 2008) or found no link between education and practice (Nitin et al., 2025). In Brazil one study reporting high levels of compliance by street food vendors identified of education, qualifications and a stable financial position as being determining factors (Auad et al., 2018).

Making compliance easier is a strong theme, including simplifying regulations (Haque and Kohda, 2020; Moges et al., 2024) and providing access to sanitary infrastructure (Khairuzzaman et al., 2014; Habib, 2016; Azanza et al., 2000; Huynh-Van et al., 2022; Sepadi and Nkosi, 2023; Nitin et al., 2025).

Practitioner and academic articles recommend that regulations be adapted to the needs and capacity to comply of street vendors (FAO and WHO, 2003; Pulliat et al., 2024). Such adaption should be participatory (Martínez and Young, 2022). To be accepted by informal vendors, the formalization process needs to consider the factors which make them successful; the needed proximity to consumers, spatial mobility and operational flexibility (Huang et al., 2019). Wider interventions to support the socio-economic status of street food vendors and reduce poverty levels were recommended (Haque and Kohda, 2020; Azanza et al., 2000).

Perceptions of, and relationship with, the regulator. Distrust between vendors and regulators, typically local authorities, is identified as a common challenge (FAO, 2007; Forkuor, et al., 2017; Moges et al., 2024; Pulliat et al., 2024; Adaku et al., 2024).

The need for clear mandates and credible sanctions is identified (Dwumfour 2015; Alimo et al., 2016). Several papers identified the negative impacts of heavy-handed interventions and exclusionary practices in increasing conflict and non-compliance, in addition to excluding the most vulnerable vendors and increasing poverty (Haque and Kohda, 2020; Agyemang and Schoeman, 2022; Pulliat et al., 2024).

The failure of repressive policies against socially embedded and economically relevant informal economic practice are documented in Europe, Latin America, Africa and Asia. Attempts were made to displace or eradicate informal street vendors, before eventually moving to an inclusive formalisation process (Polese et al., 2016; Huang et al., 2019; Tafti, 2019; Contreras et al., 2020; Pulliat et al., 2024). Inclusive processes have included decriminalising street vendors and providing spaces for vending, with access to needed sanitary infrastructure (Von Holy and Makhoane, 2006; Polese et al., 2016; Tafti, 2019; Song, 2020).

In some cases, dedicated areas have been provided, with toilets and handwashing facilities, running water, garbage collection and public cleaning, with vendors being provided with training and regular inspections (Von Holy and Makhoane, 2006). This approach reduces the risk from solid waste management and poor road safety associated with unregulated street food vending, it can also make compliance easier and enables co-regulation. However, impacts on compliance have been uneven, as the approach does not allow for the needed mobility and flexibility of vendors (Von Holy and Makhoane, 2006).

A number of papers provided evidence to support to the bottom-up approach of regulating the informal economy through collaborative processes between street vendors and the state (Malasan, 2019; Abdullah et al., 2020; Recio, 2021; Agyemang and Schoeman, 2022; George et al., 2023). The most prominent model is the formation of associations, building upon existing strong informal networks of support and co-regulations between street vendors (Malasan, 2019; Abdullah et al., 2020; Recio, 2021). These associations can have positive impacts on trust between

vendors, and trust with the state, supporting more resilience (Kusakabe, 2006). However, they need to be empowered and supported (George et al., 2023; Tafti, 2019). A less explicit version is “managed informality,” where the local authorities actively and purposefully organize legal grey zones; where hierarchies of informal street vendors effectively provide co-regulation (Batréau and Bonnet, 2016; Tafti, 2019; Song, 2020). The regulators here are adapting to a situation where trust is low, and resources are limited.

This zoning or enabled co-regulation may exacerbate the inequalities among street vendors in accessing public spaces unless it considers power relations between street vendors (Batréau and Bonnet, 2016; Tafti, 2019; Song, 2020). Low-end or more vulnerable street vendors may be constrained or even exploited by better resourced vendors. Alejo and Schoenecker (2018) warn against conditions of formalisation which serve to further constrain and exclude the most vulnerable informal workers. Similarly, Meneses-Reyes (2018) highlights how licensing is the preferred means of street food vendor regulation across Latin America, as it provides autonomy to local municipalities and is in theory neutral, however unlicensed vendors tend to be the most vulnerable and can become more likely to be harassed.

Resourcing and supporting regulators. Poorly resourced regulators, and the lack of enforcement capacity was noted as limiting the impact of inspections on practice (Dwumfour-Asare, 2015; Amaami et al., 2017). Coping strategies adopted by regulators to perform their responsibilities within limited available resources can then create tensions and distrust between regulators and vendors (Forkuor et al., 2017). Inadequate time for local officials to understand and apply formalisation policies was a block to implementation in some locations (George et al., 2023).

Enabling environment. Fragmented governance is noted as a challenge; an integrated or harmonised system may be more effective (Monney et al., 2014; Grace, 2015). Experience has shown policies and regulations are rarely translated into practice (Grace, 2015).

Reflections from Expert feedback (Four No. Responses)

Expert reviewers emphasised some of the points covered during the review. Costs are typically not measured but re often considered to be high relative to benefits. Training is an important basis for building compliance. Regulatory authorities should implement training and certification programmes, alongside legal recognition.

Expert reviewers highlighted the potential for information-based regulation, with routine monitoring and testing, alongside public awareness campaigns.

The profile of street vendors themselves also needs to be considered when looking at vulnerability, for example, many vendors are female and vulnerable to gender based violence.

Re-locating vendors was noted not to have had much success expect in countries with strong governance and resources such as Singapore and Hong Kong. However, providing better infrastructure at common street vending points was supported, including water supply and waste management.

References

Abdullah, A., Bentum, H., Dwumah, P., Gyasi-Boadu, N., & Musah, M. N. (2020). Informal control and safety measures of street hawkers: narratives from costermongers. *Urban Forum* 31(4), 515-531.

Adaku, A. A., Egyir, I. S., Gadegbeku, C., Kunadu, A. P. H., Amanor-Boadu, V., & Laar, A. (2024). Barriers to ensuring and sustaining street food safety in a developing economy. *Heliyon*, *10*(11).

Agyemang, F. & Schoeman, I. (2022). Practical Demonstration of Street Trading Regulatory Management Practices. *International Journal of Management Research and Economics*. 2.

Alejo, E., & Schoenecker, A. (2018). Policies of exclusion: Space, time, and the informal economy in Chicago. *Space and Culture*, 23(4), 477-491.

Alimi, B. A. (2016). Risk factors in street food practices in developing countries: A review. *Food science and human wellness*, *5*(3), 141-148.

Amaami, A. J., Dominic, D., & Collins, D. (2017). Factors associated with poor food safety compliance among street food vendors in the Techiman Municipality of Ghana. *African Journal of Food Science*, 11(3), 50-57.

Auad, L. I., Ginani, V. C., dos Santos Leandro, E., Nunes, A. C. S., Domingues Junior, L. R. P., & Zandonadi, R. P. (2018). Who is serving us? Food safety rules compliance among Brazilian food truck vendors. *International Journal of Environmental Research and Public Health*, *15*(12), 2807.

Azanza, P. V., Gatchalian, C. F., Ortega, M.P. (2000). Food safety knowledge and practices of street food vendors in a Philippines university campus. *International journal of food sciences and nutrition*, 51(4), 235-246.

Bannor, R. K., Amfo, B., & Oppong-Kyeremeh, H. (2022). Examining the nexus between food retailers certification and its impacts. *International Journal of Retail & Distribution Management*, 50(10), 1238-1258.

Batréau, Q., & Bonnet, F. (2016). Managed informality: Regulating street vendors in Bangkok. *City & Community*, 15(1), 29-43.

Contreras, C. P. A., Cardoso, R. D. C. V., da Silva, L. N. N., & Cuello, R. E. G. (2020). Street food, food safety, and regulation: what is the panorama in Colombia?: a review. *Journal of Food Protection,* 83(8), 1345-1358.

Dwumfour-Asare, B. (2015). Effect of local authorities' field monitoring visits on awareness of regulation and hygiene practices among street food vendors: the case of two district capitals in Ghana. *Journal of Behavioral Health*, 4(3), 71-80.

FAO (2007). *Promises and Challenges of the Informal Food Sector in Developing Countries*. Food and Agricultural Organization. Rome.

FAO & WHO (2003). Assuring food safety and quality: guidelines for strengthening national food control systems. *FAO Food and Nutrition Paper, 76*. Food and Agricultural Organization. Rome.

Forkuor, J. B., Samuelsen, H., Yeboah, E. H., Rheinländer, T., & Akuoko, K. O. (2017). The regulation of street foods: Experiences of front-line regulators in Ghana. *Urban Forum.* 28, 251–269.

Grace, D. (2015). Food safety in low- and middle-income countries. *International journal of environmental research and public health*, 12(9), 10490-10507.

George, C., Msoka, C. T., & Makundi, H. (2023). Formalisation of street vending in Dar es Salaam: Implementation and enforcement of the Wamachinga identity card initiative. *Forum for Development Studies,* 50(2), 283-302.

Habib, K. R. (2016). Understanding Challenges Faced by Street Food Vendors to Maintain Street Food Hygiene in Dhaka City. *Journal of Food and Nutrition Sciences,* 4, 78-85.

Haque, I. T., & Kohda, Y. (2020). Understanding the impact of social determinants of health in street food safety: a qualitative study in Bangladesh. *International Journal of Health Promotion and Education*, 58(3), 152-162.

Huang, G., Xue, D., & Wang, Y. (2019). Governmentality and spatial strategies: Towards formalization of street vendors in Guangzhou, China. *International Journal of Urban and Regional Research*, 43(3), 442-459.

Huynh-Van, B., Vuong-Thao, V., Huynh-Thi-Thanh, T., Dang-Xuan, S., Huynh-Van, T., Tran-To, L., ... & Nguyen-Viet, H. (2022). Factors associated with food safety compliance among street food vendors in Can Tho city, Vietnam: Implications for intervention activity design and implementation. *BMC Public Health*, 22, 1-11.

Khairuzzaman, M. D., Chowdhury, F. M., Zaman, S., Al Mamun, A., & Bari, M. L. (2014). Food safety challenges towards safe, healthy, and nutritious street foods in Bangladesh. *International journal of food science*, 2014(1), 483519.

Kusakabe, K. (2006). *Policy issues on street vending: an overview of studies in Thailand, Cambodia and Mongolia. Informal economy, poverty and employment.* Bangkok, International Labour Organisation.

Malasan, P. L. (2019). The untold flavour of street food: Social infrastructure as a means of everyday politics for street vendors in Bandung, Indonesia. *Asia Pacific Viewpoint*, 60(1), 51-64.

Martínez, L., & Young, G. (2022). Street vending, vulnerability and exclusion during the COVID-19 pandemic: the case of Cali, Colombia. *Environment and Urbanization*, *34*(2), 372-390.

Meneses-Reyes, R. (2018). (Un) authorized: A study on the regulation of street vending in Latin America. *Law & Policy*, 40(3), 286-315.

Moges, M., Rodland, E. K., & Argaw, A. (2024). Ethiopian Street Foods: Working Conditions and Governance Perspectives. A Qualitative Study. *Environmental Health Insights*, 18, 11786302241241414.

Monney, I., Agyei, D., Badzi, S. E., Campaore, P., & Nyaw, S. (2014). Food Hygiene and Safety Practices among Street Food Vendors: An Assessment of Compliance, Institutional and Legislative Framework in Ghana. *Food and Public Health*, 4, 306-315.

Nitin, K., Puja, D., Sanjay, C., Sunil, T., Poonam, K., Poojan, M., & Sushruti, K. (2025). A Cross-Sectional Study to Assess Street Food Vendors' Adherence to the Bureau of Indian Standards (BIS) in an Urban Slum of Pune, India. *Cureus*, *17*(3).

Oladipo-Adekeye, O. T., & Tabit, F. T. (2021). The food safety knowledge of street food vendors and the sanitary compliance of their vending facilities, Johannesburg, South Africa. *Journal of Food Safety*, 41(4), e12908.

Polese, A., Rekhviashvili, L., & Morris, J. (2016). Informal governance in urban spaces: Power, negotiation and resistance among Georgian street vendors. *Geography Research Forum*, 36(2), 15-32.

Pulliat, G., Block, D., Bruckert, M., Nussbaum-Barberena, L., Dreysse, C., Dupé, P., & Perrin, C. (2024). Governing the nurturing city: the uneven enforcement of street food vending regulations. *Urban Geography*, 45(7), 1148-1172.

Recio, R. B. (2021). How can street routines inform state regulation? Learning from informal traders in Baclaran, Metro Manila. *International Development Planning Review*, 43(1), 63-88.

Rheinländer, T., Olsen, M., Bakang, J. A., Takyi, H., Konradsen, F., & Samuelsen, H. (2008). Keeping up appearances: perceptions of street food safety in urban Kumasi, Ghana. *Journal of Urban Health*, 85, 952-964.

Sepadi, M. M., & Nkosi, V. (2023). Strengthening urban informal trading and improving the health of vendors: An integrated management model. *International journal of environmental research and public health*, *20*(6), 4836.

Song, S. (2020). Street stall economy in China in the post-COVID-19 era: Dilemmas and regulatory suggestions. *Research in Globalization*, 2, 100030.

Tafti, M. T. (2019). Assembling street vending. *Urban Studies*, 57(9), 1887-1902.

Tuglo, L. S., Mishra, S., Mohapatra, R. K., Kortei, N. K., Nsor-Atindana, J., Mulugeta, H., ... & Doku, A. (2023). A systematic review and meta-analysis of food handling practices in Ghana vis-a-vis the associated factors among food handlers during 2009 and 2022. *Scientific Reports*, 13(1), 18748.

Von Holy, A., & Makhoane, F. M. (2006). Improving street food vending in South Africa: Achievements and lessons learned. *International journal of food microbiology*, 111(2), 89-92.

S.3-2: Construction waste management (illegal dumping).

Literature identified

Sources and steps are given in the PRISMA diagram below. The search string used was: [[construction AND waste AND [management OR disposal]] AND [Regulation OR Compliance] AND [impact OR evidence OR change OR increase]. Compliance is considered around legal dumping. Other forms of compliance, such as reduction of waste or incentivizing recycling were excluded as being less relevant.

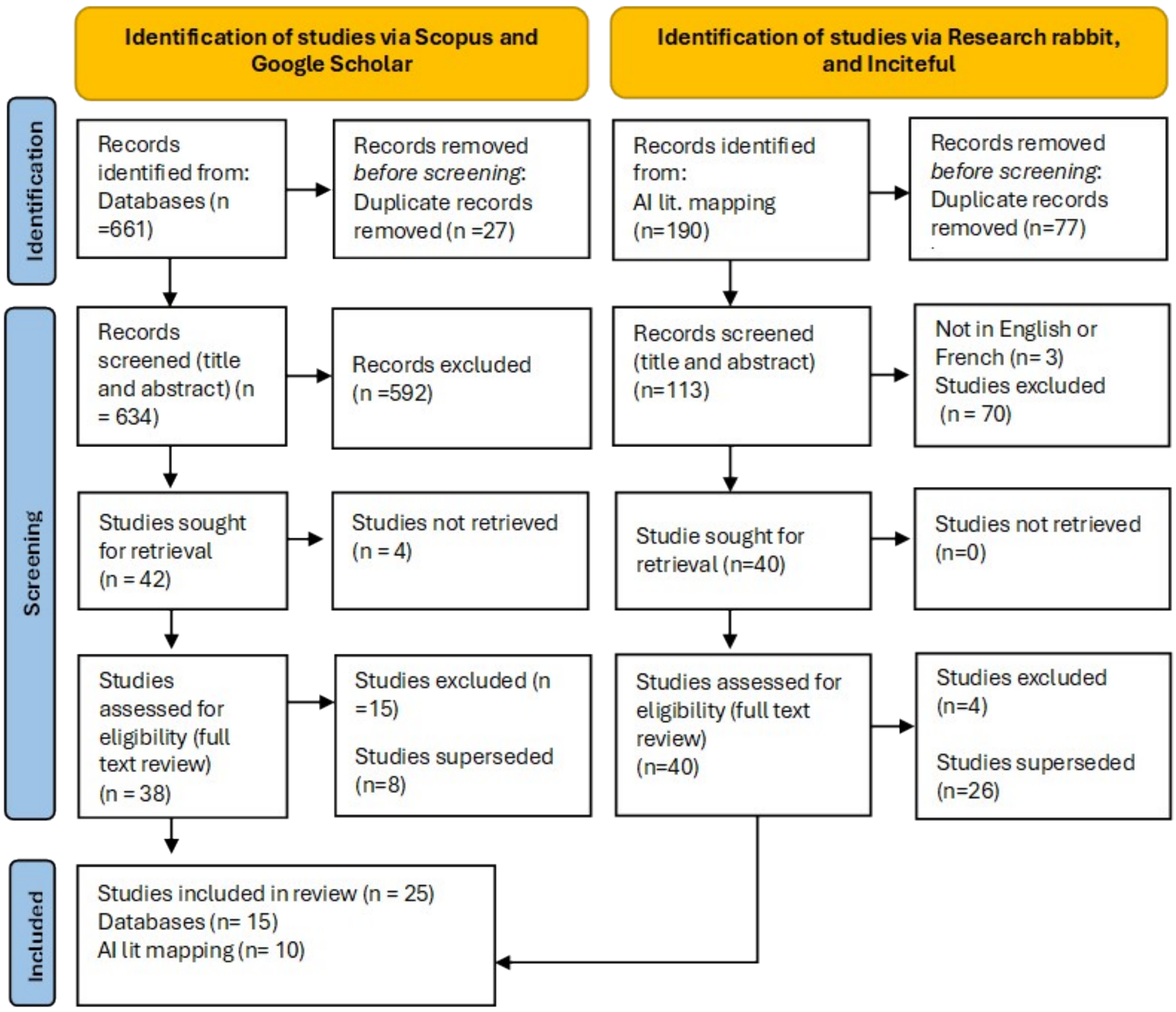

The construction industry addressed its illegal waste disposal problems by investing in: (i) minimization of waste by prefabrication and modular construction (ii) circularity, reuse, and resource recovery. For new construction sites the waste is largely limited to packaging materials. Remaining issues of illegal dumping in the construction sector are primarily at demolition sites. This rapid review focusses on directly reducing illegal dumping as this is the closest to the analogous sector under consideration – faecal sludge emptying and transport.

The rapid review findings incorporate 5 of the additional 6 recommended references from Expert feedback, with one duplicate removed. These are related to the design of environmental regulation to use market drivers and are included in a separate sub-section as they are also relevant to the parallel review on municipal solid waste management. The preamble also includes further background on the limitations of the review in response to these comments.

A separate section summarises further reflections from the expert feedback on research and progress in the sector.

Findings

Target group: Motivations. Motivations for illegal dumping are typically considered from an economic perspective (Lu, 2019; Du et al., 2021). Illegal dumping is the cheapest option, avoiding high transport and costs charged for disposal or recycling (Lu, 2019; Du et al., 2021). It is often also the quickest option, queues were identified as a major disincentive to safe disposal, linking back to saving time (Lu, 2019). Costs of compliance may be perceived to outweigh the benefits (Swarna Swetha et al., 2022). High costs of disposal at landfill can also drive positive behaviours, such as recycling (Du et al., 2021). A number of papers recommended investing in resale of recycled products, or compensation for recycling, to reduce illegal dumping, rather than the threat of enforcement (Kim et al., 2008; Ramos et al., 2023ab).

Several papers focused on information-based regulation seem to assume a game playing attitude to compliance, with the only interest being on calculated risk – see the section on **relationship with the regulator**. However, many studies have found that both individual stakeholders' willingness and attitude to illegal dumping, and also the perceived value of compliance by colleagues and prospective clients were highly influential in changing behaviour (Adjei et al., 2015; Du et al., 2021; Swarna Swetha et al., 2022; Yuan et al., 2023). Yu et al., (2023) highlight the social importance of pro-environmental behaviour linked to recycling. In some cases, illegal dumping was considered a positive behaviour as it facilitated informal reuse of goods (Du et al., 2021). A positive attitude towards the policy and agreement with policy aims can contribute to compliance (Du et al., 2021).

Target group: Knowledge of the rules and Capacity to comply. Knowledge and understanding of requirements, and the impacts of non-compliance were identified as significant factors contributing to construction waste management (Seror and Portnov, 2018; Swarna Swetha et al., 2022; Ramos et al., 2023ab). Ramos et al. (2023ab) note the importance of adopting approaches for local implementation by small scale organizations, who face specific challenges regarding the lack of knowledge and capacity. They recommend oral communication strategies to teach waste producers both how and why they should use dump sites. Ramos et al. (2023 ab) highlight that cooperative approaches should be used, working with waste producers in dealing with logistical challenges and delivering training.

The impact of the availability of local dump sites on illegal dumping is contested. Establishing more proximate dump sites is commonly believed to be a major contributor to reduce illegal dumping by making compliance easier and cheaper (Du et al., 2021; Ramos et al., 2023ab; Ichinose and Yamamoto, 2011). Ramos et al., (2023c) demonstrated that providing local intermediate disposal solutions to individuals or small businesses significantly reduced illegal disposal. However, Lu et al. (2025) find that this is not a determining factor, and that wider urban planning may be more influential – see **Enabling environment** below. They describe dump sites as acting only as a 'local modifier' of dumping behaviour.

Perceptions of, and relationship with, the regulator. The literature did not appear to discuss building the relationship between waste producers and regulators. The two main areas of focus were the design of sanctions and supervision, and the use of information-based regulation.

Supervision and surveillance are widely considered to reduce the risk of illegal dumping. The impact is contested by more recent research (Lu et al., 2025) however it remains a major focus in the literature. Good practice includes the use of fixed-point monitoring at known, or expected, hot spots for illegal dumping, combined with patrol supervision (Seror and Portnov, 2018; Du et al., 2021; Fang et al., 2023; Barakat and Srour, 2024). The balance between fixed point monitoring, which is cheaper, but easily avoided, and more resource intensive patrols is debated. Liu et al. (2022) used evolutionary game theory to model required supervision intensity for

various scenarios of income (or cost averted) from illegal dumping and the cost of fines.

Satellite imagery, big data and modelling are increasingly used to target supervision by identifying likely illegal disposal sites, including marginalised areas, and suspicious behaviour (Lu, 2019; Du et al.2021; Fang et al., 2023; Bao et al., 2024; Iacoboaea et al., 2024; Ray et al, 2024). For registered vehicles these measures could be combined with video recognition and GPS tracking of suspect operators (Lu, 2019). These methods cannot be used on unregistered vehicles or in informal areas, where volumes dumped may be small (Lu, 2019; Fang et al., 2023).

Research into the design of sanctions identifies that they need to immediate and relevant. Seror and Portnov (2020) identified that immediate vehicle impoundment is an effective deterrent. A number of studies identified that criminal proceedings are not effective, potentially due to the delay between the action and the sanction being applied (Seror and Portnov, 2020; Germani et al., 2024).

Increasing penalty fines were found to have an impact only when there is an associated increase in supervision, or other action taken to increase the probability of being detected and punished (Seror and Portnov, 2020; Liu et al., 2022; Germani et al., 2024). Dixon et al. (2022) identify the importance of perceived risk in decision making; changes to increase the probability of detection need to be made visible and known. Excessive penalty rates may increase the risk of illegal dumping, by allowing a greater margin for payoffs of officials thereby incentivizing corruption (Abatemarco et al., 2024). Yang et al. (2021) identify that punitive regulation should be used only when recycling is very costly and illegal dumping is highly damaging. Similarly, Jia et al. (2017) found that penalties were effective when there were associated subsidies, or other financial incentives, making recycling or waste reduction feasible.

Enabling Environment. How cities are laid out makes a significant difference to dumping patterns. Illegal dumping is more likely to occur in low population density areas, but also lower per capita floor area (Lu et al., 2025). Street layouts, including accessibility and visibility can create opportunities or incentives for dumping illegally (Hohl et al., 2023; Lu et al., 2025). Similarly poor condition of roads leading to a legal dumping site can increase illegal dumping (Du et al., 2021): Outside of cities, similar conditions are created by forests or other conditions which make illegal dumping less

visible (Du et al., 2021). High risk locations could be identified based on these metrics, and mapping of known illegal dump sites, and enable preventative action (Du et al., 2021; Lu et al., 2025).

Multiple studies found that compliance is not primarily driven by legislation (Adjei et al., 2015; Yuan et al., 2023).

External actors. Public participation in managing illegal dumping may be one approach to reduce the cost of supervision and investigation (Du et al., 2021; Al Fariz et al, 2025). Government support would be needed to strengthen community management, ensure there are proximate legal sites and raise awareness of the harm caused by illegal dumping (Du et al., 2021). Yu et al. (2023) highlight the importance of both public and media supervision. Al Fariz et al. (2025) document an approach where local communities were successfully engaged to identify illegal disposal sites. Swarna Swetha et al. (2022) note that this approach requires a greater value being placed upon environmental protection and good waste management.

Du et al. (2021) note that in higher income areas communities act to reduce illegal dumping. Similarly, economic decline of a community is linked an increase in illegal dumping (Hohl et al., 2023). Wider economic development could be expected to have a positive impact on illegal dumping (Du et al., 2021). Per capita floor area may be a proxy for income level, linking to the findings around planning by Lu et al. (2025).

Regulatory design: Economic incentives and enforcement. An influential idea in regulation of construction waste management is that "*well-designed environmental regulation can stimulate innovation and enhance competitiveness"* (Porter and van der Linde, 1995). Regulated organisations should be able to reduce their costs by complying with regulations and diminishing pollution. In construction waste management many companies have learnt *"to frame environmental improvement in terms of resource productivity, or the efficiency and effectiveness ... use resources".* This is reflected in the major changes to reduce construction waste by changing construction techniques and the incentives and charges to drive reuse and recycling

from demolition (Du et al., 2021; Ramos et al., 2023a). The findings of Huang et al. (2024) and Lavee (2020) reinforce the argument that economic tools can achieve policy objectives more effectively than command and control regulation, while driving the market toward environmental improvement.

Recommendations for regulatory design include the following; (i) clear goals and flexible approaches which focus on outcomes, not technologies; (ii) use of market incentives, including pollution taxes, deposit-refund schemes and tradable permits; (iii) Industry participation in regulation development and coordination of environmental regulation: between industry and regulators, between regulators at different levels and places in government (Porter and van der Linde, 1995):

Liu et al. (2022) demonstrate that stock markets may focus on costs, rather than opportunities, of environmental regulation. They identify that environmental policy roll-outs can send strong negative signals to markets, especially for firms perceived as unprepared, or closer links to government (Liu et al., 2022). This suggests that regulated organisations may need support through implementation of new regulation, even where these are expected to have a positive long-term impact on competitiveness.

For sectors such as solid waste management with significant externalities, the costs of compliance with regulation may still be high if those externalities are not costed. Mihajlović et al. (2016), researching compliance with EU waste management requirements in Serbia, identify a negative net present value for all investment options into waste management and a requirement for significant %GDP to maintain costs at affordable levels.

Reflections from Expert feedback (Three No. Responses)

Experts emphasised the importance of wider stakeholder cooperation, proximity of affordable and convenient disposal sites supported by training and supervision and company size. Smaller enterprises have more constraints than larger companies in accessing information and capacity building, and typically less oversight from the authorities.

Experts emphasised the importance of market mechanisms to drive a wide range of behaviour in construction waste management, reducing waste generation and

increasing recycling and reuse. The most substantial addition made was to include the foundational economic perspectives on environmental regulation. The idea that well-designed environmental regulation can stimulate innovation and enhance competitiveness has been instrumental in driving change in construction waste management. One expert highlighted that it is this principle which is likely to have the most cross sectoral relevance.

References

Abatemarco, A., Cascavilla, A., Dell'Anno, R., & Morone, A. (2024). Maximal fines and corruption: An experimental study on illegal waste disposal. *Waste Management*, 183, 153-162.

Adjei, S., Ankrah, N., Ndekugri, I., & Searle, D. (2015). Investigating the extent to which waste management legislation affects waste management practices within the UK construction industry. In *Proceedings 31st Annual ARCOM Conference*, 2015 September (pp. 7-9).

Al Fariz, R. D., Rachman, I., & Matsumoto, T. (2025). Uncovering illegal waste disposal sites: Engaging communities in investigative solutions. *Multidisciplinary Science Journal*, 7(2), 2025057-2025057.

Bao, Z., Li, S., Chen, Y., Xie, H., Long, W., & Chen, W. Q. (2024). Applications of geospatial technologies for construction and demolition waste management: A systematic literature review. *Journal of Industrial Ecology*.

Barakat, B., & Srour, I. (2024). Consideration of hotspots in the selection of supervision schemes to reduce illegal dumping of construction and demolition waste. *Waste Management & Research*, 42(2), 146-157.

Dixon, A. C., Farrell, G., & Tilley, N. (2022). Illegal waste fly-tipping in the Covid-19 pandemic: enhanced compliance, temporal displacement, and urban–rural variation. *Crime Science*, 11(1), 8.

Du, L., Xu, H., & Zuo, J. (2021). Status quo of illegal dumping research: Way forward. *Journal of Environmental Management*, 290, 112601.

Fang, B., Yu, J., Chen, Z., Osman, A. I., Farghali, M., Ihara, I., ... & Yap, P. S. (2023). Artificial intelligence for waste management in smart cities: a review. *Environmental Chemistry Letters*, 21(4), 1959-1989.

Germani, A. R., Castaldo, A., & Ker, A. (2024). Carrots, sticks, and environmental crime in Italy. *European Journal of Law and Economics*, 58(2), 201-219.

Hohl, B. C., Kondo, M. C., Rupp, L. A., Sadler, R. C., Gong, C. H., Le, K., ... & Zimmerman, M. A. (2023). Community identified characteristics related to illegal dumping; a mixed methods study to inform prevention. Journal of Environmental Management, 346, 118930.

Huang, L., Zhou, X., Chi, L., Meng, H., Chen, G., Shen, C., & Wu, J. (2024). Stimulating innovation or enhancing productivity? The impact of environmental regulations on agricultural green growth. *Journal of Environmental Management*, 370, 122706.

Ichinose, D., & Yamamoto, M. (2011). On the relationship between the provision of waste management service and illegal dumping. *Resource and energy economics*, 33(1), 79-93.

Kim, G. S., Chang, Y. J., & Kelleher, D. (2008). Unit pricing of municipal solid waste and illegal dumping: An empirical analysis of Korean experience. *Environmental Economics and Policy Studies*, 9, 167-176.

Iacoboaea, C., Luca, O., Șercăianu, M., Aldea, M., Păunescu, M., & Popescu, A. L. (2024). Towards Sustainable Modes for Remote Monitoring in Waste Management: A Study of Marginalized Urban Areas in Romania. *Sustainability*, 16(6), 2400.

Jia, S., Yan, G., Shen, A., & Zheng, J. (2017). Dynamic simulation analysis of a construction and demolition waste management model under penalty and subsidy mechanisms. *Journal of cleaner production*, 147, 531-545.

Lavee, D. (2020). Are economic tools preferable to direct regulatory measures in achieving environmental goals?. *Environmental Policy and Law*, 50(3), 181-191.

Liu, C., Hua, C., & Chen, J. (2022). Efficient supervision strategy for illegal dumping of construction and demolition waste: A networked game theory decision-making model. *Waste Management & Research*, 40(6), 754-764.

Lu, W. (2019). Big data analytics to identify illegal construction waste dumping: A Hong Kong study. *Resources, conservation and recycling*, 141, 264-272.

Lu, W., Yang, B., Yuan, L., & Peng, Z. (2025). Understanding fly-tipping in urban areas: A social-economic-spatial combinatorial approach enabled by geographically weighted random forest. *Environmental Impact Assessment Review*, 112, 107858.

Mihajlović, V., Vujić, G., Stanisavljević, N., & Batinić, B. (2016). Financial implications of compliance with EU waste management goals: Feasibility and consequences in a transition country. *Waste Management & Research*, 34(9), 923-932.

Porter, M. E., & Linde, C. V. D. (1995). Toward a new conception of the environment-competitiveness relationship. *Journal of economic perspectives*, 9(4), 97-118.

Ramos, M., Martinho, G., & Pina, J. (2023a). Strategies to promote construction and demolition waste management in the context of local dynamics. *Waste Management*, 162, 102-112.

Ramos, M., Martinho, G., Vasconcelos, L., & Ferreira, F. (2023b). Local scale dynamics to promote the sustainable management of construction and demolition waste. *Resources, Conservation & Recycling Advances*, 17, 200135.

Ramos, M., & Martinho, G. (2023c). An assessment of the illegal dumping of construction and demolition waste. *Cleaner Waste Systems*, *4*, 100073.

Ray, S., Ng, K. T. W., Mahmud, T. S., Richter, A., & Naghibalsadati, F. (2024). Quantification of construction and demolition waste disposal behaviors during COVID-19 using satellite imagery. *Environmental and Sustainability Indicators*, 24, 100502.

Seror, N., & Portnov, B. A. (2020). Estimating the effectiveness of different environmental law enforcement policies on illegal C&D waste dumping in Israel. *Waste Management*, 102, 241-248.

Swarna Swetha, K., Tezeswi, T. P., & Siva Kumar, M. V. N. (2022). Implementing construction waste management in India: An extended theory of planned behaviour approach. *Environ. Technol. Innov*, 27(1866), 102401.

Yang, J., Zhang, J., Guo, C., Tan, R., & Yu, M. (2021). Incentive or punitive measure? Analysis of environmental regulations in construction and demolition waste recycling. *Mathematical Problems in Engineering*, 2021(1), 6619980.

Yu, S., Hao, J. L., Di Sarno, L., Ma, W., Guo, N., & Liu, Y. (2023). Enhancing pro-environmental behaviour of employees towards renovation waste for a circular economy: The role of external supervision and corporate environmental responsibility. *Sustainable Chemistry and Pharmacy*, 33, 101103.

Yuan, H., Du, W., Ma, X., Liu, J., & Li, L. (2023). Critical factors to influence the illegal dumping behavior of construction and demolition waste: An ISM-DEMATEL analysis. *Developments in the Built Environment*, 14, 100159.

S.3-3: Artisanal mining

Literature identified

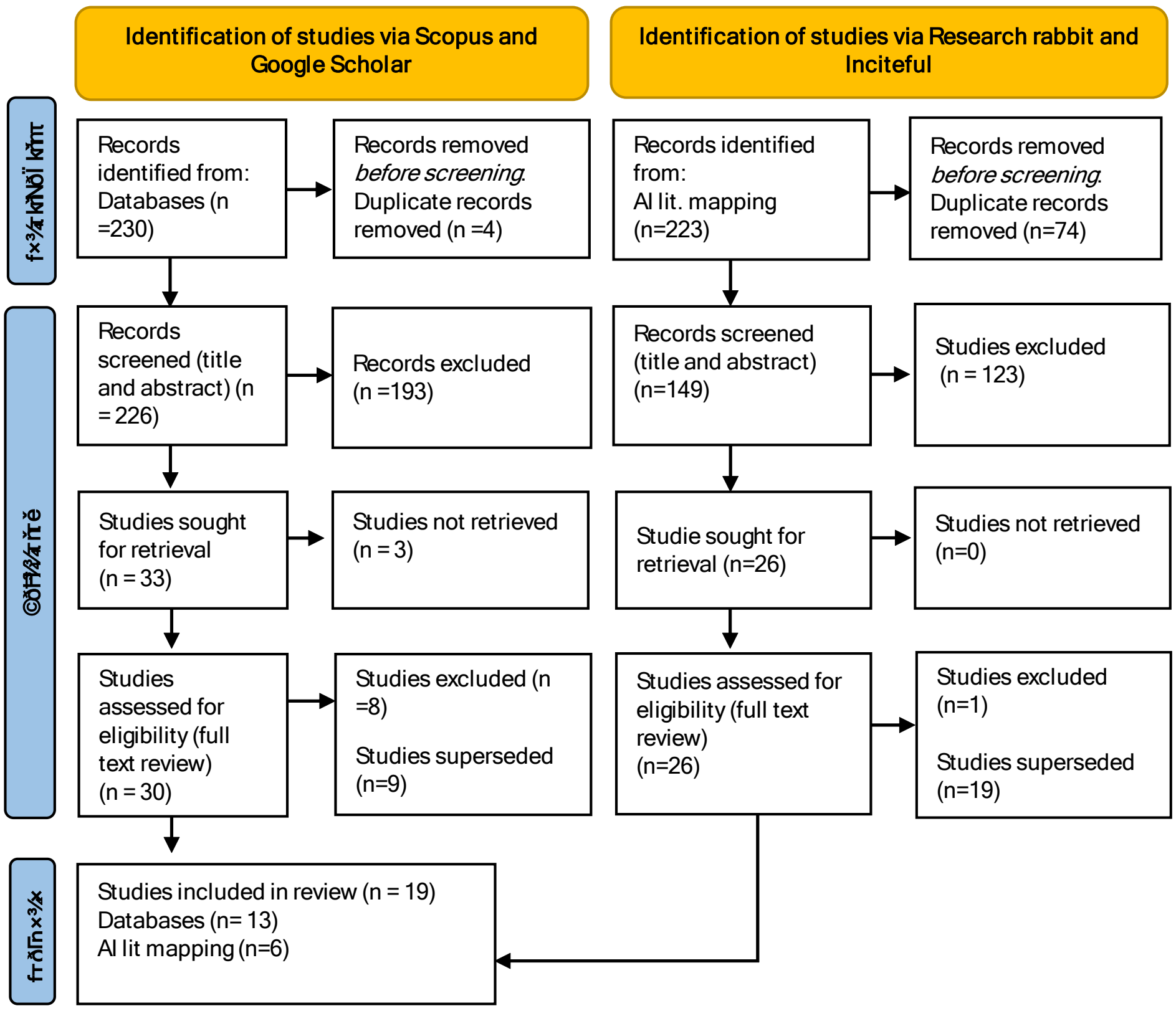

Sources and steps are given in the PRISMA diagram. The search string used was: [Artisanal OR small scale OR illegal] AND [mining] AND [Regulation OR Compliance] AND [impact OR evidence OR change OR increase].

The majority of artisanal and small-scale miners (ASM), worldwide, operate informally and considerations of compliance and formalisation are intertwined (Hilson and Maconachie, 2017). The literature identified focussed on the development and formalisation of ASM (Arthur-Holmes and Ofosu, 2024). Formalisation in ASM can be defined as "*the process through which miners transition to working within the state's regulatory framework*" (Velez et al., 2025). This process can be limited to licensing but should also incorporate technical and organisational support (Velez et al., 2025). In this review we followed the majority of academics who define it widely as being able to work within the law and have safe social and environmental working

practices (Greenen, 2012; Siegel and Veiga, 2009). Social issues consider ASM as contributing to poverty alleviation and livelihoods development (Spiegel et al., 2015).

In general, the literature is case study based. The most recent article was taken for each country studied. In locations where there was a significant body of literature, such as Ghana and Peru, a number of recent case studies were taken to check alignment. The literature identified was largely skewed towards gold mining, this was identified in the expert review and additional literature has been included to counteract this point.

There have been no long-term, well-documented cases of formalization success. The unusual case of Guyana, where the development of "*indigenous small- and medium-scale operators*" has been prioritised, was initially celebrated but later found to face many similar challenges in terms of elite capture of resources and limited environmental protection (Hilson and Maconachie, 2017; Hilson and Laing, 2019; Hook, 2019)

The documented or proposed good practice appeared, to a non-sector specialist, to be highly consistent across locations. However, it is recognised that there are huge levels of variation in regulations, and building compliance, within and between the countries covered in this review. In each country there are different definitions of formalisation and requirements for formalisation. There are also important differences between the minerals or metals being mined. This review tries to take only those issues which are common across multiple locations and which could be considered to be more widely generalisable. However, this flattening out of much of the variation is recognised as a limitation of the review.

The rapid review findings incorporate 15 of the additional 18 recommended references from Expert feedback, with three duplicates removed. The preamble also includes further background on the limitations of the review in response to expert review comments. A separate section summarises further reflections from the expert feedback on research and progress in the sector.

Findings

Target group: Motivations. Miners have stated their willingness to formalise with the right assistance and material, technical and financial support (Greenen, 2012).

However, in practice, the costs of formalization policies in ASM have outweighed the benefits (Greenen, 2012; Mutemeri et al., 2016; Arthur-Holmes and Ofosu, 2024; Velez et al., 2025). Costs included both the bureaucratic barriers to obtaining legal licenses, including travel for long distances, long waiting times and high costs to obtaining the correct documentation, and the technical costs to comply with working practices. Benefits from formalisation, apart from the avoidance of punitive responses, are often not clear (Speigel et al., 2015).

Jiménez et al. (2024) found that people willing to take higher risks or who needed an immediate income may be more likely to choose informal mining. In contrast, they found that people with strong family ties, who were risk-averse and who placed a high value on the security offered by formal mining, would choose to work for formalized ASM companies. Jiménez et al. (2024) highlight the importance of understanding motivation to formalise and how these may vary by individual and over time; miners moved back and forth between formal and informal practices dependant on personal drivers, for example the need to cover emergency medical fees for family.

Target group: Knowledge of the rules and capacity to comply. Historically the problems relating to ASM have been addressed through the bureaucratic process of licenses and registration. A number of cases identified that the focus should be on improved practice, rather than bureaucracy, in order to have an impact (Greenen, 2012; Martinez et al., 2023). A top-down technocratic approach to improving practices has had limited impact (Andriamasinoro et al., 2020; Mutemeri et al., 2016). Understanding of the formalisation process and its implications may be weak (Velez et al., 2025). However, knowledge of the rules alone was found to have little effect, change required both top-down policy change and bottom-up support and adaption (Martinez et al., 2023).

Capacity to comply was a significant barrier across locations. The majority of formalisation attempts required a costly licensing process and for miners to shift away from traditional mining practices (Martinez et al., 2023; Hilson and Maconachie, 2017; Siegel and Veiga, 2009). Some requirements were not feasible in practice, for example being able to legally purchase explosives (Jiménez et al., 2024) or being required to use equipment which was not suitable to the ore being

mined (Arthur-Holmes and Ofosu, 2024). Mercury removal is a common requirement for formalisation in gold mining, but there are no documented cases of viable alternatives to mercury, or complete mercury elimination, thereby blocking formalisation (Veiga and Fadina,2020; Keane et al., 2023). Criminalisation of practices, where there are no viable and affordable alternative can have negative social and environmental impacts as miners turn to working at night or in less accessible locations, increasing the risk from poor practices (Spiegel et al., 2015; Mutemeri et al., 2016; Siwale and Siwale,2017).

Adapting formalisation processes to capacity, and building capacity to meet requirements, were identified as parallel and interconnected responses. The formalisation process needed to be highly flexible, developed through bottom-up participatory approaches, adapted to the location and the individual needs (Arthur-Holmes and Ofosu, 2024). Continuous support through training, provision of equipment and machinery, and business development was required to enable formalisation and prevent miners reverting to unsafe practices (Siegel and Veiga, 2009; Siwale and Siwale, 2017; Martinez et al., 2023). Capacity building was also needed to enable local stakeholders to participate in policy formulation and implementation (Mutemeri et al., 2016). Financial mechanisms such as microfinance, mobilizing savings and facilitating access to credit have been demonstrated to enable improved environmental practices, but only within communities where miners were already licensed (Spiegel et al., 2015). Given the location of ASM within low-income rural communities, wider socio-economic development is also needed to enable compliance (Spiegel et al., 2015; Martinez et al, 2023).

The majority of the literature above has focussed on gold mining. Legalisation of artisanal mining of copper in Chile has been largely successful, in terms of mining rights and tenure, and steps have been taken in wider formalisation, including skills transfer, equipment and access to geological data (Atienza et al., 2023). In this case the regulator also provides a technical support role and is the main buyer and processor of ore – meaning they can stabilise prices. However, even this well documented success story has its limitations due to the limited budget for equipment and the challenges of supporting competition while protecting vulnerable miners. (Atienza et al., 2023).

Perceptions of, and relationship with, the regulator. Over the last 20 years there has been a shift in the perceptions of ASM by many governments; with formalisation, rather than exclusion, now accepted by some as a first step (Martinez et al., 2023: Siegel and Veiga, 2009; Geenen, 2012; Bansah, 2023). However heavy-handed military or police responses to control ASM have been attempted in multiple locations and there were often many years of violent oppression before governments were forced to adopt an alternative approach (Bansah, 2023; Ayambire et al., 2024; Spiegel et al., 2015; Greenen, 2012). Recent papers on gold-mining communities in Ghana identify how this deterrence approach has continued to be popular, enabling the government to facilitate access for large scale mining companies, demonstrate action to the international community and also provide "a*n avenue where some state officials look for wealth creation opportunities*" (Ofari et al., 2021).

Studies indicated that this heavy-handed response weakened trust between regulators and low-income mining communities (Ayambire et al., 2024; Spiegel et al., 2015; Greenen, 2012). In some cases, these responses were credited with fuelling defiance and actively increasing non-compliance (Ayambire et al., 2024; Spiegel et al., 2015).

In terms of implementation, gradual stringency was proposed as one approach, where demands are stepped up in line with capacity and rewards or benefits (Rodríguez et al.,2021). Spiegel et al., 2015, recommend prioritizing local knowledge in technology decision making and capacity building, in terms of both understanding the local context and adapting implementation to local technological capabilities. Trust, between the government and local miners is identified as an important factor which shapes perceptions of regulations, high levels of distrust are likely to result in regulations being perceived as less fair, particularly in terms of access to mining areas (Heemskerk et al., 2015). There is a need for both sides to invest in relationship building and developing trusted relationships as part of the formalisation process (Heemskerk et al., 2015; Jiménez et al., 2024).

In many cases Governments may not want to support the formalization of ASM. There may be a significant bias towards facilitating large scale mining, driven by the desire for foreign investment and perceived economic benefits (Hilson, 2019; Veiga

and Fadina 2020). Hilson (2019) highlights this specifically in sub-Saharan Africa, where investment into large scale mining has occurred at the expense of ASM, and not recognised the potential of ASM to contribute to the economy. This prioritisation towards large scale mining has seen larger mining companies benefits from access to land, resources, tax incentives, and supportive policies, to the detriment, or at best benign neglect, of ASM (Hilson, 2019). Experience in Zambia illustrated how the government can embrace the ASM formalisation challenge when there are potential financial gains (Hilson, 2020). Although not successful in the long term this example illustrates how taxation can be a driver for formalisation and support both operators and government (Hilson, 2020).

Resourcing and supporting regulators. The resources required to monitor ASM, it's dispersed nature and chronic underfunding of regulators are highlighted (Marshall et al., 2022). Formalisation requires a multi-stakeholder approach – including government and non-governmental actors (Martinez et al., 2023; Mutemeri et al., 2016). Information based regulation, such as using satellite imagery to detect unauthorised mining activity have been identified as one approach to reduce the cost of regulation (Marshall et al, 2022).

Enabling Environment. Siegel and Veiga (2009) highlight that formalization of ASM should be viewed as '*a process, not a product*' and should incorporate customary practices. The legal system for ASM often arrives only after customary practices and informal social arrangements are established. Formal legal systems need to take account this existing enabling environment (Siegel and Veiga, 2009; Greenen, 2012; Mutemeri et al., 2016; Huntington and Marple-Cantrell, 2021) and strengthen existing local organisations that assist with regulating ASM activities (Mutemeri et al., 2016). Aligning informal and formal systems, which may face deep cultural and structural divides, requires an understanding of customary institutions and existing dependencies, reciprocal relations and power structures (Huntington and Marple-Cantrell, 2021; Siwale and Siwale, 2017; Greenen, 2012).

Formalization risks entrenching existing power inequalities by confirming the more powerful and richer actors in their rights, as has been seen even where those elites are local, rather than multinational (Bulkan and Palmer, 2016). This risk can be increased by not recognising the existing informal institutions (Greenen, 2012).

Formalisation should be seen as a political, rather than technical process (Ofari et al., 2021).

External actors:

Partnering between large scale and artisanal mining development: One response taken, and at one time widely promoted, to formalising ASM has been for large scale mining companies to forge partnerships with these groups (Hilson et al., 2020; Sauerwein, 2023). Others have argued that such partnerships were not sustainable given the differences in scale and operating environments; artisanal miners were exploited for their local knowledge while prospecting and at risk of eviction following increases in gold prices or changes of ownership in mining companies (Hilson et al., 2020). Sauerwein (2023) documents an example where hosting of ASM by a large mining company, which initially led to positive community development and development of trust, rapidly collapsed after the change in ownership and drop in investment which contributed to a fatal accident.

A contrasting viewpoint is taken by Ofosu et al. (2025) who present partnership arrangements which address these issues by (i) legally recognising the partnership, (ii) identifying areas most suitable to ASM development and delineating these as protected areas; and (iii) ceding suitable and economically viable concessions to ASM and agreeing a revenue sharing arrangement. In this arrangement large scale mining companies would get financial return, while also saving labour and operational costs in these areas, and benefit from reduced conflict, while ASM would benefit from improved and more secure livelihoods.

Associations: Co-regulation through cooperatives (or other local groups, such as associations) has the potential to improve visibility and make ASM regulation easier (Mutemeri et al., 2016; Rodríguez et al., 2021; Cardoso and Hilson, 2022; Velez et al., 2025). Co-operatives can facilitate adaptive approaches and capacity building of local stakeholders, particularly in very poor communities with high levels of informal ASM (Mutemeri et al., 2016; Cardoso and Hilson, 2022). These groups also enable collective incentives which can improve efficiency by rewarding local agreements and cooperation among miners (Rodríguez et al.,2021). However, the cost and time to comply with standards and guidelines has also been a challenge for these groups and their success is highly dependent on the capacity of the group to build both

internal agreement and external influence (Rodríguez et al.,2021; Cardoso and Hilson, 2022; Velez et al., 2025).

Arthur-Holmes and Matey (2025) and Buss et al., (2019) identify how women are excluded from high earning activities in ASM and leadership structures and recommend that formalization interventions should address these gendered inequalities. Associations may provide one route to address this challenge as part of formalisation (Arthur-Holmes and Ofosu, 2024; Buss et al., 2019).

The equitable distribution of costs and benefits of compliance is highly dependent on leadership, in the worst case they can become organisations which facilitates the exploitation of the more vulnerable miners (De Haan et al., 2016; Rodríguez et al.,2021). Similarly, Corbett et al. (2017) outline how co-regulation of ‘designated areas’ for ASM has the potential to facilitate government regulation but can easily be monopolized by wealthier individuals and lead to greater inequality.

Reflections from expert review (Three No. Reviewers)

The focus on punishing artisanal miners can be seen as a continuation of the colonial legacy. Governments have encouraged large scale mining and foreign direct investment. Artisanal mining is seen as being in conflict with multi-national companies. Programmes for formalization are frequently rooted in neoliberalism and how to control market access. The high level of informality in the wider economy emphasizes how ‘formalization’ is used selectively to target specific groups. There are examples of ASM miners being used as scapegoats for environmental damage due to mining, as a target to redirect public anger. Punitive approaches to ASM haven't worked in terms of compliance but has worked in terms of political support. These punitive measures create an additional economy and attract resources. For example, licensing was considered the first step despite being a major barrier for the informal sector. Licensing required payment of fees and therefore provided an income generation opportunity.

The capacity for formalization is linked to macro issues, including the capacity of bureaucratic systems. The basic processes of the state need to be strengthened first to avoid any formalization process just becoming a block to changing practice. Softer approaches and capacity building were not visible and not valued. Some

actors have attempted to work with existing informal structures. However, support is limited. Funders, such as the World Bank, often do not have the flexibility or willingness to work with this level of ‘messiness’.

Mining policy needs to be developed to have differentiated formalisation requirements for different mining groups, from artisanal to large scale mining. This is often misunderstood and leads to policy errors, for example artisanal mining groups being required to pay for environmental permits in line with what would be expected for large-scale mining companies. Some locations have adapted policies which may make it easier to be formal but may not mean that miners are operating in a way that would prevent pollution. Formalisation may not imply eliminating negative externalities. For example, a miner can be “formal” in Guyana but still use mercury. A miner in Peru can be “in the process of formalizing” and have to comply with few actual requirements but still enjoy the same advantages of formal miners.

There are vested interests in maintaining the informal sector. The formal and informal sectors are intertwined, with the formal enabled by informal. The lack of accountability means that the risk still remains with the most vulnerable, who are working outside the law. For example, support to ASM miners is rapidly withdrawn in the face of public or funder opposition.

The main challenges are (i) how to get acceptance of informality, where the risk is held by the government, and (ii) how to also get the public to support policies which embrace the informal sector.

References

Andriamasinoro, F., Hohmann, A., Douguet, J. M., & Angel, J. M. (2020). Serious games as a social learning tool in formalizing the artisanal and small-scale mining sector in African territories. *The Extractive Industries and Society*, 7(3), 1108-1120.

Arthur-Holmes, F., & Matey, P. D. (2025). Gender as an entangled force: Gendered mining entanglements, labor organization and leadership structure in artisanal and small-scale mining. Journal of Rural Studies, 115, 103594.

Arthur-Holmes, F., & Ofosu, G. (2024). Rethinking state-led formalisation of artisanal and small-scale mining (ASM): Towards mining licence categorisation, women empowerment and environmental sustainability. *Resources Policy*, 93, 105058.

Atienza, M., Scholvin, S., Irarrazaval, F., & Arias-Loyola, M. (2023). Formalization beyond legalization: ENAMI and the promotion of small-scale mining in Chile. *Journal of Rural Studies*, 98, 123-133.

Ayambire, R. A., Nunbogu, A. M., Cobbinah, P. B., Kansanga, M. M., Pittman, J., & Dogoli, M. A. (2024). Constructing alternative interpretation: Embeddedness of illegality in small-scale mining. *The Extractive Industries and Society*, 17, 101430.

Bansah, K. J. (2023). Artisanal and small-scale mining formalization in Ghana: the government's approach and its implications for cleaner and safer production. *Journal of Cleaner Production*, 399, 136648.

Bulkan, J., & Palmer, J. (2016). Rentier nation: Landlordism, patronage and power in Guyana’s gold mining sector. *The Extractive Industries and Society*, *3*(3), 676-689.

Buss, D., Rutherford, B., Stewart, J., Côté, G. E., Sebina-Zziwa, A., Kibombo, R., ... & Lebert, J. (2019). Gender and artisanal and small-scale mining: implications for formalization. The Extractive Industries and Society, 6(4), 1101-1112.

Cardoso, M. E. G., & Hilson, G. (2022). Cooperatives as a centrepiece for formalizing small-scale mining in sub-Saharan Africa: Rationale, benefits and limitations. In *Routledge Handbook of the Extractive Industries and Sustainable Development* (pp. 419-439). Routledge.

Corbett, T., O'Faircheallaigh, C., & Regan, A. (2017). ‘Designated areas’ and the regulation of artisanal and small-scale mining. *Land Use Policy*, 68, 393-401.

De Haan, J., & Geenen, S. (2016). Mining cooperatives in Eastern DRC The interplay between historical power relations and formal institutions. *The Extractive Industries and Society*, 3(3), 823-831.

Geenen, S. (2012). A dangerous bet: The challenges of formalizing artisanal mining in the Democratic Republic of Congo. *Resources Policy*, 37(3), 322-330.

Heemskerk, M., Duijves, C., & Pinas, M. (2015). Interpersonal and institutional distrust as disabling factors in natural resources management: small-scale gold miners and the government in Suriname. *Society & Natural Resources*, 28(2), 133-148.

Hilson, G. (2020). The ‘Zambia Model’: A blueprint for formalizing artisanal and small-scale mining in sub-Saharan Africa?. *Resources Policy*, *68*, 101765.

Hilson, G. (2019). Why is there a large-scale mining ‘bias’ in sub-Saharan Africa?. Land use policy, 81, 852-861.

Hilson, G., & Laing, T. (2019). Guyana gold: a unique resource curse?. In *Why Does Development Fail in Resource Rich Economies* (pp. 55-74). Routledge.

Hilson, G., & Maconachie, R. (2017). Formalising artisanal and small-scale mining: insights, contestations and clarifications. *Area*, 49(4), 443-451.

Hilson, G., Sauerwein, T., & Owen, J. (2020). Large and artisanal scale mine development: The case for autonomous co-existence. *World Development*, *130*, 104919.

Hook, A. (2019). The multidimensionality of exclusion in the small-scale gold mining sector in Guyana: Institutional reform, landlordism, and mineral uncertainty. *World Development*, *123*, 104607.

Huntington, H., & Marple-Cantrell, K. (2021). Customary governance of artisanal and small-scale mining in Guinea: Social and environmental practices and outcomes. *Land use policy*, 102, 105229.

Jiménez, A. D., Smith, N. M., & Holley, E. A. (2024). Towards sustainable ASM-based livelihoods: The role of institutional arrangements in the formalization of artisanal and small-scale mining. *Resources Policy*, 94, 105129.

Keane, S., Bernaudat, L., Davis, K. J., Stylo, M., Mutemeri, N., Singo, P., ... & Etui, I. D. (2023). Mercury and artisanal and small-scale gold mining: Review of global use estimates and considerations for promoting mercury-free alternatives. *Ambio*, *52*(5), 833-852

Marshall, S., Taylor, K., Connor, T., Haines, F., & Tödt, S. (2022). Will Business and Human Rights regulation help Rajasthan's bonded labourers who mine sandstone?. *Journal of Industrial Relations*, 64(2), 248-271.

Martinez, G., Smith, N. M., & Malone, A. (2023). “I am formal, what comes next?”: A proposed framework for achieving sustainable artisanal and small-scale mining formalization in Peru. *The Extractive Industries and Society*, 13, 101227.

Mutemeri, N., Walker, J. Z., Coulson, N., & Watson, I. (2016). Capacity building for self-regulation of the Artisanal and Small-Scale Mining (ASM) sector: A policy paradigm shift aligned with development outcomes and a pro-poor approach. *The Extractive Industries and Society*, 3(3), 653-658.

Ofori, A. D., Mdee, A., & Van Alstine, J. (2021). Politics on display: The realities of artisanal mining formalisation in Ghana. *The Extractive Industries and Society*, *8*(4), 101014.

Ofosu, G., Arthur-Holmes, F., Siaw, D., & Sarpong, D. (2025). Friends or foes: Can large-scale mining companies partner with small-scale miners? Yes, they can?. Journal of Rural Studies, 119, 103709.

Rodriguez, L. A., Velez, M. A., & Pfaff, A. (2021). Leaders’ distributional & efficiency effects in collective responses to policy: Lab-in-field experiments with small-scale gold miners in Colombia. *World Development*, 147, 105648.

Sauerwein, T. (2023). Should mining companies partner with artisanal miners? Why only time will tell. *Journal of Rural Studies*, *100*, 103006.

Siegel, S., & Veiga, M. M. (2009). Artisanal and small-scale mining as an extralegal economy: De Soto and the redefinition of “formalization”. *Resources policy*, 34(1-2), 51-56.

Siwale, A., & Siwale, T. (2017). Has the promise of formalizing artisanal and small-scale mining (ASM) failed? The case of Zambia. *The Extractive Industries and Society*, 4(1), 191-201.

Spiegel, S., Keane, S., Metcalf, S., & Veiga, M. (2015). Implications of the Minamata Convention on Mercury for informal gold mining in Sub-Saharan Africa: from global policy debates to grassroots implementation?. *Environment, development and sustainability*, 17, 765-785.

Veiga, M. M., & Fadina, O. (2020). A review of the failed attempts to curb mercury use at artisanal gold mines and a proposed solution. *The Extractive Industries and Society*, *7*(3), 1135-1146.

Velez, M. A., Rueda, X., Henao, J. P., Monroy, D., Tobin, D., Maldonado, J., & Pfaff, A. (2025). Small-scale gold miners' preferences on formalization: First steps toward sustainable supply chains in Colombia. *World Development*, *188*, 106899

S.3-4: Informal transport.

Literature identified

Sources and steps are given in the PRISMA diagram below. The search string used was: [Informal* AND [Mobility OR Transport]] AND [Regulation OR Compliance] AND [impact OR evidence OR change OR increase].

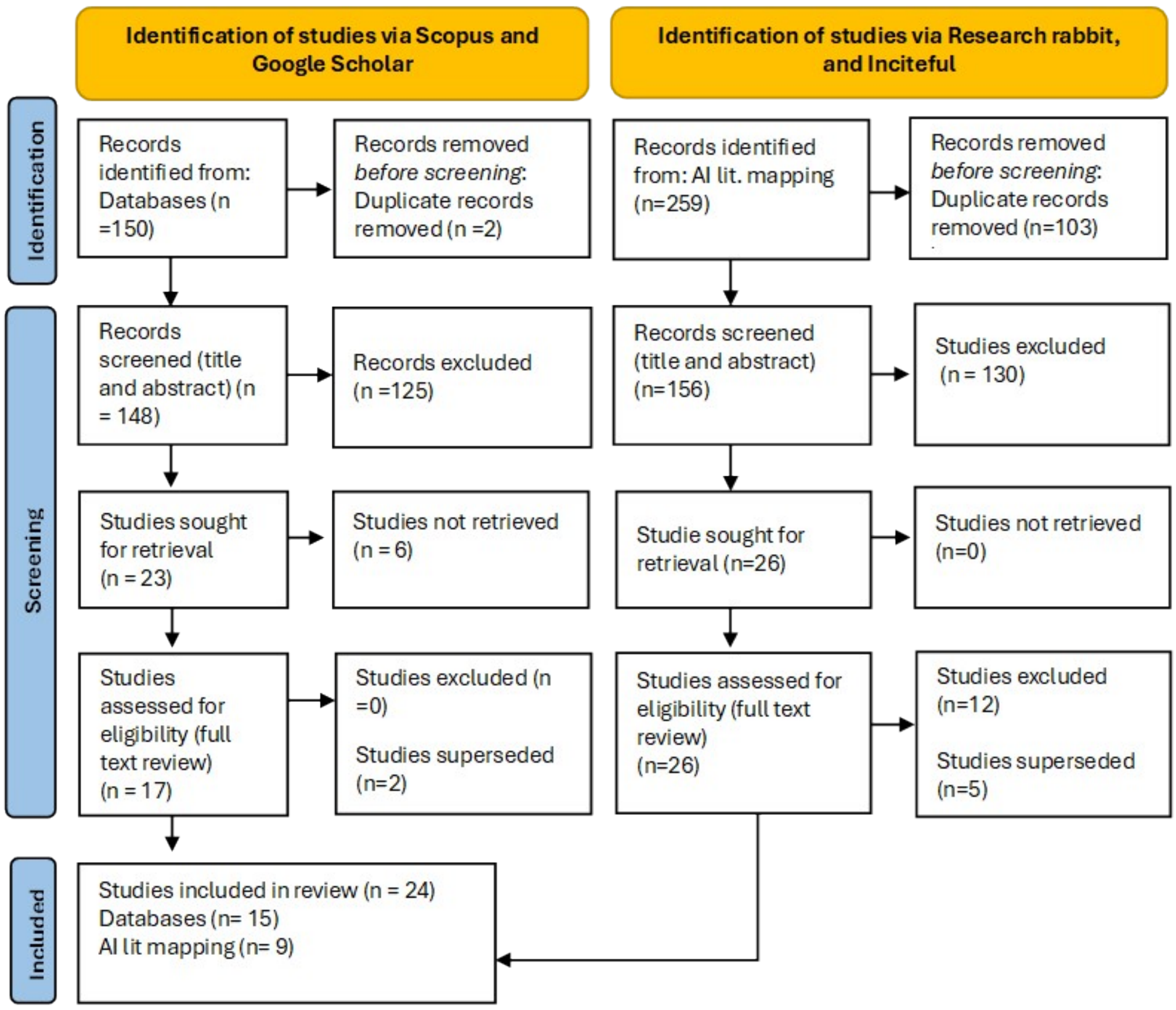

All informal or semi formal modes of transport are considered jointly in this summary. This simplification is noted as a limitation as semi-formal and informal bus operators (or paratransit) and motorbike taxis are addressed separately in the literature. This limitation is addressed by trying to identify only those issues which appear to be relevant across the sector. However, the flattening of variance between service provide providers through this review is also recognised as a limitation. Compliance

is considered largely in terms of safe services but also incorporates comments on workers' rights.

The rapid review findings incorporate 4 of the additional 5 recommended references from Expert feedback, with one duplicate removed. A separate section summarises further reflections from the expert feedback on research and progress in the sector.

Findings

Target group: Motivations. The majority of papers focused upon economic motivations for compliance (Ehebrecht et al., 2018; Akpa et al., 2019). Others looking at selective compliance across a range of regulations identified economic motivations as the main driver but also noted normative motives around perceived fairness and procedural justice (McCormick, 2014). Brehens et al. (2025) present how drivers paid through target setting and without any collective industry representation provide lower quality services than those paid with some element of regular salary and with collective representation. They conclude that 'better treated drivers provide better quality services', indicating a mix of motives.

Informal transport providers are presented by several authors as also being embedded social actors. Prices, services provided, access to jobs, ownership rights to vehicles, and vehicle routes, are often determined or mediated by personal connections (kinship and friendship), informal norms and informal institutions (Rekhviashvili and Sgibnev, 2020; Doherty, 2022; Rekhviashvili et al., 2022). Rekhviashvili et al. (2022) describe kin-based networks and resource-pooling to support drivers faced with road accidents or other uncertainties.

Doherty (2022) describes attempts to replicate some of this social embeddedness and trust through 'digital mediation' on ride hailing apps where drivers are ranked by passengers. They note that these reforms may be opposed by service providers based on their impact on the 'moral landscape' of the city, as well as the impacts on livelihoods and the economy (Doherty, 2022).

Target group: Knowledge of the rules and capacity to comply. Capacity to comply has been raised as a concern in the transition towards a more formal system, particularly by smaller operators (Mateo-Babiano et al., 2020; Guillen et al., 2024). Ardila-Gomez et al., (2025) highlight the high costs associated with formalisation, for

both the drivers and the vehicle owners. Informal transport providers are presented as being highly responsive to demand, adaptable to local contexts, efficient and flexible in operation, several papers raised the challenge of maintaining these benefits to the customer alongside formalization (Ehebrecht et al., 2018; Mittal et al., 2024).

Sumner et al. (2014) identify the limited impact of distributing protective equipment (hi-vis vests) on compliance and highlight that changing safety practice requires more than just removing economic barriers.

A number of authors note the importance of improving informal (or semiformal) transport services through business skills training, safety training for drivers, and basic infrastructure in operating environments, such as bus stops and stations (Schalekamp, 2017; Tun et al., 2020; Gatarin, 2024; Appiah-Opoku et al., 2025).

Perceptions of, and relationship with, the regulator. Attempts at formalisation have often led to resistance from transport providers (Shittu, 2014; Ardila-Gomez et al., 2025). Informal transport providers may have substantial social and political influence to disrupt services and mobilise for protests (Ardila-Gomez et al., 2025). Ferro et al. (2013) document the attempts and failure to exclude the informal transport sector. Top-down reform is similarly critiqued by Gatarin (2024). The success of efforts to regulate informal transport services will be highly dependent on the informal transport providers' participation and buy-in to these reforms (Shittu, 2014; Tun et al., 2020; Doherty, 2022; Gatarin, 2024).

Attempts to apply information-based regulation through ride hailing platforms, by either government or surrogate regulators, have been mixed, in some cases increasing inequality by excluding some transport providers (Doherty, 2022; Sopranzetti, 2022; Nguyen and Turner, 2024). Kuttler (2024) highlights how mobility apps are a regressive form of regulation, which increase pressure and effectively exploit transport workers, formal and informal. There are also concerns raised where these ride hailing apps are owned by international companies, bypassing government policy (Sopranzetti, 2022). However, there are significant advantages from the use of, and access to, routing information. Informal systems typically have very little available data, and routing information can improve safety, improve

efficiency and help inform future decisions by regulators (Doherty, 2022; Gaterin, 2024).

Regulation requires a credible threat of sanctions; as in other sectors, research indicates that heavy fines can increase the risk of corruption by increasing the level of bribes paid (Sohail et al., 2006). Chitere et al., (2013) identify that dealing with traffic police corruption, and employer target setting which incentivises unsafe practices in drivers, should take precedence over enforcement and penalties.

Resourcing and supporting regulators. Reforming public transport means that the regulators and other relevant institutions need to be strengthened (McCormick, 2014; Ardila-Gomez et al., 2025). The lack of financial and institutional capacity to implement reforms, such as formalisation and integration of transport services is noted as a limitation (McCormick, 2014; Mateo-Babiano et al., 2020; Tun et al., 2020). The lack of reliable data on service use, safety, costs and charges is mentioned across multiple papers as a barrier to effective regulation.

Enabling Environment. Informal modes of transport, particularly moto-taxis are often not considered in transport planning and there is often no experience with their effective regulatory control (Ehebrecht et al., 2018; Guillen et al., 2024). Rekhviashvili et al. (2022) go further in describing the use of 'informality' as an 'instrument of exclusion', an active marginalization, rather than benign neglect, of this group.

Informal transport providers are often highly integrated with formal public transport services; working with and around existing legal frameworks (Shittu, 2014; Ehebrecht et al., 2018; Rekhviashvili et al.,2022). Perspectives on whether the enabling environment should support this complementarity or incentivize competition appear to be mixed. A push for legalization and policies to foster competition for both the formal and informal sector has been promoted as a route to avoid cartels and keep costs low (Golub et al., 2009). However more recent literature appears to move towards accepting a certain level of informality and support complementarity, while building incremental improvements in practice (Schalekamp, 2017; Tun et al., 2020; Rekhviashvili et al., 2022; Gatarin, 2024; Guillen et al., 2024)

Gatarin (2024) frames urban mobility as human dignity and highlights the need for the sector to consider public subsidies to improve conditions for informal transport providers and services. Public investment would be needed to provide equity for transport providers, by supporting vehicle maintenance and safety measures, and transport users, by maintaining fares at affordable prices and ensuring services extend into all areas (Tun et al., 2020; Kerzhner, 2023; Gatarin, 2024; Ardila-Gomez et al., 2025).

External actors. Safety is noted as a major concern which could influence public use of the informal sector and increase support for regulation (Akpa et al., 2019; Alimo et al., 2022). However, even where users support regulation in theory, usage patterns were unchanged unless the safer alternative was also affordable and convenient (Alimo et al., 2022).

The underlying drivers for unsafe informal transport are social, low-income areas have difficult access and are not served by formal routes, pushing low-income groups onto unsafe feeder services (Alimo et al., 2022; Ardila-Gomez et al., 2025). Regulation of the sector should not be seen in isolation, but as part of wider urban development efforts (Ardila-Gomez et al., 2025).

Associations, unions or cooperatives. Associations, unions or cooperatives can have an important role in professionalising informal or semi formal transport providers, both improving driver safety and to enable informal providers to complement formal transport services (Heinrichs et al., 2017; Ntramah et al., 2023).

There are examples of strong co-regulation around the use of protective equipment and compliance with traffic rules (Ntramah et al., 2023).

Cooperatives in Kenya have increased compliance by operator consolidation, shifting all drivers onto salaries, vehicle monitoring and enforcing centralised saving funds to cover maintenance and depreciation costs (Behrens et al., 2017).

The example of cooperatives in Kenya is partly successful due to the institutional support provided for cooperatives (Behrens et al., 2017). Similarly, both Kerzhner (2023) and Heinrichs et al. (2017) identify the need for support from local government, professionalization and access to resources for groups of service providers to be successful.

Abridged Expert feedback (Six No. - Three with substantive narrative review)

Informal transport service providers should be seen as both social and economic actors. Not one or the other. Informal transport may start as a community service and because of the lack of proper roads. However, they are not working at a loss.

Without Government intervention to reduce costs to users, low-income groups can be expected to continue using less safe cheaper alternatives. Safety is a trade-off.

Cooperatives have led to improvements in driver safety through internal rules and sanctions. They also protected drivers from regulator abuse. Cooperatives have enabled informal service providers to diversify into other linked businesses – such as running petrol stations. However, there are also examples of where funds have been mismanaged or elected officers have committed fraud. In some cities informal providers have reached a critical mass and can influence the Government. This group, particularly through the cooperatives formed, has been problematic in blocking some transport upgrading. They required, compensation if they were to be displaced by formal systems. However overall cooperatives have been seen as an effective way to group and regulate a highly dispersed sector.

Capacity to comply has been an issue. The cost of compliance, and the feasibility for both service provider and user, are rarely fully considered by the Government. Examples were given from the Philippines of brand-new vehicles, GPS, WiFi and card payment being required for the formalization process, which were difficult to implement and maintain.

As reported in a number of countries the relationship with the regulator is often punitive. The Government rarely holds registers of drivers and are not able to provide rewards for sustained compliance, however fining drivers for instances of non-compliance is easy.

Currently the informal sector is providing a needed service with no Government intervention. A clear recommendation from the Thredbo 2025 Conference International Conference Series on Competition and Ownership in Land Passenger Transport was that the informal transport sector should be included and supported, because they are providing a service which the Government cannot.

References

Akpa, N. E. E., Booysen, M. J., & Sinclair, M. (2019). Fuel savings as an incentive for speed compliance in the informal public transport industry in South Africa. In *2019 IEEE Intelligent Transportation Systems Conference*, October (Itsc) (pp. 492-496). IEEE.

Alimo, P. K., Rahim, A. B. A., Lartey-Young, G., Ehebrecht, D., Wang, L., & Ma, W. (2022). Investigating the increasing demand and formal regulation of motorcycle taxis in Ghana. *Journal of Transport Geography*, 103, 103398.

Appiah-Opoku, S., Manu, K. K., & Sampson, L. E. (2025). *Assessing motorcycle taxis as public transport option in the urban life of African cities: The case of Accra, Ghana*. March 2025

Ardila-Gomez, A., & Darido, G. (2025). Public Transport Reform in Developing Countries: Lessons from Experience. *Transportation Research Procedia*, 82, 2737-2749.

Behrens, R., McCormick, D., Orero, R., & Ommeh, M. (2017). Improving paratransit service: Lessons from inter-city matatu cooperatives in Kenya. *Transport Policy*, 53, 79-88.

Behrens, R., Zuidgeest, M., & Durant, T. (2025). Relationships between paratransit passenger satisfaction and driver labour conditions in Sub-Saharan Africa. *African Transport Studies*, *3*, 100030.

Chitere, P., Orero, R., Mitullah, W., McCormick, D., & Ommeh, M. (2013). Paratransit labour and regulatory compliance. Institute for Development Studies, University of Nairobi.

Doherty, J. (2022). Motorcycle taxis, personhood, and the moral landscape of mobility. *Geoforum*, 136, 242-250.

Ehebrecht, D., Heinrichs, D., & Lenz, B. (2018). Motorcycle-taxis in sub-Saharan Africa: Current knowledge, implications for the debate on “informal” transport and research needs. *Journal of transport geography*, 69, 242-256.

Ferro, P. S., Behrens, R., & Wilkinson, P. (2013). Hybrid urban transport systems in developing countries: Portents and prospects. *Research in Transportation Economics*, 39(1), 121-132.

Gatarin, G. R. (2024). Modernising the 'king of the road': Pathways for just transitions for the Filipino jeepney. *Urban Governance*, 4(1), 37-46.

Golub, A., Balassiano, R., Araújo, A., & Ferreira, E. (2009). Regulation of the informal transport sector in Rio de Janeiro, Brazil: welfare impacts and policy analysis. *Transportation*, 36, 601-616.

Guillen, M. D. V., Gaspay, S. M., Mateo-Babiano, I., & Schneck, J. A. T. (2024). Formalizing the Informal in Public Transportation: The Case of Motorcycle Taxis Transport Network Service in the Philippines. Conference paper. Thredbo 2025 Conference International Conference Series on Competition and Ownership in Land Passenger Transport. Cape Town.

Heinrichs, D., Goletz, M., & Lenz, B. (2017). Negotiating territory: Strategies of informal transport operators to access public space in urban Africa and Latin America. *Transportation Research Procedia*, 25, 4507-4517.

Kerzhner, T. (2023). How are informal transport networks formed? Bridging planning and political economy of labour. *Cities*, 137, 104348.

Kuttler, T. (2024). Urban mobilities in Mumbai: Towards worker-centric platformisation beyond 'urban solutionism'. *Urban Studies*, 00420980241264645.

Mateo-Babiano, I., Recio, R. B., Ashmore, D. P., Guillen, M. D., & Gaspay, S. M. (2020). Formalising the jeepney industry in the Philippines–A confirmatory thematic analysis of key transitionary issues. *Research in transportation economics*, 83, 100839.

McCormick, D. (2014). Institutional development in public transport: implications of selective compliance for Nairobi's paratransit system. Institute for Development Studies, University of Nairobi.

Mittal, K. M., Timme, M., & Schröder, M. (2024). Efficient self-organization of informal public transport networks. *Nature Communications*, 15(1), 4910.

Nguyen, B., & Turner, S. (2024). The New Kids on the Street: Ride-Hailing Platform Drivers Competing with Informal Motorbike-Taxi Livelihoods in Hanoi, Vietnam. In *Geographies of the Platform Economy: Critical Perspectives* (pp. 173-187). Cham: Springer International Publishing.

Ntramah, S., Peters, K., Jenkins, J., Mugisha, M. M., Chetto, R., Owino, F., ... & Johnson, T. (2023). Safety, health and environmental impacts of commercial motorcycles in Sub-Saharan African cities. *Urban, Planning and Transport Research*, 11(1), 2259233.

Rekhviashvili, L., & Sgibnev, W. (2020). Theorising informality and social embeddedness for the study of informal transport. Lessons from the marshrutka mobility phenomenon. *Journal of Transport Geography*, 88, 102386.

Rekhviashvili, L., Kębłowski, W., Sopranzetti, C., & Schwanen, T. (2022). Informalities in urban transport: Mobilities at the heart of contestations over (in) formalisation processes. *Geoforum*, 136, 225-231.

Schalekamp, H. (2017). Lessons from building paratransit operators' capacity to be partners in Cape Town's public transport reform process. *Transportation Research Part A: Policy and Practice*, 104, 58-66.

Shittu, A. O. (2014). Towards quasi-formal management of paratransit in informal transport dependent cities of Nigeria. *IOSR Journal of Humanities and Social Science* (IOSR-JHSS), 19, 75-80.

Sohail, M., Maunder, D. A. C., & Cavill, S. (2006). Effective regulation for sustainable public transport in developing countries. *Transport policy*, *13*(3), 177-190.

Sopranzetti, C. (2022). Shifting informalities: Motorcycle taxis, ride-hailing apps, and urban mobility in Bangkok. *Geoforum*, 136, 293-301.

Sumner, S. A., Pallangyo, A. J., Reddy, E. A., Maro, V., Pence, B. W., Lynch, C., ... & Thielman, N. M. (2014). Effect of free distribution of safety equipment on usage among motorcycle–taxi drivers in Tanzania—A cluster randomised controlled trial. *Injury*, 45(11), 1681-1686.

Tun, T. H., Welle, B., Hidalgo, D., Albuquerque, C., Castellanos, S., Sclar, R., & Escalante, D. (2020). *Informal and semiformal services in Latin America: an overview of public transportation reforms*. InterAmerican Development Bank.

S.3-5: Off grid water

Literature identified

Sources and steps are given in the PRISMA diagram below. The search string used was: [vendor OR informal OR illegal OR off-grid] AND [water AND [network OR supply]] AND [Regulation OR Compliance] AND [impact OR evidence OR change OR increase].

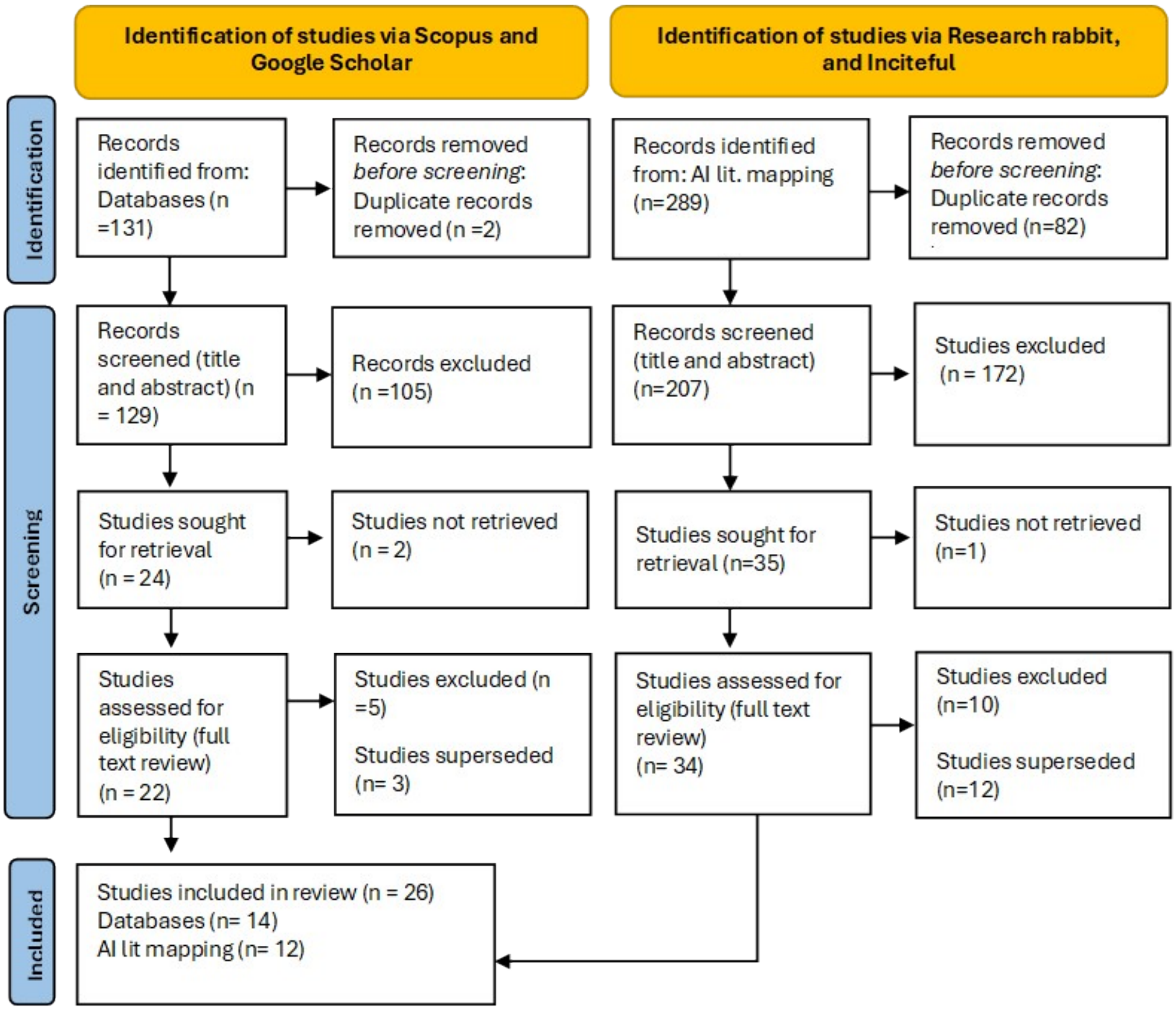

The below summary includes all 'alternative providers' outside of the networked system; tankered water, water vendors, small networks reselling bulk water and other forms of modular and decentralised water systems (Gerlach, 2008). The review focuses on rapidly growing urban or peri-urban areas. Only commercial providers are considered. Communal, community or NGO managed water supplies are not included in this review. The review focusses on situations where these suppliers are extending into low-income areas, rather than providing services to high- or middle-

income areas. Service providers are therefore considered where they are potentially complementary, rather than directly competing with a utility supply.

The review therefore includes formal and informal suppliers, working commercially, who are distributing utility supplies (formally or informally) and those distributing water from independent source development. There is a high level of variance within this group in terms of capacity and influence. The impact of this variance is discussed where possible within the confines of this rapid review. However, the flattening of variance between service provide providers through this review is also recognised as a limitation. It is considered an acceptable limitation as the World Bank review also combined recommendations for this group (Misra and Kingdom, 2022).

The regulation of bottled or sachet water supplied by large private sector companies is not considered here as being fundamentally different to off-grid supplies through small or informal providers (Pacheco-Vega, 2019).

Compliance is with water quality standards and pricing (Weissenberger and Elliott, 2024). Poor water quality and exploitative fees are the concerns most commonly raised with regards to alternative providers (Weissenberger and Elliott, 2024).

The literature maps the gradual acceptance of alternative providers as part of the water market and the emergence of different models for water provision (Kjellén and McGranahan, 2006; Weissenberger and Elliott, 2024). Studies highlight alternative providers as an emerging issue in high income as well as lower- or middle-income areas (Wutich et al., 2023). Only 26 papers were found which implicitly or explicitly discussed regulation or building compliance of alternative providers. An additional 7 publications looking at regulation and utility led services are incorporated, prompted by the Expert feedback. No studies were found which claimed to have identified a causal link between an intervention and increased compliance of service providers.

The rapid review findings incorporate the additional 10 recommended references from Expert feedback. A separate section summarises further reflections from the expert feedback on research and progress in the sector. The overall findings below are supported by practitioner literature, including World Bank guidance (Misra and Kingdom, 2022).

Findings

Target group: Motivations. There are conflicting findings around economic motives. Some authors highlight exploitative relationships between alternative providers and customers, with the primary drivers of alternative providers being profit (Gerlach, 2008; Sarkar, 2020). Others describe economic drivers being around stability and continued operation, rather than the very narrow profit margins (Gerlach, 2008).

Several authors describe how alternative providers determine customer charges and credit schemes based on personal relationships, or based on need, or on religion, reflecting normative beliefs about the right to water, in addition to the economic drivers to recoup costs and develop a livelihood (Gero et al., 2014; Alba et al., 2019; Alba et al., 2022). Similarly, Alba et al. (2019 & 2022) report a range of social and normative motivations for whether and how individuals work for an alternative provider, in addition to economic motives. They identify motives including stability, status, habits, trust and social relationships, affiliation and the feeling of contributing to a greater good or a broader sense of social responsibility.

Beresford et al (2024) also highlight the importance of normative motives. They identify multiple examples of the moral economy for water, where normative beliefs in a right to water 'facilitate practices that keep water prices affordable and water distribution seen as fair'. Similarly, Wutich et al. (2016) describe how water vendors see themselves as addressing moral injustice by providing safe and affordable water services to unserved communities. Moral economies and normative regulation are less likely to develop in heterogenous and less integrated communities, however there are indications that they can be supported via social mobilisation (Beresford et al., 2024).

Target group: Knowledge of the rules and capacity to comply. Although alternative providers may have limited technical capacity, they have, possibly substantial, skills in working with their target group, including market knowledge, customer responsiveness, ability to adapt to provide services in low-income areas and trust of communities (Gerlach, 2008; Gero et al., 2014). Recommended regulatory approaches recognize and work with the skills and ability of alternative providers (Gerlach, 2008; Gero et al., 2014).

The importance of clear guideline and standards, and active dissemination, are identified alongside the need to understand and build levels of awareness and capacity to comply (Gerlach, 2008; Dakyaga et al., 2018; Mapunda et al., 2018; Alba et al., 2019).

Enabling alternative providers can be an effective way of broadening the utility's reach (Gilson and Garrick, 2024). However, this does entail shifting the risks associated with providing the service to the alternative provider, which may not be a just or moral step (Adams et al., 2019; Boakye-Ansah et al, 2019b). Active regulation, alongside enabling and support to alternative providers, is needed for price or quality control of the extended services (Boakye-Ansah et al, 2019b).

Perceptions of, and relationship with, the regulator. Alternative providers are now recognised to be an important complement to formal piped water networks, both in terms of geographical reach and to fill gaps created by intermittent supply (Adams et al., 2019; Weissenberger and Elliott, 2024). The attitude of utilities towards alternative providers has shifted, from seeing them as a problem to be displaced, towards seeing them as potential partners or sub-contractors who can help utilities in meeting their conflicting aims of commercial viability and public good (Bhatt, 2014; Boakye-Ansah et al, 2019b; Alba et al., 2022; Karpouzoglou et al., 2023; Weissenberger and Elliott, 2024).

This shift has happened alongside the growing acceptance of alternative providers as part of water provision, at least in the short or medium term. The level of importance of alternative providers is disputed, with some seeing them as an essential part of service provision in low-income areas and others as a short-term solution which will – and should - be displaced as utilities extend and improve their services (Boakye-Ansah et al., 2019a; Mitlin and Walnycki, 2020; Sarkar, 2020; Gerlach, 2008). There are also examples of regulators and utilities increasingly recognising their responsibility to provide universal access and taking positive steps towards this goal, including clear mandates for universal access, pro-poor indicators and low-income consumer units, and in some cases substantial public funding and government leadership (WSUP, 2018; Boakye-Ansah et al., 2022; ESAWAS, 2022; Vathanan and Dasgupta, 2025).

Top-down attempts to regulate the disparate and highly mobile alternative providers has often not been successful, due to the difficulty of enforcement (Zozmann et al., 2022). Participatory approaches, and building bottom-up compliance, are essential (Wutich et al., 2023).

Alternative providers should not be sanctioned where they are filling a gap left by the water utility and form an important part of household water security (Gerlach, 2008; Gilson and Garrick, 2024). Active recognition of alternative providers may be required in order to enable regulation and public information. Passive - or unofficial - recognition of alternative providers can expose them to harassment from authorities and prevent consumers being fully informed about the risks (Gilson and Garrick, 2024).

Active recognition of alternative providers is considered beneficial for those providers, if given alongside business and capacity support (Matsinhe et al., 2008; Gerlach, 2008; Dakyaga et al., 2018; Mapunda et al., 2018; Gilson and Garrick, 2024). However, official recognition may not have any impact for the consumer unless there is also regulation in price and quality control (Gilson and Garrick, 2024). There is a continued lack of policies or regulations specific to alternative providers (Mapunda et al., 2018; Priadi et al., 2024).

Engaging in both formal and informal service delivery is required to enhance service delivery (Dery and Bisung, 2022). Several authors note that networked and off-grid water services are intertwined and interdependent in a legal grey zone, and the use of binary formal / informal distinctions is not helpful (Schwartz et al., 2015; Truelove, 2019; Alba et al., 2022; Zozmann et al., 2022; Karpouzoglou et al., 2023). This intermeshed system of formal and alternative providers can be highly political and may cater to the vested interests of more powerful groups (Schwartz et al., 2015; Truelove, 2019; Adams et al., 2019; Karpouzoglou et al., 2023).

Enabling Environment and External actors. The wider enabling environment for building compliance of service providers can be understood in terms of the commodification of water, where economic equity may be considered to have replaced social equity and the focus is on consumers rather than citizens (Menga et al., 2023). This global framing is in conflict with access for water as a normative motive for service providers, described above. It is also in conflict with the greater

focus on extending services into low-income areas also described above. However, it may underpin the lack of resourcing for national regulators and utilities to provide universal access (Menga et la., 2023).

Accountability of alternative providers is typically towards the customer, rather than the government. The decentralisation of the regulatory role, and the potential for local customer groups to take a role as a surrogate regulator is identified by several authors (Gerlach and Franceys, 2010; Pakizer and Lieberherr, 2018).

Two potential routes to increase accountability are outlined. Firstly, disclosure of service performance and cost information and strengthened customer protection, leading to customers holding service providers to account (Matsinhe et al., 2008; Gerlach and Franceys, 2010 Gerlach, 2008). The disclosure of cost information should include both cost and charge information, making mark-ups by alternative providers clear (Gerlach, 2008). This information-based regulation can be an interim step where direct economic regulation is not possible (Gerlach, 2008). Secondly the engagement of CSOs, CBOs or NGOs as surrogate regulators to provide technical and financial support, monitor and provide oversight – holding both alternative providers and local government to account (Matsinhe et al., 2008; Gero et al., 2014; Pakizer and Lieberherr, 2018). Multilayered systems where community water user associations are supported by NGOs, local water boards or local government appear to be more successful (Adams et al., 2019).

Resourcing and supporting regulators. Water regulators are typically not resourced to regulate the diverse and dispersed market of alternative providers, they do not have the needed information or networks (Gerlach, 2008; Mapunda et al., 2018). Those who have been able to engage alternative providers successfully, typically received external support which enabled this transition, for example NWASCO in Zambia (Gerlach and Franceys, 2010).

Delegated management of alternative providers, for example to the community or local civil society groups, often means delegating to groups with lower capacity than the utility (Schwartz et al., 2017; Boakye-Ansah, 2019a; Adams et al., 2019; Gilson and Garrick, 2024). Delegated management often struggles to be financially and technically viable (Adams et al., 2019). Groups charged with local regulation need to

be adequately resourced to avoid being exposed to regulatory capture (Matsinhe et al., 2008; Gerlach, 2008).

Co-regulation through associations, unions or cooperatives. Co-regulation of water tankers through associations is a common recommendation as part of engaging and regulating alternative providers (Dakyaga et al., 2018; Mapunda et al., 2018; Zozmann et al., 2022). Association members are noted as developing agreed minimum standards and prioritising water quality (Alba et al., 2019). Similarly, comparisons of unionised and non-unionised water vendors indicate that unionised water vendors typically create and enforce rules and norms that facilitate higher levels of accountability, and a lower cost, more reliable service (Wutich et al., 2016; Beresford et al., 2024). Both tanker associations and water vendor unions are credited with facilitating social networks for business development and creating shared social norms around how to provide services and engage with customers (Wutich et al., 2016; Alba at al., 2019).

The structure, and ways of working, of alternative providers need to be understood by local authorities in order to regulate the sector (Choueiri and Vantaggiato, 2023). Although some case studies describe no risk of collusion, co-regulation can introduce risks due to monopoly formation, resulting in higher prices and potentially even denial of services (Wutich et al., 2016; Sarkar, 2020). Co- and self-regulation also still require monitoring and oversight in order to be effective (Gerlach, 2008). As part of support to co- and self-regulation it would be beneficial to raise awareness among the customer base about the relative service quality from alternative providers (Wutich et al., 2016).

Reflections from Expert feedback (Four No. Responses)

Expert feedback on this rapid review emphasised the importance of continuing to focus on universal access to safe water supply and presented a centralised high-capacity formal service provider as the main, if not only, way to achieve this long-term goal. Experts highlighted a "*tendency for researchers to be overly optimistic in their view of alternative providers*". "*A pluralistic and decentralized system has*

advantages but can also result in stagnation at a low level of service". In addition, the view of informal providers should not be naive; some may be actively criminal.

Expert feedback emphasized that "*the art of regulating is to enable the utility to undertake its responsibilities"*. Part of this process has been accepting alternative service providers in the transition to full utility service. However, the important point is that the utility understands that they are responsible for overseeing the alternative providers in their area, alongside extending their own services. Alternative providers facilitate the transition and make possible what otherwise becomes an impossible regulatory task. A high-capacity formal utility providing universal access to good quality services should always be the preferred option in the long term.

Expert feedback also highlighted the challenges in drawing common conclusions across such a disparate and heterogenous group of service providers.

References

Adams, E. A., Sambu, D., & Smiley, S. L. (2019). Urban water supply in Sub-Saharan Africa: historical and emerging policies and institutional arrangements. *International Journal of Water Resources Development*, 35(2), 240-263.

Alba, R., Bruns, A., Bartels, L. E., & Kooy, M. (2019). Water brokers: Exploring urban water governance through the practices of tanker water supply in Accra. *Water,* 11(9), 1919.

Alba, R., Kooy, M., & Bruns, A. (2022). Conflicts, cooperation and experimentation: Analysing the politics of urban water through Accra's heterogeneous water supply infrastructure. *Environment and Planning E: Nature and space*, 5(1), 250-271.

Beresford, M., Brewis, A., Choudhary, N., Drew, G., Garcia, N. E., Garrick, D., ... & Wutich, A. (2024). Justice and moral economies in "Modular, Adaptive, and Decentralized"(MAD) water systems. *Water Security*, 21, 100148.

Bhatt, J. (2014). Comparison of small-scale providers' and utility performance in urban water supply: the case of Maputo, Mozambique. *Water Policy,* 16(1), 102-123.

Boakye-Ansah, A. S., Schwartz, K., & Zwarteveen, M. (2019a). Unravelling pro-poor water services: what does it mean and why is it so popular?. *Journal of Water, Sanitation and Hygiene for Development,* 9(2), 187-197.

Boakye-Ansah, A. S., Schwartz, K., & Zwarteveen, M. (2019b). From rowdy cartels to organized ones? The transfer of power in urban water supply in Kenya. *The European Journal of Development Research,* 31(5), 1246-1262.

Boakye-Ansah, A. S., Schwartz, K., & Zwarteveen, M. (2022). Service differentiation as an improvement strategy for access to water in urban low-income areas: evidence from three Kenyan cities. *International Journal of Water Resources Development*, 38(5), 766-782.

Choueiri, Y., & Vantaggiato, F. (2023). Informal Water Tankers, Their Network Structure, and Drivers of Cooperation and Competition: A Case Study in Beirut, Lebanon. *Society & Natural Resources*, 36(12), 1530-1550.

Dakyaga, F., Kyessi, A. G., & Msami, J. M. (2018). Water access today and tomorrow: Domestic water sustainability under informal water supply markets in Dar es Salaam, Tanzania. *Journal of Sustainable Development*, 11(6), 120.

Dery, F., & Bisung, E. (2022). Exploring informalities, inequalities, and health risk in the informal water sector in Sub-Saharan Africa. *Water Security*, 17, 100128.

ESAWAS (2022) The Water Supply and Sanitation Regulatory Landscape Across Africa 2022. Eastern and southern Water and Sanitation Regulators Association. Available at: https://www.esawas.org/wp-content/uploads/2024/04/Esawas_Report_2022.pdf (accessed 19 June 2025)

Gerlach, E. (2008). Regulating Alternative Providers for the Poor. In Franceys, R. & Gerlach, E. (Eds.) *Regulating Water and Sanitation for the Poor* (pp. 195-209). Routledge.

Gerlach, E., & Franceys, R. (2010). Regulating water services for all in developing economies. *World development*, 38(9), 1229-1240.

Gero, A., Carrard, N., Murta, J., & Willetts, J. (2014). Private and social enterprise roles in water, sanitation and hygiene for the poor: a systematic review. *Journal of Water, Sanitation and Hygiene for Development,* 4(3), 331-345.

Gilson, G. G., & Garrick, D. (2024). Co-producing water service delivery: a scoping review and typology of informal water vending in Sub-Saharan Africa. *Environmental Research Letters*, 20(1), 013004.

Karpouzoglou, T., Vij, S., Blomkvist, P., Juma, B., Narain, V., Nilsson, D., & Sitoki, L. (2023). Analysing water provision in the critical interface of formal and informal urban water regimes. *Water international,* 48(2), 202-216.

Kjellén, M. & McGranahan, G. (2006) *Informal water vendors and the urban poor,* International Institute for Environment and Development London, 2006. Available at: https://www.iied.org/10529iied (accessed May 3, 2024).

Mapunda, D. W., Chen, S. S., & Yu, C. (2018). The role of informal small-scale water supply system in resolving drinking water shortages in peri-urban Dar Es Salaam, Tanzania. *Applied Geography*, 92, 112-122.

Matsinhe, N. P., Juízo, D., Macheve, B., & dos Santos, C. (2008). Regulation of formal and informal water service providers in peri-urban areas of Maputo, Mozambique. *Physics and Chemistry of the Earth,* Parts a/b/c, 33(8-13), 841-849.

Menga, F., Rusca, M., & Alba, R. (2023). Philantrocapitalism and the re-making of global water charity. *Geoforum*, 144, 103788.

Misra, S. & Kingdom, B. (2022). *Citywide inclusive water supply: The role of supplementary urban water service providers*. World Bank. Washington DC.

Mitlin, D., & Walnycki, A. (2020). Informality as experimentation: water utilities' strategies for cost recovery and their consequences for universal access. *The Journal of Development Studies*, *56*(2), 259-277.

Pacheco-Vega, R. (2019). (Re) theorizing the politics of bottled water: water insecurity in the context of weak regulatory regimes. *Water*, 11(4), 658

Pakizer, K., & Lieberherr, E. (2018). Alternative governance arrangements for modular water infrastructure: An exploratory review. *Competition and Regulation in Network Industries,* 19(1-2), 53-68.

Priadi, C. R., Suleeman, E., Darmajanti, L., Putri, G. L., Genter, F., Foster, T., & Willetts, J. (2024). Policy and regulatory context for self-supplied drinking water services in two cities in Indonesia: Priorities for managing risks. *Environmental Development*, 49, 100940.

Sarkar, A. (2020). Informal water vendors and the urban poor: evidence from a Nairobi slum. *Water International*, 45(5), 443-457. 1.

Schwartz, K., Tutusaus Luque, M., Rusca, M., & Ahlers, R. (2015). (In) formality: the meshwork of water service provisioning. Wiley Interdisciplinary Reviews: *Water*, 2(1), 31-36.

Schwartz, K., Tutusaus, M., & Savelli, E. (2017). Water for the urban poor: Balancing financial and social objectives through service differentiation in the Kenyan water sector. *Utilities Policy*, 48, 22-31.

Truelove, Y. (2019). Gray zones: The everyday practices and governance of water beyond the network. *Annals of the American Association of Geographers*, 109(6), 1758-1774.

Vathanan, G. M., & Dasgupta, S. (2025). *Decentralisation and Empowerment: The Odisha Model for Urban Development*. IWA Publishing, London.

Weissenberger, L. T., & Elliott, S. J. (2024). Gender dimensions of water vending in LMICs: A scoping review. *Water Security*, 23, 100186.

WSUP (2018). Institutionalising pro-poor services: A new Key Performance Indicator for Kenyan utilities. Water and Sanitation for Urban Populations. Available at: https://wsup.com/publications/institutionalising-pro-poor-services-a-new-key-performance-indicator-for-kenyan-utilities/ (accessed 19 June 2025)

Wutich, A., Beresford, M., & Carvajal, C. (2016). Can informal water vendors deliver on the promise of a human right to water? Results from Cochabamba, Bolivia. *World Development*, 79, 14-24.

Wutich, A., Thomson, P., Jepson, W., Stoler, J., Cooperman, A. D., Doss-Gollin, J., ... & Westerhoff, P. (2023). MAD water: Integrating modular, adaptive, and decentralized approaches for water security in the climate change era. *Wiley Interdisciplinary Reviews: Water*, 10(6), e1680.

Zozmann, H., Morgan, A., Klassert, C., Klauer, B., & Gawel, E. (2022). Can tanker water services contribute to sustainable access to water? A systematic review of case studies in urban areas. *Sustainability,* 14(17), 11029.

S3-6: Municipal solid waste management (waste pickers)

Literature identified

Sources and steps are given in the PRISMA diagram below. The search string used was: [[municipal AND solid AND waste AND management] OR [waste AND pickers]] AND [Regulation OR Compliance] AND [impact OR evidence OR change OR increase]. Compliance is considered around safe working practices in solid waste collection and recycling.

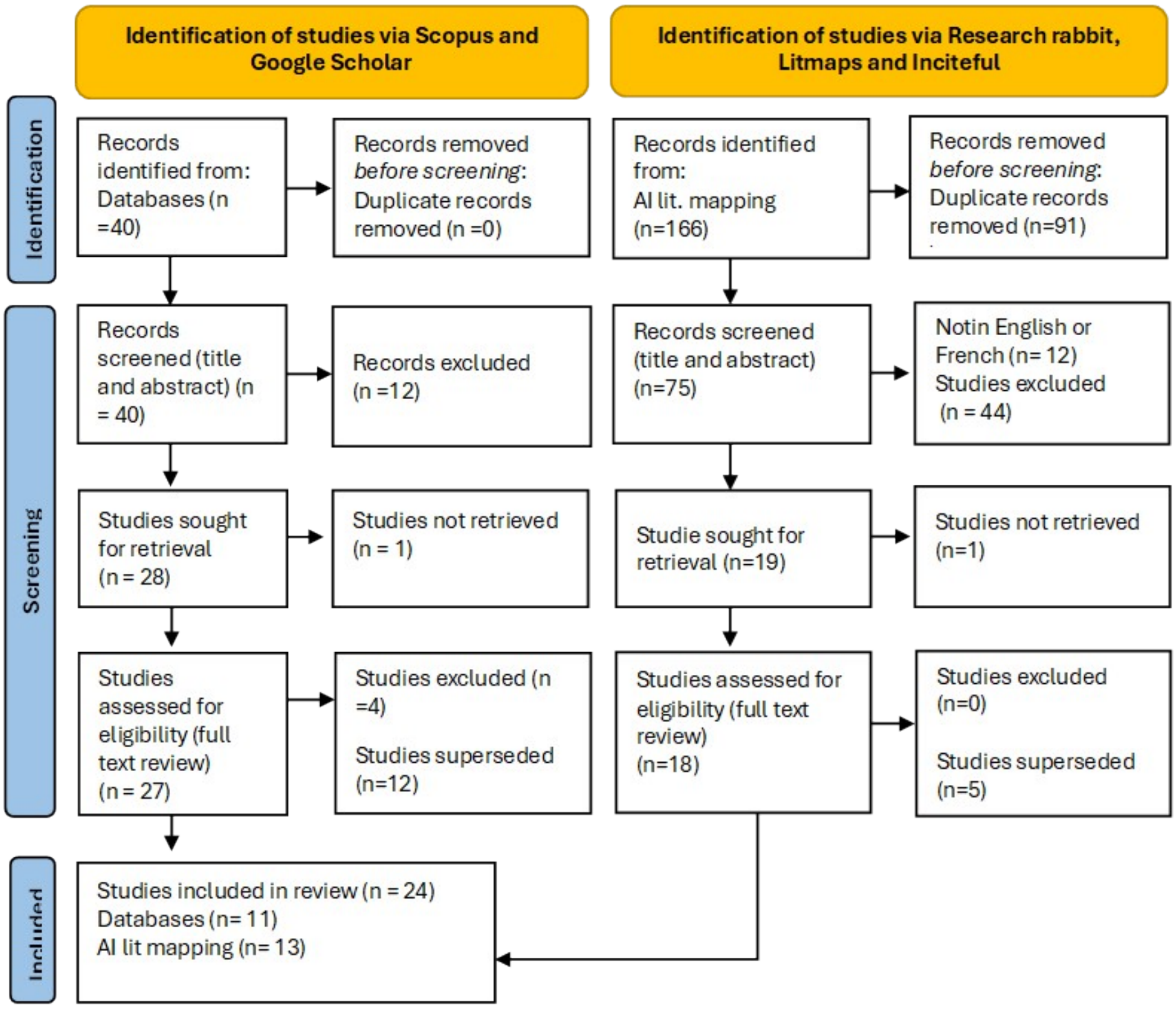

The scope of this review focuses on waste pickers and their enabling environment. It is recognised that there is a whole ecosystem linked to these waste pickers, which are required for the recycling value chain to operate. However, these are excluded from this review in order to remain as close as possible to the analogous sector of fecal sludge emptying and transport service providers. There are also wider issues which are excluded as unlikely to identify useful learning for regulating faecal sludge

emptying and transport, such as transboundary waste management. The health and safety issues faced by waste pickers are well documented and have many similarities to those faced by front line workers in fecal sludge emptying and transport. Literature describing these challenges is excluded from this review. Illegal waste disposal is not covered here but is incorporated to a parallel review on construction waste management.

The majority of the literature identified was focussed on waste picker cooperatives, largely within Latin America. Here ‘cooperative’ is used as a catch-all term including from associations to loose groupings of waste pickers. Brazil and Colombia are at the forefront of waste-picker community inclusion, with legal recognition and integration of waste-pickers as professionals after decades of political struggle within the national waste policy networks. Articles from Asia and Africa were included even where findings were not new to check for wider geographical relevance. Regulation and formalization for waste pickers appeared to be discussed interchangeably (Simpson et al., 2024).

A more limited second set of literature was identified which looked more widely at environmental crime and design of sanctions.

The rapid review findings incorporate 14 of the additional 16 recommended references from Expert feedback, with two duplicates removed. The preamble also includes further background on the limitations of the review in response to expert review comments.

A separate section summarises further reflections from the expert feedback on research and progress in the sector.

Findings

Target group: Motivations, Knowledge of the rules and capacity to comply. The importance of understanding the motives of regulated groups, and the diversity of these groups, was highlighted by many (Maiello, 2022). Regulated groups had a range of economic motives, some looking for greater job security rather than increased income. Alongside different perceptions and acceptance of risk, differing levels of need/desire for flexibility and autonomy were also reported (Aparcana, 2017; Kavuş et al., 2023). Social recognition, from others and workers recognizing

the importance of their own work, and status were also reported as a motivator and a major perceived benefit from cooperatives (Aparcana, 2017; Maiello, 2022; Kavuş et al., 2023).

Three approaches for engaging formal and informal workers in municipal solid waste management were given; (i) cooperatives, (ii) CBOS or MSEs, and (iii) engagement through the formal sector by larger waste management companies (Aparcana, 2017; Kain et al., 2022; Harfadli et al., 2024). The first two options require intensive participatory decision-making processes (Aparcana, 2017). Cooperatives are discussed further under 'External Actors'. CBOs or MSEs were noted as requiring less engagement from municipalities, but (and possibly linked to this) being less sustainable (Aparcana, 2017). Direct engagement through the formal sector tended to be non-participatory and transactional (Aparcana, 2017). These neat distinctions do not reflect how informal sector are often already intertwined with the formal sector in providing services (Simatele et al., 2017; Jiménez-Martínez, 2018; Muheirwe et al., 2024).

Dias and Samson (2016) present engagement as a ladder of integration, ranging from no integration at all to full integration. The following principles are presented: 1. Broad consultation and empowered participation, through waste pickers' organisations and representatives (Dias, 2016); 2. Economic support: Payment for waste pickers' labour and/or compensation for environmental service rendered; 3. Access to finance: credit lines for equipment, as well as micro-financing schemes; 4. Legal framework: proper contracts with payment for environmental services; laws and bylaws that recognize pickers as legitimate actors; 5. Work Conditions: provision of infrastructure for sorting/storage/processing; capacity building; social protection schemes (Dias and Samson, 2016).

Direct support was needed for organizational structures and individuals, for them to be successful in providing sustained services in, to or through low-income groups. The main areas of support identified were; social mobilization and acceptance, financial and operational support and policy and institutional arrangements (Aparcana, 2017; Gómez-Maldonado et al., 2023; da Silva et al., 2024; Harfadli et al., 2024). More specific examples included; grant funding, business development and support to reach cost recovery, training, facilities (space, energy or water

supply), equipment, support in meeting health and safety standards for workers and support in completing bureaucratic processes (Calderón Márquez et al., 2021; Kain et al., 2022; Gómez-Maldonado et al., 2023; da Silva et al., 2024). It was recommended that training be adapted to the context and provided largely as on the job training and mentoring, with the content responding to the issues faced in practice and the demands of the trainees (Ibelli-Bianco et al., 2022).

There are also examples where grassroot innovation has been able to develop technical, organizational and socioeconomic solutions, and thereby provide services where formal bodies have failed to do so (Zapata Campos and Zapata, 2013; Zapata Campos et al., 2023a&b). Zapata Campos et al. (2023a) refer to 'extreme' niches of innovation in the resource-poor 'cracks' of formal systems. Zapata Campos et al. (2023b) highlights the importance of development, community relationships and digital innovation framing for inspiring and motivating participation, and the role of the enabling environment in sustaining grassroots innovations in the long term.

The existing capacity and specialised skills of waste pickers, and their role as important stakeholders who contribute positively to the environment and local economy, also needs to be recognized (Aparcana, 2017; Kavuş et al., 2023). Successful waste-picker incorporation projects have paired confidence in the capacity of informal workers, along with the recognition that addressing marginalization require pro-active programmes of investment and capacity building (Tucker, 2024; Aparcana, 2017). The challenges in obtaining and retaining this recognition, given the vulnerability and stigmatization of waste pickers, cannot be overemphasized (Aparcana, 2017; Tucker, 2024; Simatele et al., 2017). These challenges are present for both the waste pickers and their organisations. National or global waste pickers federations and movements have been important to prompt societal awareness of rights and demands of informal workers, and to strengthen their political influence through collective action (Kain et al., 2022; Simpson et al., 2024).

Economic viability was a common theme through the literature, including diversification of income streams for workers or organisations, and approaches to guarantee a stable minimum wage for workers, such as financial mechanisms,

development of payment schemes, and the strengthening of their business capacities (Aparcana, 2017).

Resourcing and supporting regulators / Perceptions of, and relationship with, the regulator. Higher levels of local governmental support can improve the sustainable performance of waste-pickers in collection rates, salaries and working conditions (Aparcana, 2017; Navarrete-Hernández and Navarrete-Hernández, 2018). Participatory approaches and involvement in developing the formalization process are a common success factor (Aparcana, 2017). The most persistent barriers post formalization are related to a lack of leadership and trust between cooperatives and regulators and within cooperatives (Aparcana, 2017).

Municipal governments need to be resourced to work closely with and support cooperatives and other structures (Calderón Márquez et al., 2021; Kavuş et al., 2023; Simpson et al., 2024). A decentralized waste management system that empowers municipalities with additional responsibilities for waste management and treatment could be beneficial for improving local control (Andreatta et al., 2023). Pro-poor policy change will also require an epistemic shift to value waste pickers as essential urban workers (Tucker, 2024).

The importance of a credible threat of enforcement is noted (Muheirwe et al., 2022; Germani, 2024). Fines for illegal dumping of municipal waste need to be significantly higher than the cost of safe disposal (Muheirwe et al., 2022). However, D’Amato et al. (2018) highlight that threatening sanctions that you cannot enforce can have the perverse impact of reducing voluntary compliance.

Enabling Environment. Informal waste pickers were seen for many years as a temporary phenomenon, now many governments are acknowledging their positive contribution and have begun integrating them in formal waste management systems (Aparcana, 2017; Muheirwe et al., 2022; Koen and Fourie, 2023; Simpson et al., 2024). Muheirwe et al. (2024) and Simatele et al. (2017) highlight that across sub-Saharan Africa regulations are typically limited to formal actors, not considering the reality of national economies and making basic service provision effectively illegal.

The feasibility and applicability of policies needed to be reviewed before and during implementation. A number of examples were given of policies which, largely

unintentionally, were not possible for low-capacity groups to comply with (Dlamini, 2016; Muheirwe et al., 2024). Examples include requiring security checks (where many informal workers had a history of being criminalized due to the nature of their work), registered addresses and complex reporting requirements (Kavuş et al., 2023; Koen and Fourie, 2023). Mandatory membership of cooperatives, with associated costs for membership which were unaffordable for many, both increased non-compliance and were perceived as repressive (Muheirwe et al., 2022). Transport of waste requirements sometimes came under regulations intended for larger commercial companies, not appropriate for waste picker trolleys (Koen and Fourie, 2023; Muheirwe et al., 2024). The informal sector may have been engaged with the formal sector for many years and have established ways of working, which can be disrupted by formalisation and led to further marginalisation (Dlamini, 2016; Muheirwe et al., 2024).

In some locations waste picking is banned but continues, with tacit agreement from local municipality, to contribute to recycling, and livelihoods (Simatele et al., 2017; Koen and Fourie, 2023). Even when permitted in practice, legal bans on waste picking exposes these workers to harassment and is a symbol of societal stigma (Koen and Fourie, 2023; Tucker, 2024). Local bylaws can continue to criminalise waste pickers even where it is permitted under national regulations (Koen and Fourie, 2023).

Regulations need to be adapted to local contexts (Dlamini, 2016; Aparcana, 2017; Calderón Márquez et al., 2021; Muheirwe et al., 2022; Koen and Fourie, 2023; Harfadli et al., 2024). Informal settlements are unique and therefore may require special rules and regulations (Muheirwe et al., 2022). Policies need to understand the historical production of waste-picker organizing styles and appreciate the diversity of groups of informal recyclers (Tucker, 2024; Harfadli et al., 2024).

Jiménez-Martínez (2018) describes how this paradox, where the informal sector is essential to basic service delivery but is officially banned, is due to dualist approach where formal and informal are seen as distinct and separate. They describe how a structuralist approach may be more helpful, where formal and informal are seen as intertwined and operating along a continuum, "*expressed in a variety of practices, at*

different spheres of society and diverse contexts, and which involves a diversity of people, practices, strategies and modalities".

External actors

Neighbourhood-watch schemes, where the public monitor illegal disposal of municipal solid waste, have been introduced in some locations (Muheirwe et al., 2022; Andreatta et al., 2023). NGOs and international organisations have been influential in initiating change and changing how the informal sector is viewed (Apacarna, 2017; Calderón Márquez et al., 2021; Simpson et al., 2024).

Cooperatives (or other Membership Based Organisations): Cooperatives have been shown to have a positive impact on the productivity and quality of life of waste pickers across Latin America, Africa and Asia (Dias and Samson, 2016; Chen et al., 2018; Calderón Márquez et al., 2021; Simpson et al., 2024). Waste picker cooperatives can support better working conditions, recognition, reduction of stigma and co-regulation (Dias and Samson, 2016; Aparcana, 2017; Colombijn and Morbidini, 2017; Dias and Fernández, 2020; Coletto and Carbonai, 2023; Simpson et al., 2024). Cooperatives have been integrated into waste management schemes and have been able to shape alternative routes for formalisation and employment alongside a social support function for members (Dias and Samson, 2016; Dias and Fernández, 2020). Research has suggested that cooperatives have the potential to contribute more to the development of the sector, if properly supported and with platforms through which they can influence policy (Samson, 2009, p.86; Aparcana, 2017; da Silva et al., 2019; Carenzo, 2020; Calderón Márquez et al., 2021; Harfadli et al., 2024). However, the strength of the cooperatives depends on its location and the level of decentralisation in decision making, among many other factors; smaller cities may have weaker cooperatives or less policy leverage (Gómez-Maldonado, et al., 2023).

Not all waste pickers want to be incorporated into cooperatives, it depends on their relationship with the state and the balance of different motives for the individual (Colombijn and Morbidini, 2017; Tucker, 2024). Kasinja and Tilley (2018) highlight that concerns of free riding behaviours, conflict and decreased income can be a barrier to informal waste pickers forming cooperatives. Cooperatives can shield workers from risk, raise their social profile, and provide guaranteed income.

However, they may lead to stricter governmental control and limited flexibility and choice.

Cooperatives may be particularly important for vulnerable groups. Socially excluded people may trust and value cooperatives more, indicating the importance of this structure in the formalisation process (Çolak et al., 2024). Some studies have demonstrated how cooperatives can be a route for women waste pickers to fight gender-based vulnerabilities and take leadership roles (Dias and Ogando, 2015ab).

The above discussion presents integration as being waste pickers being integrated into municipal systems. Samson (2020) critiques this as being also a process of erasure highlights how change can be transformative when the focus shifts to the integration of municipalities and industry into systems led by front line service providers. Cavé (2020) presents consideration of waste as a common pool resource as another approach to change how actors work and share use rights.

Reflections from Expert feedback (Four No. Responses)

The discussion on risks, vulnerability, and lack of social protection that informal waste workers experience is a key issue depicted in academic and grey literature. Waste pickers face health risks including the handling of hazardous chemical and biological materials, insalubrious workplace conditions, propensity for accidents, the lack of use of protective equipment, the lack of proper sanitary conditions, problems with organizational processes, and emotional and gender-based vulnerabilities.

The challenges linked to the vulnerability and stigmatization of waste picker organizations, especially those that are more informally organized, should be highlighted. Membership based organisations are critical in protecting vulnerable groups. Organizing can provide paths for women to fight gender-based vulnerabilities and purse greater dignity.

Apparently well-functioning collaboration between local authorities, waste pickers and households can be quickly abandoned with changes in local leadership or in the political climate. Such changes can be due to the introduction of large-scale waste management systems, or a push for formalization. In these situations, there may be little consideration of the '*devastating socioeconomic repercussions for the waste pickers*'. There are examples of this reaction when outside funding arrives to

implement large-scale waste management systems or other urban renewal initiatives, resulting in '*the abandonment of informal waste pickers to the benefit of more formal sectors*'.

References

Andreatta, D., Favarin, S., Lisciandra, M., & Millemaci, E. (2023). Digging into waste: an analysis of waste crime in the Italian provinces. *Regional Studies*, 57(7), 1367-1379.

Aparcana, S. (2017). Approaches to formalization of the informal waste sector into municipal solid waste management systems in low-and middle-income countries: Review of barriers and success factors. *Waste management*, 61, 593-607.

Calderón Márquez, A. J., Silva de Souza Lima Cano, N., & Rutkowski, E. W. (2021). Inclusion of waste pickers into municipal waste management systems: A comparison between Colombia and Brazil. *The Journal of Environment & Development*, 30(4), 395-425.

Carenzo, S. (2020). Contesting informality through innovation "from below": epistemic and political challenges in a waste pickers cooperative from Buenos Aires (Argentina). *Tapuya: Latin American Science, Technology and Society*, 3(1), 441-471.

Cavé, J. (2020) Managing urban waste as a common pool resource. In: *The Informal Economy Revisited: Examining the Past, Envisioning the Future.* (M. Chen & F. Carré, Eds.). Women in Informal Employment: Globalizing and Organizing (WIEGO). Cambridge, USA.

Chen, F., Luo, Z., Yang, Y., Liu, G. J., & Ma, J. (2018). Enhancing municipal solid waste recycling through reorganizing waste pickers: A case study in Nanjing, China. *Waste Management & Research*, 36(9), 767-778.

Çolak, H., Yiğit, M., Boyaci, N. B., Şişman, Y., Kağnicioğlu, D., & Kağnicioğlu, C. H. (2024). Do not waste "Pickers": exploring the intention to join waste picker cooperatives. *Journal of Material Cycles and Waste Management*, 26(6), 3482-3494.

Coletto, D., & Carbonai, D. (2023). What Does it Mean to Have a Dirty and Informal Job? The Case of Waste Pickers in the Rio Grande do Sul, Brazil. *Sustainability,* 15(3), 2337.

Colombijn, F., & Morbidini, M. (2017). Pros and cons of the formation of waste-pickers' cooperatives: a comparison between Brazil and Indonesia. *Decision,* 44, 91-101.

D'Amato, A., Mazzanti, M., Nicolli, F., & Zoli, M. (2018). Illegal waste disposal: Enforcement actions and decentralized environmental policy. Socio-economic planning sciences, 64, 56-65.

da Silva, C. L., Weins, N., & Potinkara, M. (2019). Formalizing the informal? A perspective on informal waste management in the BRICS through the lens of institutional economics. *Waste Management*, 99, 79-89.

da Silva, C.L., Guabiroba, R. C., Jacobi, P. R., Besen, G. R., & da Silva, M. A. V. (2024). Sustainability performance indicators: improving waste picker organizations in a Brazilian region. *Journal of Material Cycles and Waste Management*, 26(6), 3946-3961.

Dias, S. M. & Fernández, L. (2020). Formalisation from the ground: the case of waste pickers' cooperatives. In *Research handbook on development and the informal economy* (pp. 263-284). Edward Elgar Publishing.

Dias, S.M. & Ogando, A. C. (2015a). Engendering Waste Pickers Cooperatives in Brazil. In *ILO Research Conference – Antalya*, 9-10 November 2015 (pp. 9–10). Antalya.

Dias, S. M. & Ogando, A. C. (2015b). Rethinking gender and waste: exploratory findings from participatory action research in Brazil. *Work Organisation, Labour & Globalisation*, 9(2), 51–63.

Dias, S. M. & Samson, M. (2016). Informal Economy Monitoring Study Sector Report: Waste Pickers. (Vol. 1). Women in Informal Employment: Globalizing and Organizing (WIEGO). Cambridge, USA. Retrieved from http://www.wiego.org/sites/default/files/publications/files/Dias-Samson-IEMS-Waste-Picker-Sector-Report.pdf (accessed 24 June 2025)

Dlamini, S. Q. (2016). Solid waste management in South Africa: exploring the role of the informal sector in solid waste recycling in Johannesburg. (PhD Thesis). Faculty of Science, School of Geography, University of the Witwatersrand, Witwatersrand: Johannesburg.lands.

Germani, A. R., Castaldo, A., & Ker, A. (2024). Carrots, sticks, and environmental crime in Italy. *European Journal of Law and Economics*, 58(2), 201-219.

Gómez-Maldonado, A., Ospina-Espita, L. C., Rodríguez-Lesmes, P., & Rodríguez-Rodriguez, M. A. (2023). Barriers and opportunities for waste pickers within solid waste management policy in Colombia. *Waste Management*, 163, 1-11.

Harfadli, M. M. A., Ramadan, B. S., Rachman, I., & Matsumoto, T. (2024). Challenges and characteristics of the informal waste sector in developing countries: an overview. *Journal of Material Cycles and Waste Management*, 26(3), 1294-1309.

Ibelli-Bianco, C., Guimarães, J. P. S., Yamane, L. H., & Siman, R. R. (2022). Education and training: Key solution to self-management and economic sustainability of waste pickers organisations. *Waste Management & Research*, 40(10), 1505-1513.

Jiménez-Martínez, N. M. (2018). The governance of waste: Formal and informal rules in the central region of Mexico. *Regional Studies, Regional Science*, 5(1), 353–360.

Kain, J. H., Zapata, P., Mantovani Martiniano de Azevedo, A., Carenzo, S., Charles, G., Gutberlet, J., ... & Zapata Campos, M. J. (2022). Characteristics, challenges and innovations of waste picker organizations: A comparative perspective between Latin American and East African countries. *Plos one*, 17(7), e0265889.

Kasinja, C., & Tilley, E. (2018). Formalisation of informal waste pickers' cooperatives in Blantyre, Malawi: A feasibility assessment. *Sustainability*, 10(4), 1149.

Kavuş, H. K., Erköse, Y., & Eryar, D. (2023). Driving Green Job Opportunities in Sustainable Waste Management through Co-Production Strategies: Informal Recycling Workers, Municipalities, and the National Agenda—A Case Study of İzmir. *Social Sciences*, 12(7), 387.

Koen, L., & Fourie, E. S. (2023). Waste pickers and the law: Contradictory and fragmented regulation in three metropolitan municipalities. *Development Southern Africa*, 40(2), 373-389.

Maiello, A. (2022). In, Out or Beyond? Waste Pickers and Policy Networks: A Story from Jardim Gramacho (Rio de Janeiro). *Sustainability*, 14(24), 16977.

Muheirwe, F., Kombe, W., & Kihila, J. M. (2022). The paradox of solid waste management: A regulatory discourse from Sub-Saharan Africa. *Habitat International*, 119, 102491.

Muheirwe, F., Kombe, W. J., & Kihila, J. M. (2024). Solid waste collection in the informal settlements of African cities: a regulatory dilemma for actor's participation and collaboration in Kampala. In *Urban Forum* (Vol. 35, No. 1, pp. 1-22). Dordrecht: Springer Netherlands.

Navarrete-Hernández, P., & Navarrete-Hernández, N. (2018). Unleashing waste-pickers' potential: supporting recycling cooperatives in Santiago de Chile. *World Development*, 101, 293-310.

Samson, M. (2009). *Refusing to be Cast Aside: Waste Pickers Organising Around the World*. Waste Pickers Organising Around the World. Women in Informal Employment: Globalizing and Organizing (WIEGO). Cambridge, USA.

Samson, M. (2020). The political work of waste picker integration. In: *The Informal Economy Revisited: Examining the Past, Envisioning the Future.* (M. Chen & F. Carré, Eds.). Women in Informal Employment: Globalizing and Organizing (WIEGO). Cambridge, USA.

Simpson, M., Oduro-Appiah, K., Gunsilius, E., Dias, S. M., & Scheinberg, A. (2024). Shifting perceptions of informal operators in the service and value chains: A retrospective of 40 years of observation and advocacy for informal recyclers and waste service providers, through the eyes of five global participant-researchers. *Waste Management & Research,* 0734242X241280076.

Simatele, D. M., Dlamini, S., & Kubanza, N. S. (2017). From informality to formality: Perspectives on the challenges of integrating solid waste management into the

urban development and planning policy in Johannesburg, South Africa. *Habitat International*, 63, 122–130.

Tucker, J. L. (2024). Barriers to Inclusive Recycling in Asunción, Paraguay: A Just Transition?. *Development and Change*, 55(2), 276-301.

Zapata Campos, M. J., & Zapata, P. (2013). Switching Managua on! Connecting informal settlements to the formal city through household waste collection. *Environment and Urbanization*, 25(1), 225–242.

Zapata Campos, M. J., Carenzo, S., Charles, G., Gutberlet, J., Kain, J. H., Oloko, M. O., ... & Zapata, P. (2023a). Grassroots innovations in 'extreme' urban environments. The inclusive recycling movement. *Environment and Planning C: Politics and Space*, *41*(2), 351-374.

Zapata Campos, M. J., Barinaga, E., Dimba Kiaka, R., & Ocampo, J. (2023b). Nothing to lose: the rationales of grassroots financial innovations in contexts of extreme scarcity. *Social Enterprise Journal*, *19*(2), 193-212.
